# Atomic Charge Dependency of Spiropyran/Merocyanine Adsorption as a Precursor to Surface Isomerization Reactions

**DOI:** 10.1021/acsomega.3c06712

**Published:** 2023-12-28

**Authors:** Andreas Riemann, Lauren Rankin, Dylan Henry

**Affiliations:** Department of Physics & Astronomy, Western Washington University, 516 High Street, Bellingham, Washington 98225, United States

## Abstract

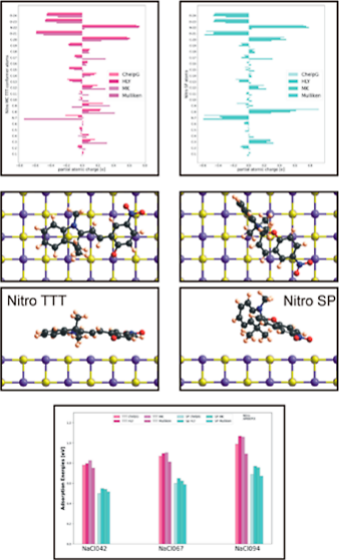

This computational study investigates the adsorption of various spiropyran and merocyanine isomers on a NaCl substrate using a combination of density functional theory (DFT) and molecular mechanics (MM) calculations. Four different charge methods were used to determine the partial atomic charges for the adsorbate molecules, including Mulliken population analysis and three electrostatic potential (ESP) methods (Merz–Kollman, ChelpG, and Hu-Lu-Yang), while three different force fields (AMBER 3, CHARMM 27, and MM+) were employed for the MM calculations. The results show that the various DFT charge methods produced similar outcomes for the molecules’ partial atomic charges, with some exceptions for individual atoms and methods. Additionally, it was found that the ESP charge methods were more sensitive to the conformer orientation than the Mulliken approach. The adsorption behavior of merocyanine conformers with the central bond in *trans* orientation (T-conformers) was similar for various configurations, with the molecule adsorbing mostly flat with its aromatic rings almost parallel to the substrate. However, C-conformers (with their central bond in *cis* orientation) and spiropyran isomers exhibited inconsistent adsorption behavior, mostly because only some of the aromatic rings contributed to the adsorption behavior. Due to additional van der Waals interactions of more aromatic rings, the adsorption energies for T-conformers are consistently 0.2–0.3 eV higher than for C-conformers and for spiropyran. The study found that the adsorption geometries and energies of stable T-conformers were independent of the partial atomic charge scheme and force field used, and C-conformers show parameter-dependent behavior upon adsorption, leading to metastable configurations. These findings indicate viable pathways during the spiropyran-merocyanine isomerization reactions. Therefore, the results provide initial insights into the possibility of switching spiropyran isomers into merocyanine isomers and vice versa after adsorption onto substrates.

## Introduction

Molecular mechanics (MM) calculations offer powerful tools to explore adsorption geometries, energies, and film growth parameters.^1−11^ Various force fields can be utilized for different combinations of adsorbates and substrates.^12−15^ These MM calculations can be advantageous over purely quantum chemistry approaches due to requirements of less computational power and therefore faster speeds. Obviously, this requires approximation schemes that are suitable for the investigated system.

Studies of the adsorption of molecules on ionic insulator substrates have been of significant interest for a number of adsorbate/substrate combinations.^16,17^ Here, we chose to investigate various spiropyran/merocyanine molecules upon their adsorption on an insulating NaCl template. This system was chosen for its rich complexity of molecular adsorbates, together with a polar substrate. To study the performance of MM calculations, different merocyanine molecules, each with different conformers, can be utilized and compared. One of the important “ingredients” for MM are the charge distributions within a molecule. In order to study several configurations, these molecules can be optimized by using quantum chemistry calculations and various charge schemes. This approach was chosen to carefully probe the effect of assigning and calculating partial atomic charges for molecules, especially investigating the difference between Mulliken population analysis and methods using electrostatic potentials (ESPs).

This investigation does not replace a rigorous ab initio quantum mechanical approach, such as density functional theory (DFT); however, it provides a suitable approximation scheme for a larger application field with less computational requirement using MM. The calculations obtained concerning adsorption geometries and energies provide qualitative and quantitative results to evaluate subsequent isomerization processes for the spiropyran/merocyanine class of molecular switches. This class of molecules can undergo reversible isomerization caused by a variety of stimuli depending on the environment of the molecule. The switching between these isomers has been investigated extensively in solution and only recently on selected substrates.^18−22^ The class of spiropyran-based molecules is especially interesting since the isomers have vastly different properties concerning the size, dipole moment, color, and emission properties. Both isomers are structurally very distinguishable: spiropyran is a three-dimensional molecule where the spiro junction leads to aromatic rings being at 90° angles. However, merocyanine with the central C–O bond broken is a much more planar molecule. These properties play an important role in their adsorption behavior when anchored to a substrate. The charge separation within the molecule leads to pronounced dipole moments in both the spiropyran and merocyanine isomers.

The investigated molecules acting as molecular switches can be used as building blocks for molecular electronics, where the adsorption properties of molecular thin films are an important first step in developing functional devices.^23,24^ Furthermore, decoupling from substrate properties will help isolate its functionality. Therefore, an insulating substrate such as NaCl was chosen to investigate the system. An additional benefit of the chosen surface template is its well-known electronic properties and subsequently the ionic character and charge distribution, making it a preferred candidate for MM calculations.

## Methods

As the substrate for this investigation, we chose a double layer of NaCl. There are many investigations of molecular adsorption on sodium chloride films deposited on a metal template and other substrates.^25−35^ This system can be investigated, e.g., with powerful experimental methods, such as STM and AFM, in order to study single-molecule behavior on insulating films and electronic exchange interactions. The NaCl film was created using a well-established lattice parameter (*a*_0_ = 5.64 Å).^36^ The local charges on the individual ions compromising the film were chosen according to three methods. The first choice of ±0.67*e* is according to Pauling method,^37^ the second choice of ±0.42*e* using the Planewave method,^38^ and last, a charge of ±0.94*e* according to DDEC3.^39^ In order to avoid the so-called edge effects and keep computational resources manageable, the substrate consisted of two layers of 32 × 32 atoms, for a total of 2048 atoms. The dimensions of the template are therefore 91 Å × 91 Å × 2.84 Å. In a previous study, we have shown that a potential increase in substrate size would only results in changes to the adsorption energies which are smaller than the convergence criteria of 0.001 kcal/mol used for MM.^40^ Additionally, throughout the computation, the NaCl substrate is treated as “frozen”, i.e., interatomic distances remain unchanged. This configuration would mimic a bulk NaCl crystal more closely, so that this study is more widely applicable for different scenarios. However, if the surface would be allowed to relax, previous DFT studies have shown interlayer buckling of the substrate upon atomic adsorption, especially in the first two layers of NaCl.^29,41^ The computational cost of allowing the substrate to relax for the wide parameter space of this study is too high in the present case, but we carried out some calculations in which the substrate is allowed to relax upon molecular adsorption of merocyanine and spiropyran. The effect observed is an expected increase in adsorption energy of about 7.5–9.5% for the investigated subset of data, with the same overall quantitative results of the study using a “frozen” substrate, validating the approach of our study only using a “frozen” substrate for all subsequent calculations.

**Figure 1:**
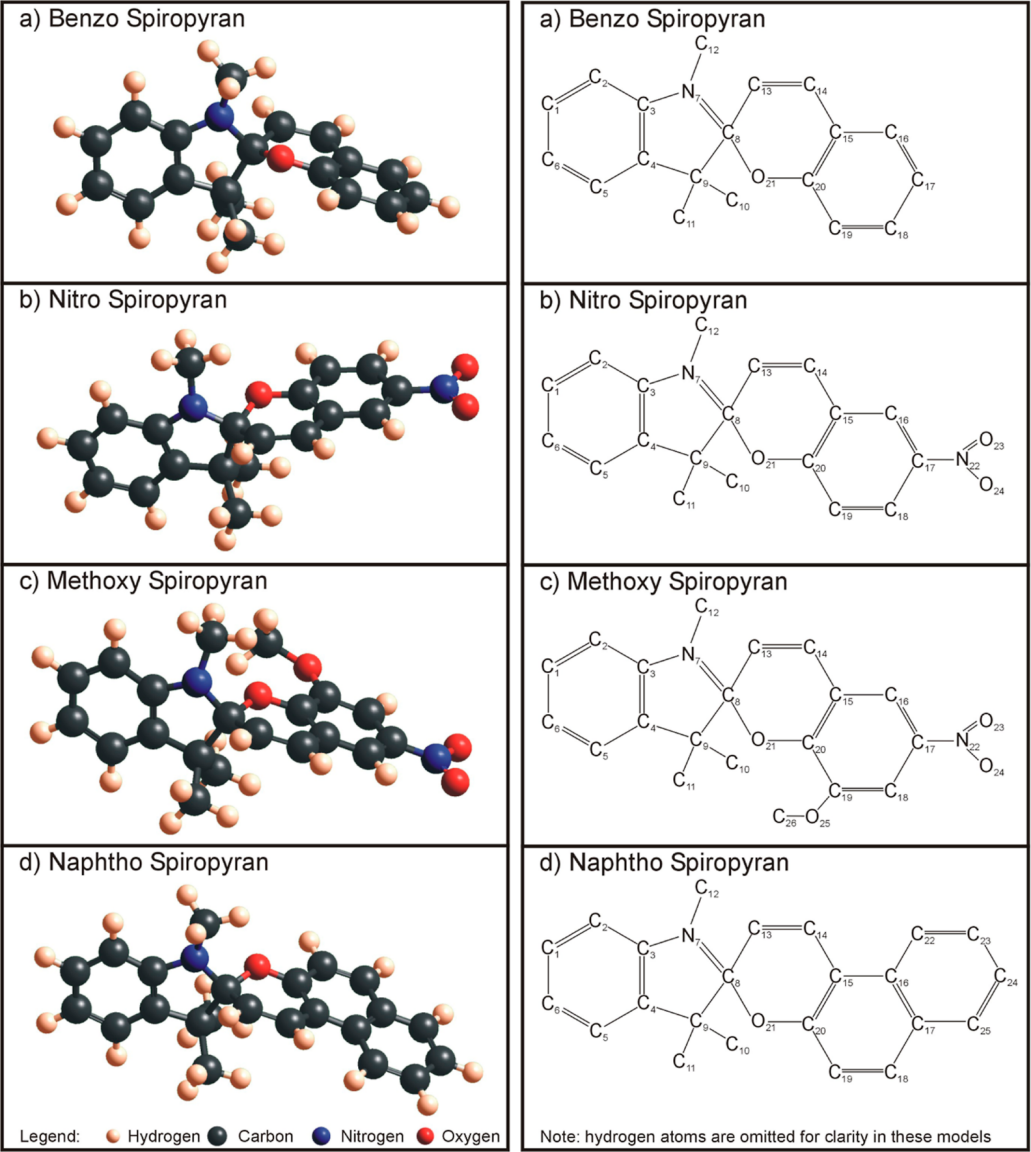
Four investigated molecules as spiropyran isomers, in a ball-and-stick model (left) and with labeled atom numbers in the Kekulé structure (right) without omitted hydrogen atoms, for clarity. (a) Benzo spiropyran = 1,3,3-trimethylindolinobenzopyrylospiran, (b) nitro spiropyran = 1,3,3-trimethylindolino-6′-nitrobenzopyrylospiran, (c) methoxy spiropyran = 1′,3′-dihydro-8-methoxy-1′,3′,3′-trimethyl-6-nitrospiro[2*H*-1-benzopyran-2,2′-[2H]indole], and (d) naphtho spiropyran = 1,3,3-trimethylindolino-β-naphthopyrylospiran.

**Figure 2:**
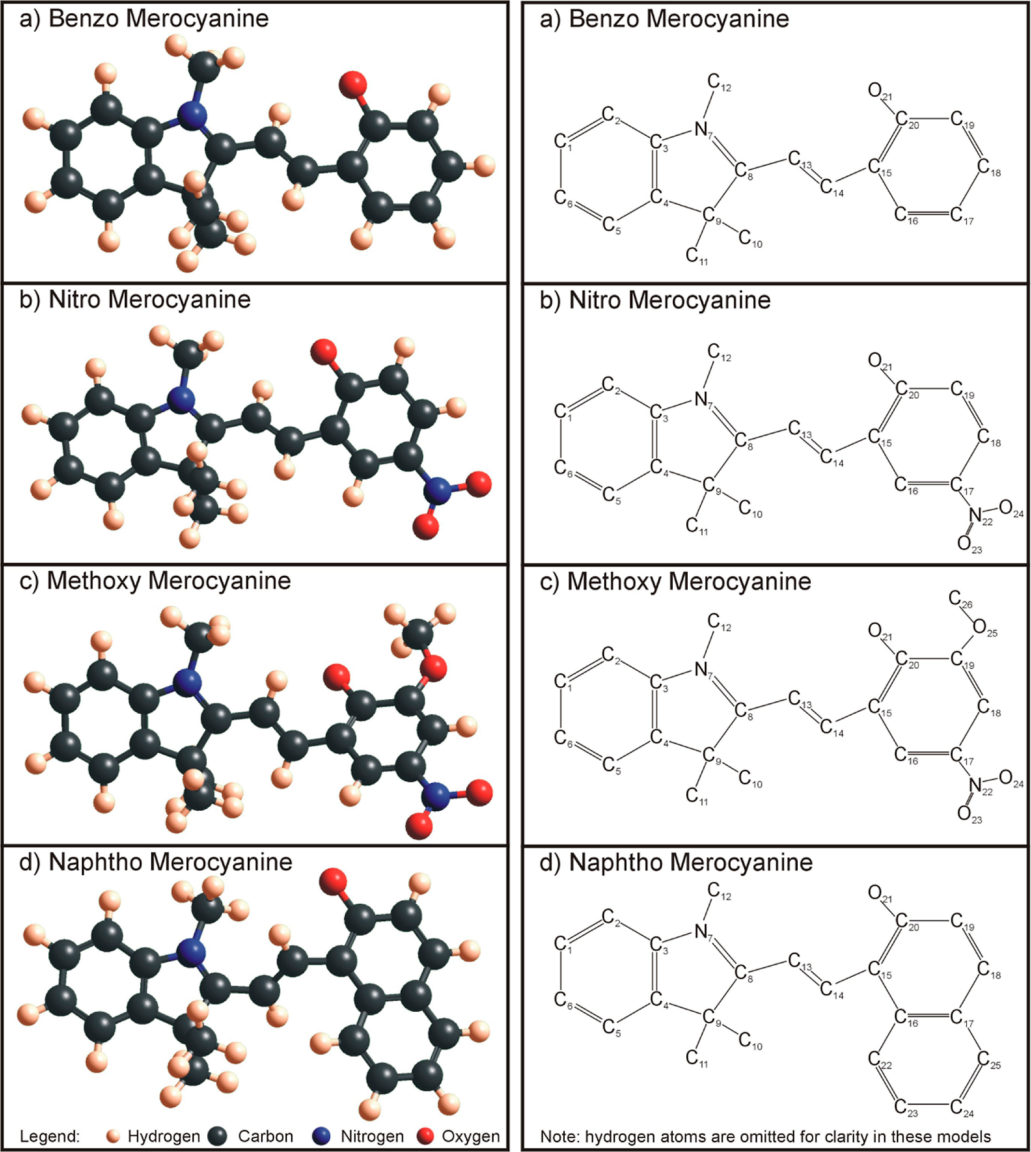
Pictured are the four investigated molecules as merocyanine isomers in the ball-and-stick model (left) and with labeled atom numbers in the Kekulé structure (right) without omitted hydrogen atoms, for clarity. (a) Benzo merocyanine isomer of 1,3,3-trimethylindolinobenzopyrylospiran, (b) nitro merocyanine isomer of 1,3,3-trimethylindolino-6′-nitrobenzopyrylospiran, (c) methoxy merocyanine isomer of 1′,3′-dihydro-8-methoxy-1′,3′,3′-trimethyl-6-nitrospiro[2*H*-1-benzopyran-2,2′-[2*H*]indole], and (d) naphtho merocyanine isomer of 1,3,3-trimethylindolino-β-naphthopyrylospiran.

**Figure 3:**
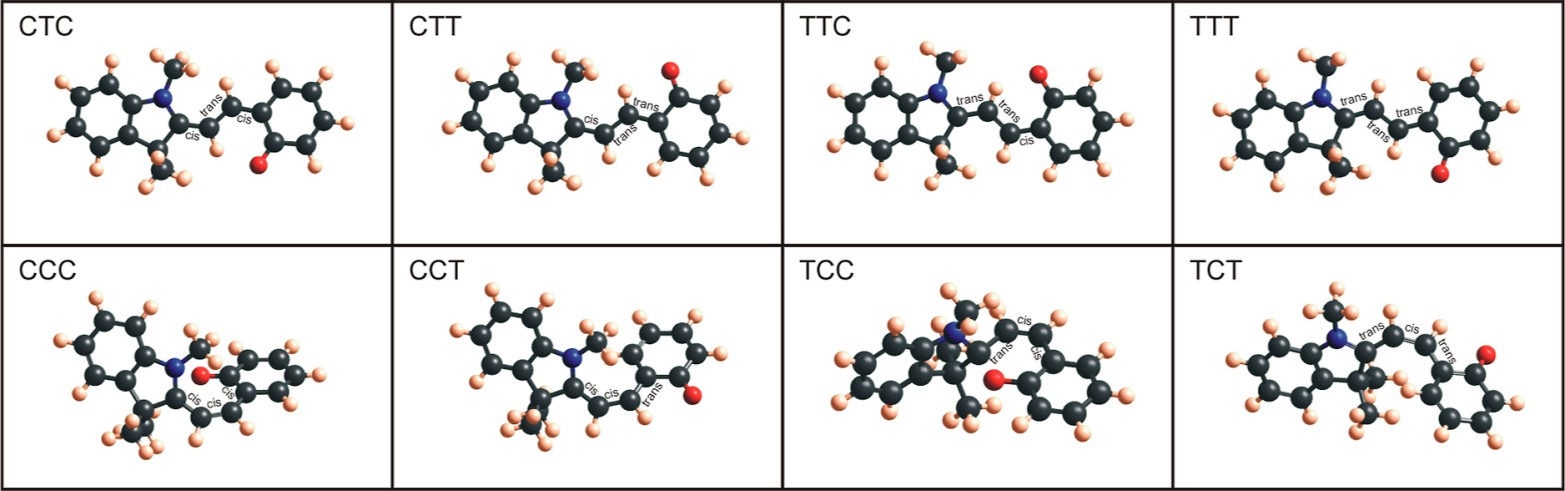
Naming scheme for merocyanine conformers (shown here is an example of Benzo-MC isomers). Conformers with a central C–C bond in the *trans* configuration (top row) are mostly planar. Conformers with a central C–C bond in *cis* configuration (bottom row) are not planar with aromatic rings at various angles.

As adsorbates, four different merocyanine/spiropyran molecules were used: (i) a benzo-merocyanine (Benzo-MC), isomer to spiropyran 1′,3′,3′-trimethylspiro[1(2*H*)-benzopyran-2,2′-indoline] (C_19_ H_19_ NO—Figures 1a and 2a), (ii) a nitro-merocyanine (Nitro-MC), isomer to spiropyran 1′,3′,3′-trimethyl-6-nitrospiro[1(2*H*)-benzopyran-2,2′-indoline] (C_19_ H_18_ N_2_ O_3_—Figures 1b and 2b), (iii) a methoxy-merocyanine (Methoxy-MC), isomer to spiropyran 1′,3′-dihydro-8-methoxy-1′,3′,3′-trimethyl-6-nitrospiro[2*H*-1-benzopyran-2,2′-[2*H*]indole] (C_20_ H_20_ N_2_ O_4_—Figures 1c and 2c), and (iv) a naphtho-merocyanine (Naphtho-MC), isomer to spiropyran 1,3,3-trimethylspiro[indoline-2,3′-[3*H*]naphth[2,1-*b*]pyran] (C_23_ H_21_ NO—Figures 1d and 2d). The merocyanine molecules can be found in eight conformers according to their three central carbon bonds being either in *trans* or in *cis* orientation. The naming scheme of the conformers is established according to the three central bond configurations using the nitrogen in the pyrrole ring and the oxygen directly attached to the benzene ring as the start and end points, respectively (see Figure 3). The choice of four different SP/MC molecules encompasses a wide range of side groups (naphtho rings, methoxy groups, and nitrite groups) added onto the Benzo-SP/MC molecule to broaden the scope of the present investigation. These four compounds are also commercially available at TCI Chemicals.

All molecules and their conformers were optimized in Gaussian with the DFT-B3LYP method and a 6-31G basis set.^42^ The self-consistent field (SCF) convergence was set to the default for Gaussian as SCF = Tight, leading to an energy convergence of 1.00D-06. We employed the B3LYP method due to its widely recognized high quality for molecular chemistry calculations.^43^ After optimization, the atomic partial charges on all atoms in the molecule were calculated either by Mulliken population analysis or by determining the charges from the ESP according to the chelpG approach, the HLY method, and the Merz–Kollman (MK) method. For each conformer, the results are one geometry-optimized configuration with four different charge schemes. The labeling/numbering of the atoms in each MC/SP molecule can be found in the schematics on the right side of Figures 1 and 2. Starting on the benzene ring on the right, all the four molecules have consistent numbering up to O-21.

The DFT-optimized molecules were the starting point of the calculations with MM using HyperChem 8.0. As force fields for the MM calculations, three common approaches were employed: AMBER 3, CHARMM27, and the universal force field MM+. The molecules were again geometry-optimized with MM taking their partial atomic charges into consideration, and a baseline energy for each molecule and conformer was obtained. In a subsequent step, these MM-optimized molecules were used for adsorption on the different substrate configurations. Here, the molecules were positioned close to the substrate (at a consistent height of 5 Å with their center of mass above the substrate) and allowed us to find the optimal position using Polak-Ribiere energy gradient calculation in HyperChem with a convergence limit of 0.001 kcal/mol.^44^ Adsorption energies were determined as the difference in energy for the substrate/adsorbate system between the situation where the molecule is very far away (200 Å above the NaCl layers) and when the molecule is in its optimal configuration on top of the substrate.

## Results and Discussion

### Charge Calculations

**Figure 4:**
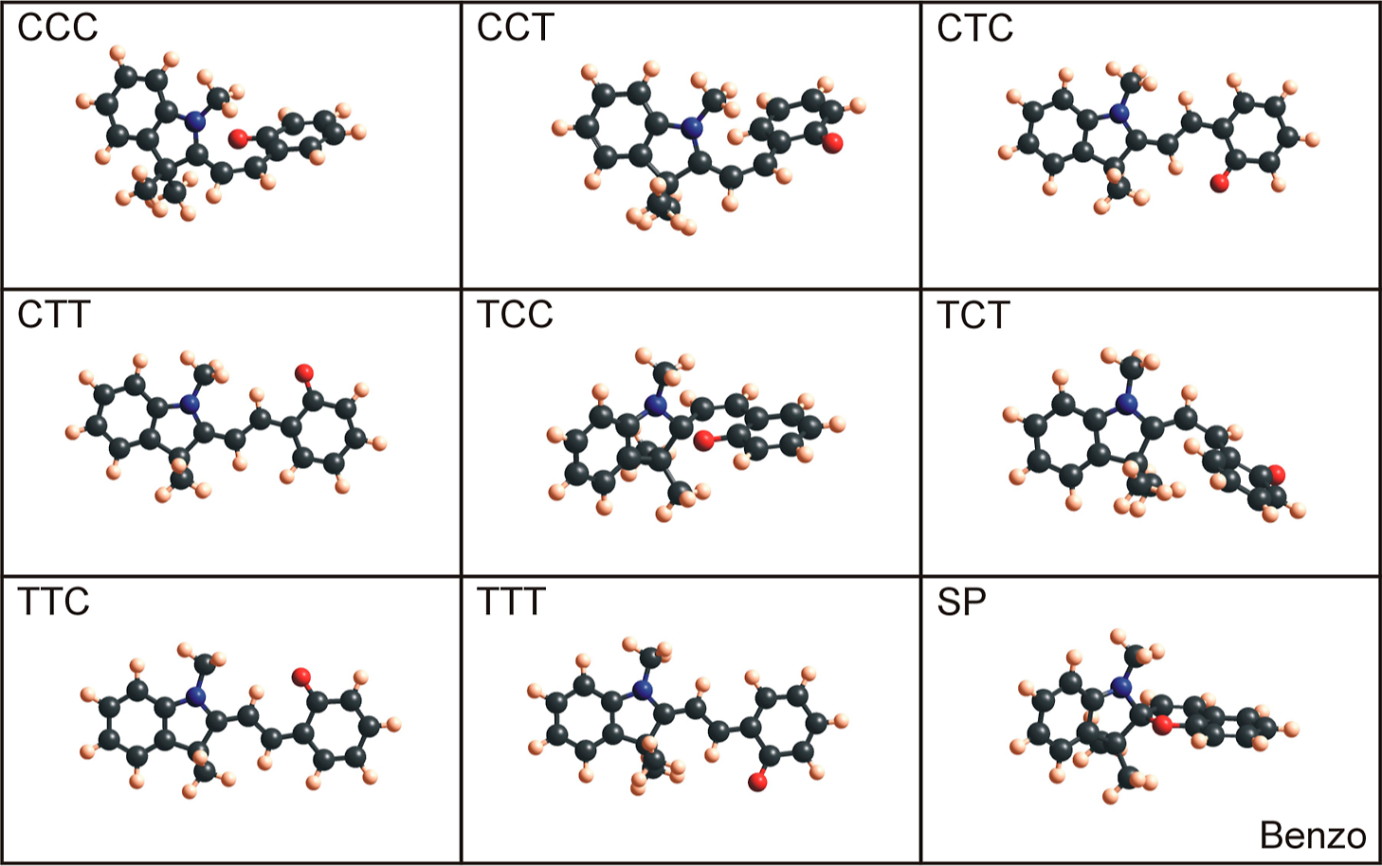
Optimized benzo merocyanine conformers and benzo spiropyran molecules using Gaussian with DFT-B3LYP and 6-31g basis sets.

After geometry-optimizing all the eight possible conformers of merocyanine and the spiropyran molecules in Gaussian with DFT-B3LYP and 6-31g basis sets, the configurations seen in Figures 4 (benzo), 5 (nitro), 6 (methoxy), and 7 (naphtho) are the results. Comparing all the eight conformers of MC, one observes that the four conformers with the central C–C bond in the *trans* orientation, T-conformers, are elongated and fairly planar. The C-conformers, on the other hand, are configured in such a way that the aromatic rings are angled with respect to each other. The main reason for this configuration are the methyl groups on the indoline unit, which make it energetically and geometrically unfavorable to have the aromatic ring with the attached oxygen in the same plane as the other aromatic rings. Especially interesting are the CCC and TCC conformers. When geometry-optimized with DFT-B3LYP, these configurations are (very) similar to the spiropyran isomer, where the indoline unit is perpendicular to the other aromatic ring (compare CCC and SP for naphtho and methoxy and TCC and SP for benzo and nitro). The six atoms, C-8, C-13, C-14, C-15, C-20, and O-21, are oriented in a hexagonal ring structure, although the central C–O bond (between C-8 and O-21) is broken in merocyanine isomers. As will be shown below, some of these C-conformers show strong parameter-dependent behavior when used as an adsorbate on the substrate and will change their configuration substantially.

**Figure 5:**
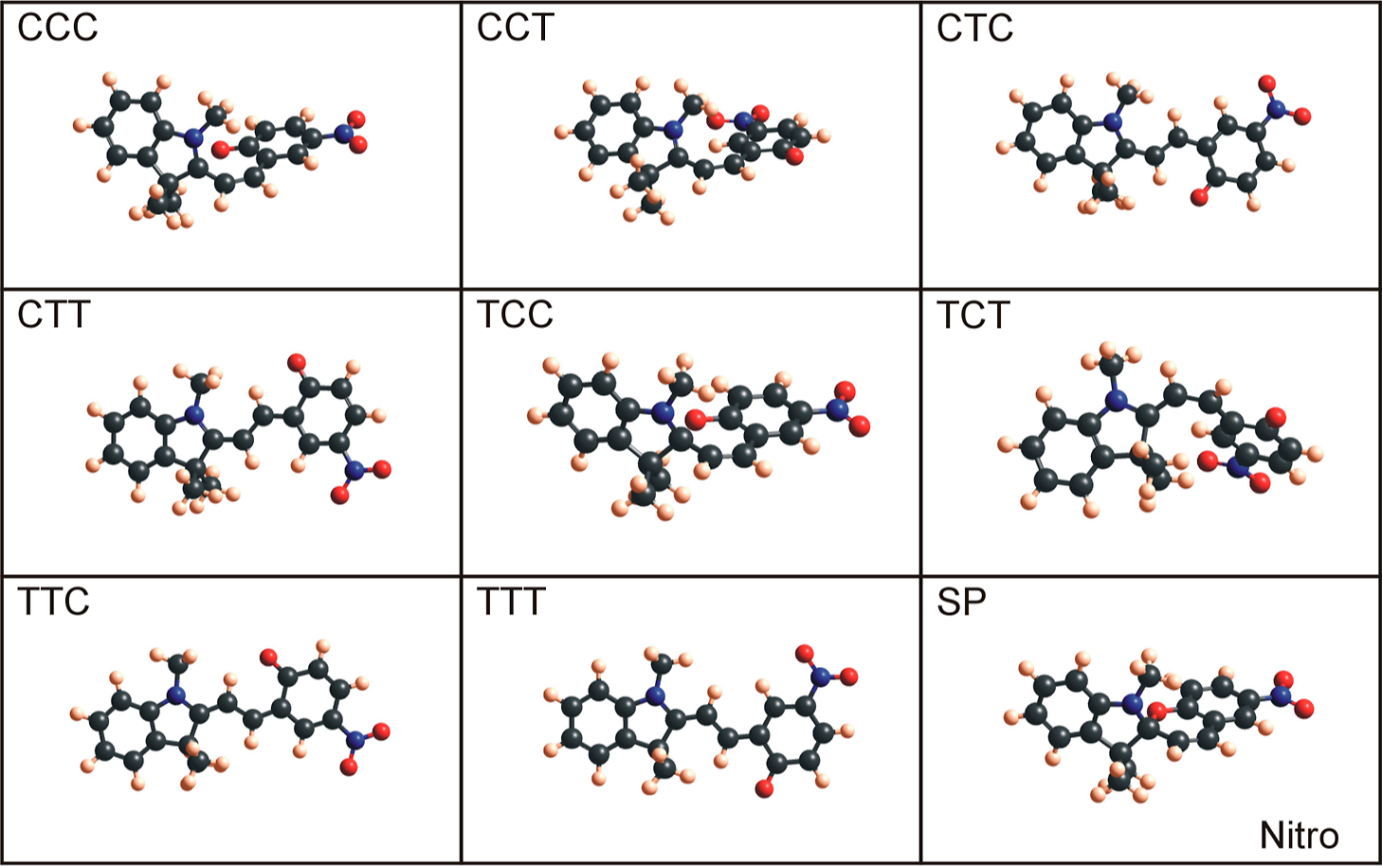
Optimized nitro merocyanine conformers and nitro spiropyran molecules using Gaussian with DFT-B3LYP and 6-31g basis sets.

**Figure 6:**
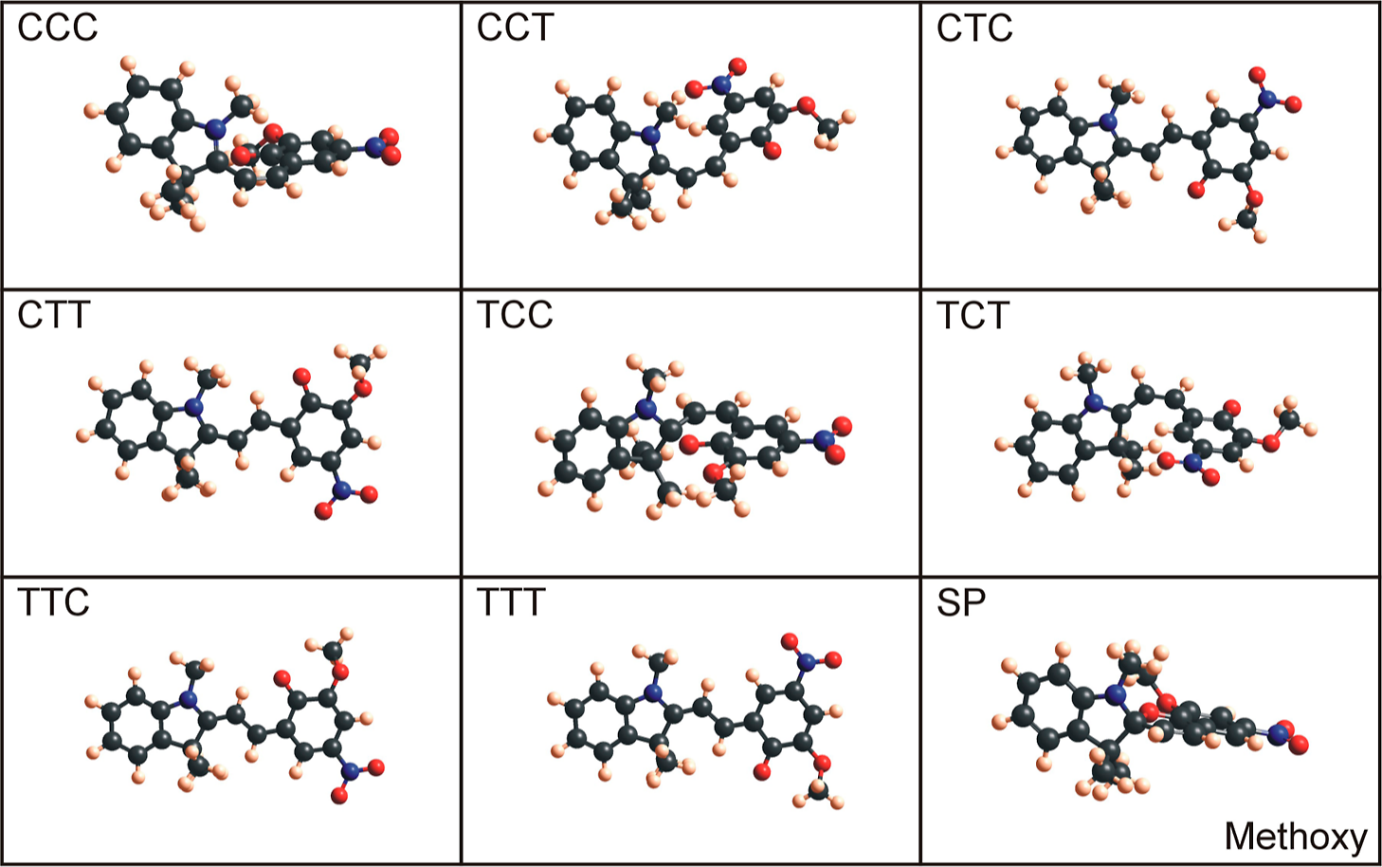
Optimized methoxy merocyanine conformers and methoxy spiropyran molecules using Gaussian with DFT-B3LYP and 6-31g basis sets.

**Figure 7:**
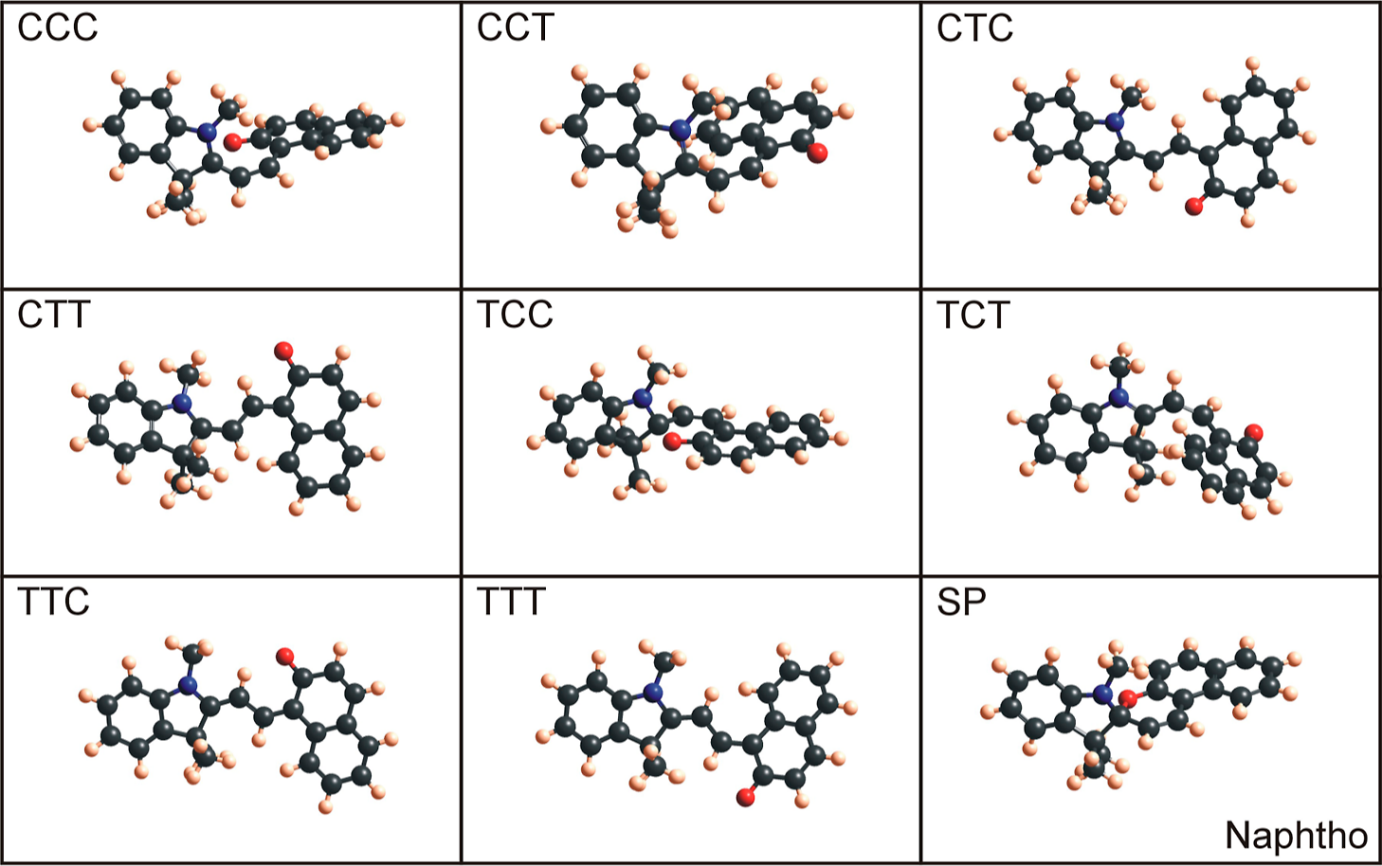
Optimized naphtho merocyanine conformers and naphtho spiropyran molecules using Gaussian with DFT-B3LYP and 6-31g basis sets.

**Figure 8:**
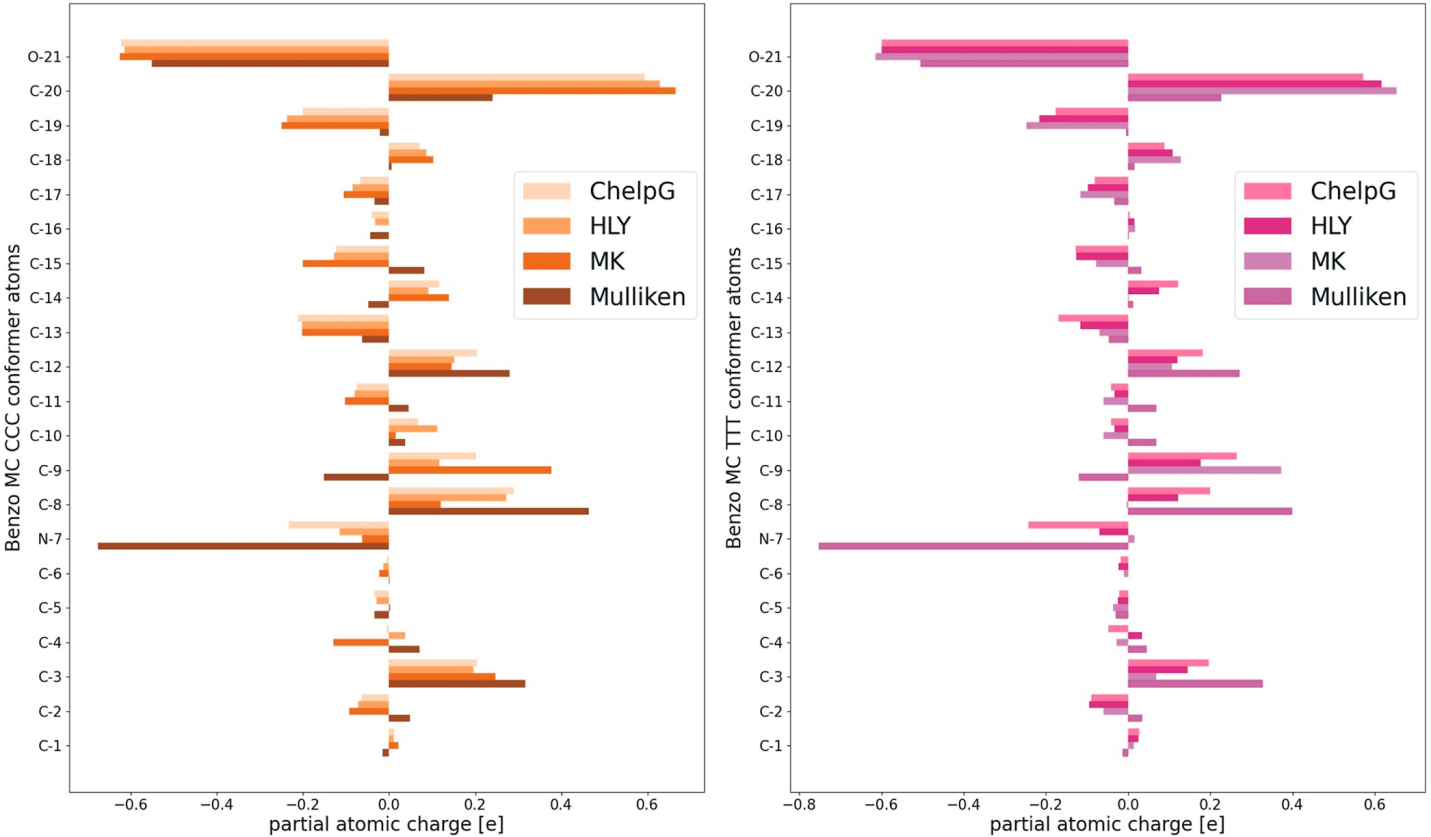
Partial atomic charges for Benzo CCC (left) and TTT (right) conformers, labeled by atom numbers, see Figure 2. Results depicted are for the following charge schemes: ESP methods are ChelpG, HLY, and M-K, as well as Mulliken population analysis.

In the next step, we analyzed the charges calculated by using various established charge schemes implemented in the Gaussian DFT software package. Since the individual partial atom charges are not an observable quantity, the “correct” scheme cannot be identified.^45^ We employed four of the more common schemes available, namely, Mulliken population analysis, ChelpG, Merz–Kollman (MK), and Hu-Lu-Yang (HLY).^46−51^ The complete and detailed results for the charge calculations for all molecules and conformers/isomers can be found in the Supporting Information (Figures S1–S8) with the labeling of atoms according to Figures 1 and 2. A representative example of these charge calculations can be seen in Figure 8, where we chose the Benzo-MC molecule as a CCC and TTT conformer, respectively. For most atoms, all the four charge schemes produce very consistent partial atomic charges. However, atoms N-7, C-9, and C-20 show consistently the highest deviations among the different charge schemes. One possible explanation is that these atoms are connected to end groups (N-7 to a CH_3_ end group, C-9 to two CH_3_ end groups, and C-20 to the oxygen atom), and therefore, the different charge schemes with their different choices for the grid of modeling the ESP are more prone to deviations for these atom groups. Another point of interest is the sign of the charges of some specific atoms. Often one can find a Kekulé structure of merocyanine with zwitterionic labeling of the oxygen as O^–^ and the nitrogen as N^+^. For all the eight conformers of the four merocyanine molecules, the oxygen’s charge is indeed calculated to be negative, with an average partial charge of about −0.4*e*. The nitrogen atom, though, has in most calculations also a negative partial charge, whereas the attached neighboring carbon atom, C-12 (including the attached H atoms), is calculated to indeed carry a positive partial charge. These findings therefore preserve the experimental findings of zwitterionic merocyanine molecules, as generally depicted. These overarching results for all conformers and molecules with an average charge of −0.4*e* on the oxygen (O-21) and +0.2*e* on the carbon attached to the nitrogen (C-12) create expected dipole moments for these molecules; an overview of dipole moments can be seen in Figure 10. Clearly, for all molecules, for the spiropyran isomer where the central C–O bond is existent and therefore the molecule is much more compact, the dipole moment is smallest compared to the merocyanine isomers, especially the planar T-conformers. Many of these T-conformers exhibit the highest dipole moments for the various isomers. The values obtained while using the Mulliken charge schemes are consistent with other reported results for spiropyran (around 2–6 debye) and merocyanine (for T-conformers, around 5–13 debye) with a factor of 2–3 higher values for merocyanine, which indicates agreement of our approach with previously reported data.^52,53^

**Figure 9:**
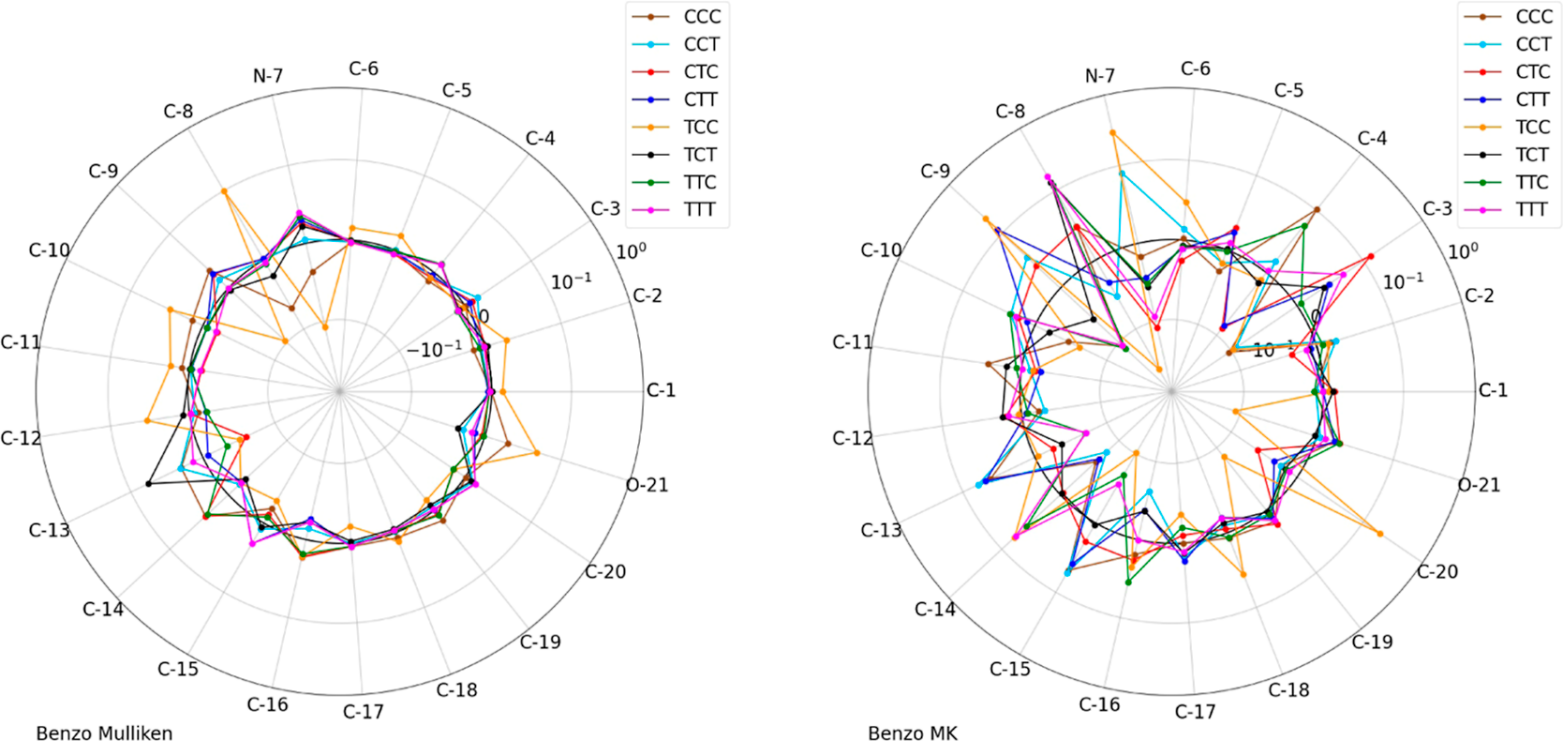
Comparison of charges for benzo merocyanine conformers by two charge schemes, Mulliken (left) and MK (right). The radial plots show the deviation from the mean for the partial charges on each atom. Using the Mulliken charge scheme (left) indicates that almost all atoms are assigned roughly similar charges independent of the conformer. On the other hand, looking at the charts for the MK scheme (right), one can clearly see a dependence of the assigned charges on the conformer configuration since the deviation from the mean is much more pronounced. The radial values (in symmetric logarithmic scale, measured in units of elementary charge *e*) are calculated as the actual assigned charge minus the average charge taken for a specific atom for all conformers. A value of nearly zero indicates that the assigned charge for that atom and conformer is the same as the average for all conformers.

**Figure 10:**
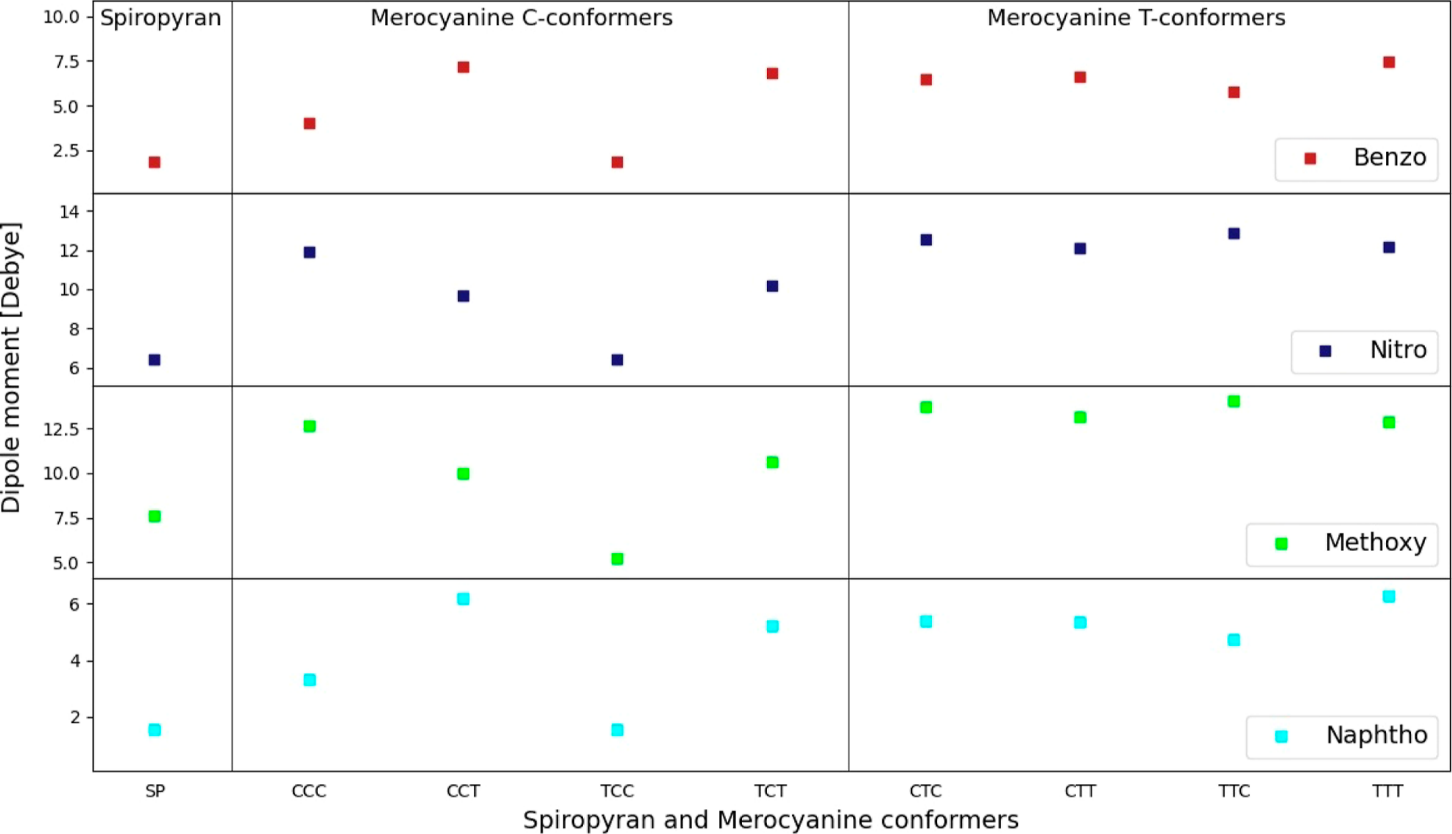
Overview of the dipole moments of the four different molecules and their respective spiropyran isomers and merocyanine conformers using the calculated charges by Mulliken population analysis. The spiropyran molecules (left side) have the lowest dipole moments, and in general, T-conformers (CTC, CTT, TTC, and TTT) have larger dipole moments. The TCC conformer has a dipole moment very similar to that of the spiropyran isomer because the configuration of the atoms is almost identical to that of the SP molecule; only the central C–O bond is broken in the merocyanine conformer.

Other observations are that the ESP charge schemes, not surprisingly, are for most cases more consistent with each other than with the Mulliken charges. However, overall, the charges on all the eight conformers calculated with the four schemes are fairly similar, as can be seen in the extensive data presentation in Figures S1, S3, S5, and S7.

Another outcome of the charge calculations for the different charge schemes is their sensitivity to various conformers. We found that the three ESP methods produce results for partial atomic charges that differ by conformer, sometimes drastically, due to the sensitivity of the ESP to geometric factors of the molecule. On the other hand, when using Mulliken population analysis, the partial atomic charges for different conformers are not very distinct. The complete results can again be found in the supplementary section (Figures S2, S4, S6, and S8); two examples for the benzo molecules can be seen in Figure 9. The data for this graph have been obtained by using the calculated charges for all the eight conformers at each respective atom and averaging the values for a specific atom over all conformers. Then, for each conformer, the actual charge on the atom was subtracted from the average, and these values are depicted in these radial plots. If the conformers’ geometries had no influence on the partial atomic charges, the values for the charge on a specific atom would not vary with conformers, and the difference between the charges for each conformer with the average would be nearly zero, leading to fairly circular plots. However, if the geometry does influence the partial charges, different conformers have different partial charges on identical atoms, and their difference with the average would be for some atoms greater than zero and for some atoms smaller than zero. That would create a noncircular plot, e.g., more zigzag structures appear. Two examples for each situation can be seen in Figure 9. In these graphs, fairly circular plots, which means similar charge values for all atoms and all conformers, indicate low dependency of charge assignment on the conformer, whereas a ragged graph indicates that the partial atomic charges are differing for a specific conformer. Looking at the left radial plot of Figure 9 using Mulliken population analysis, only small deviations for individual atoms can be found with respect to the different conformers. However, looking at the right graph using the Merz–Kollman ESP method, many atoms in the MC molecule have substantial differences from the median charge. There are various atoms which deviate by more than 0.1*e* from the average charge depending on the conformer, most notably the N-7 atom which for the TCC conformer had a more than 0.1*e* higher charge than the average, and the CTC conformer has a more than 0.1*e* lower charge than average. This clearly indicates the sensitivity of the ESP method to the geometric shapes of various conformers. These findings solidify that the use of ESP charges will be more applicable when looking at the adsorption of individual conformers.

In general, by determining the partial charges necessary for MM calculations, ESP charge methods are a suitable choice showing previously established trends of a zwitterionic merocyanine configuration with expected dipole moments and sensitivity toward conformer geometries for merocyanine.

### Adsorption Geometry and Energies

**Figure 11:**
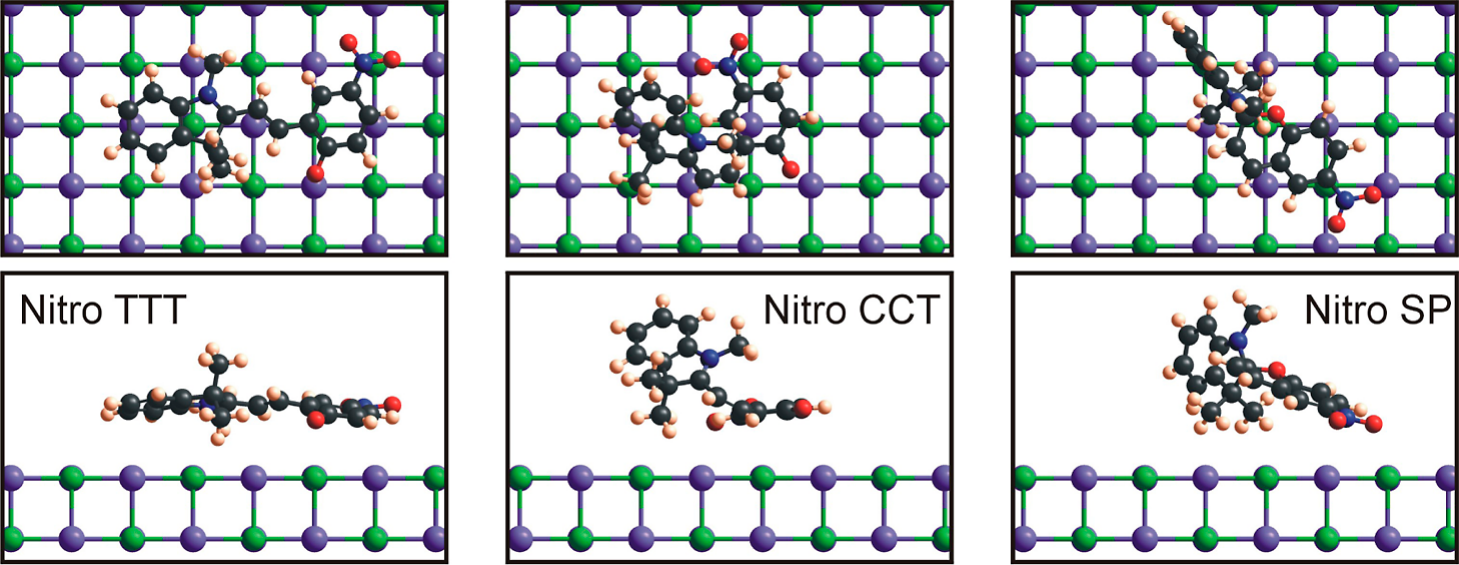
Adsorption geometry with top view and side view (as an example) for Nitro TTT and CCT conformers and Nitro SP molecule. In this case, the ChelpG charge scheme, AMBER 3 force field, and NaCl067 were used. One can clearly see the nearly parallel, flat adsorption of the TTT conformer (left), whereas the CCT conformer adsorbs fairly unordered. The spiropyran molecule consists of two sets of perpendicular rings which cause the molecule to adsorb with one set nearly perpendicular to the substrate and the other two rings closer to the NaCl surface. The substrate is rendered where chlorine (−) is shown in green and sodium (+) is shown in purple.

**Figure 12:**
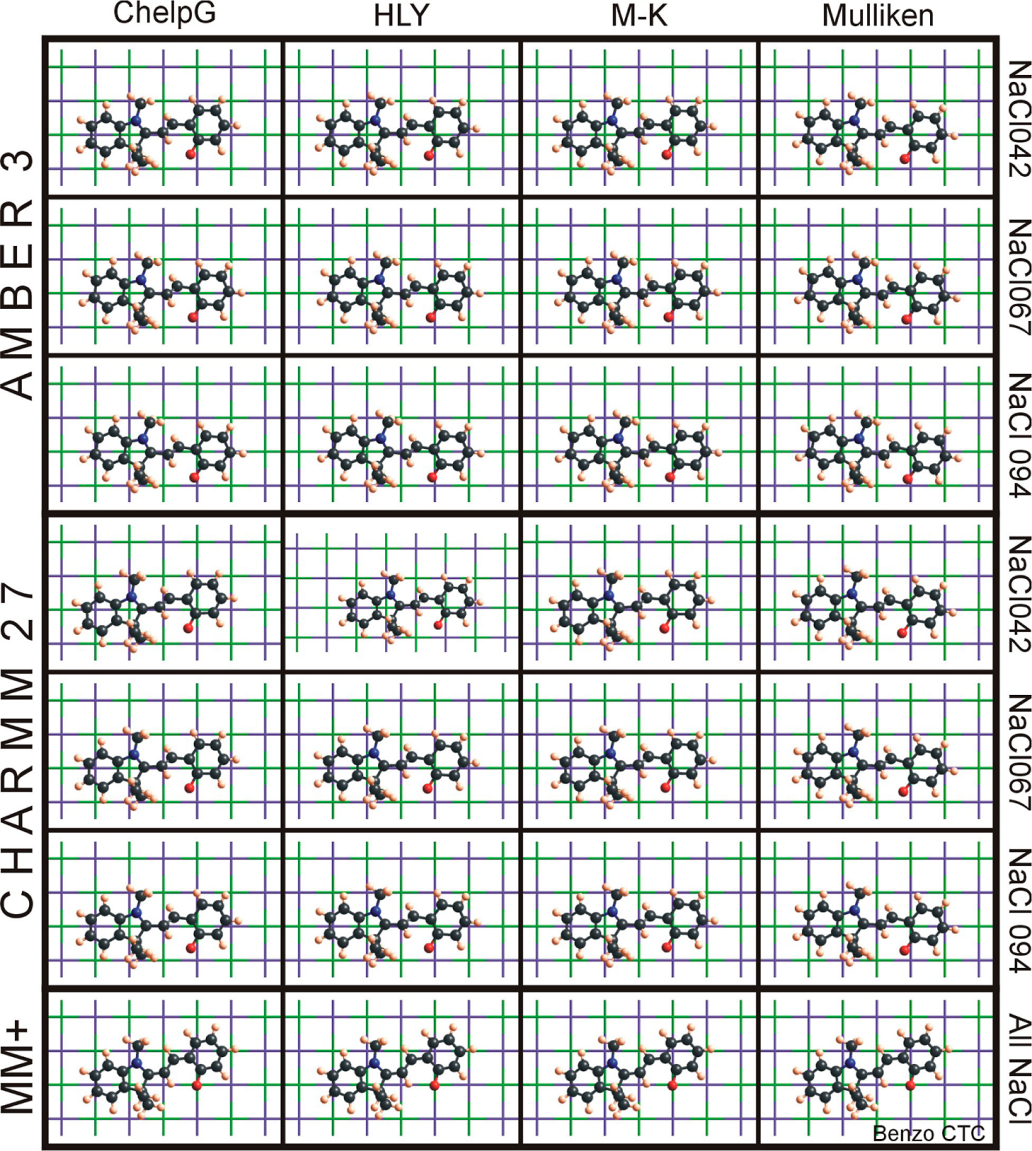
Adsorption geometry for Benzo CTC conformer using four charge methods (ChelpG, HLY, M-K, and Mulliken), three force fields, AMBER 3 (top three rows), CHARMM27 (rows 4–6), and MM+ (bottom row), and three substrate polarities (NaCl042—Na/Cl atoms with *q* = ±0.42*e*, NaCl067—Na/Cl atoms with *q* = ±0.67*e*, and NaCl094—Na/Cl atoms with *q* = ±0.94*e*). For force field MM+, there is no difference in geometry (and energy) when using different charge schemes for the molecule or a different polarity for the substrate. The substrate’s ionic bonds are rendered as tubes to show the grid: chlorine (−) is shown in green and sodium (+) is shown in purple.

Using three different MM methods (AMBER 3, CHARMM 27, and MM+) for each molecule with their eight MC conformers and the respective SP molecule, adsorption geometries and energies were determined. The complete set of data can be found in the Supporting Information. From the very large set of energy values and geometries, various common themes for all situations could be deduced, where one representative example is shown in more detail in Figure 11. The first overarching theme is regarding the geometry for the adsorption of merocyanine conformers and spiropyran molecules: T-conformers adsorb on the NaCl substrate in a mostly flat orientation with their aromatic rings parallel to the underlying substrate (see the left panel in Figure 11 for a nitro TTT conformer). These conformers only change their adsorption geometry slightly with respect to their original DFT-calculated free-space configuration. On the other hand, for C-conformers and spiropyran (middle and right panel depicting a nitro CCT conformer and the nitro spiropyran molecule), the adsorption geometry is such that the aromatic rings are only partly able to interact with the substrate, and therefore, more convoluted geometries occur. Generally, for almost all T-conformers, the overall orientation of the molecule is nearly independent of the force field and charge assignment of the molecule and substrate. A more comprehensive example for a benzo merocyanine conformer in the CTC orientation can be seen in Figure 12. In this graph (and the respective graphs in the Supporting Information), the columns are labeled according to the charge scheme which was used to assign partial charges to the molecule. The top three rows are the results when using the AMBER 3 force field with three different substrate polarizations, NaCl042, NaCl067, and NaCl094, corresponding to partial atomic charges of the substrate of ±0.42*e*, ±0.67*e*, and ±0.94*e*, respectively. Rows 4–6 show results using the CHARMM 27 force field, again with all the three substrate polarizations. The last row depicts the results when using MM+. Interestingly, when employing this force field, the geometries and energies for the adsorption are completely independent of the assigned charges for the molecule or the substrate. Using MM+, the only differences obtained was due to different conformers and therefore geometries of the molecule. In Figure 12, one can clearly see the consistent geometry of the adsorbate molecule as can be found with almost all T-conformers (CTC, CTT, TTC, and TTT) for all four molecules. The ionic bonds of the NaCl substrate are depicted as tubes (to focus attention on the adsorbate molecules’ geometry) with the intersections as the location of the substrate atoms (green is Cl^–^ and violet is Na^+^). Using Figure 12 as an example and looking a little more detailed at the adsorption geometries, one can find a not-too-surprising subtlety: the adsorption geometries of T-conformers show relatively little dependence on the force field and charge scheme. Looking closely in this figure, though, one can observe sometimes a slight rotation or a shift with respect to the underlying substrate, e.g., for AMBER 3 and CHARMM 27 (top six rows of the figure), the oxygen atom O-21 (as mentioned above given a consistently negative charge) is almost always very near (or above) a positive sodium substrate atom (violet cross). However, in some instances, when using MM+ (where the charges assigned do not show an influence on energy or geometry), a shift and slight rotation occurs to have this negatively charged oxygen atom above an also negative chlorine atom (green cross), see Figure 12, bottom row compared to the top eight rows. However, for C-conformers and SP, these findings are not observed. In general, it is difficult to make overarching statements about the adsorption of C-conformers and the spiropyran isomer. This indicates that the C-conformers exhibit only metastable behavior which is strongly influenced by the computational setup for the adsorption conditions and most likely will be harder to actually observe under experimental conditions.

**Figure 13:**
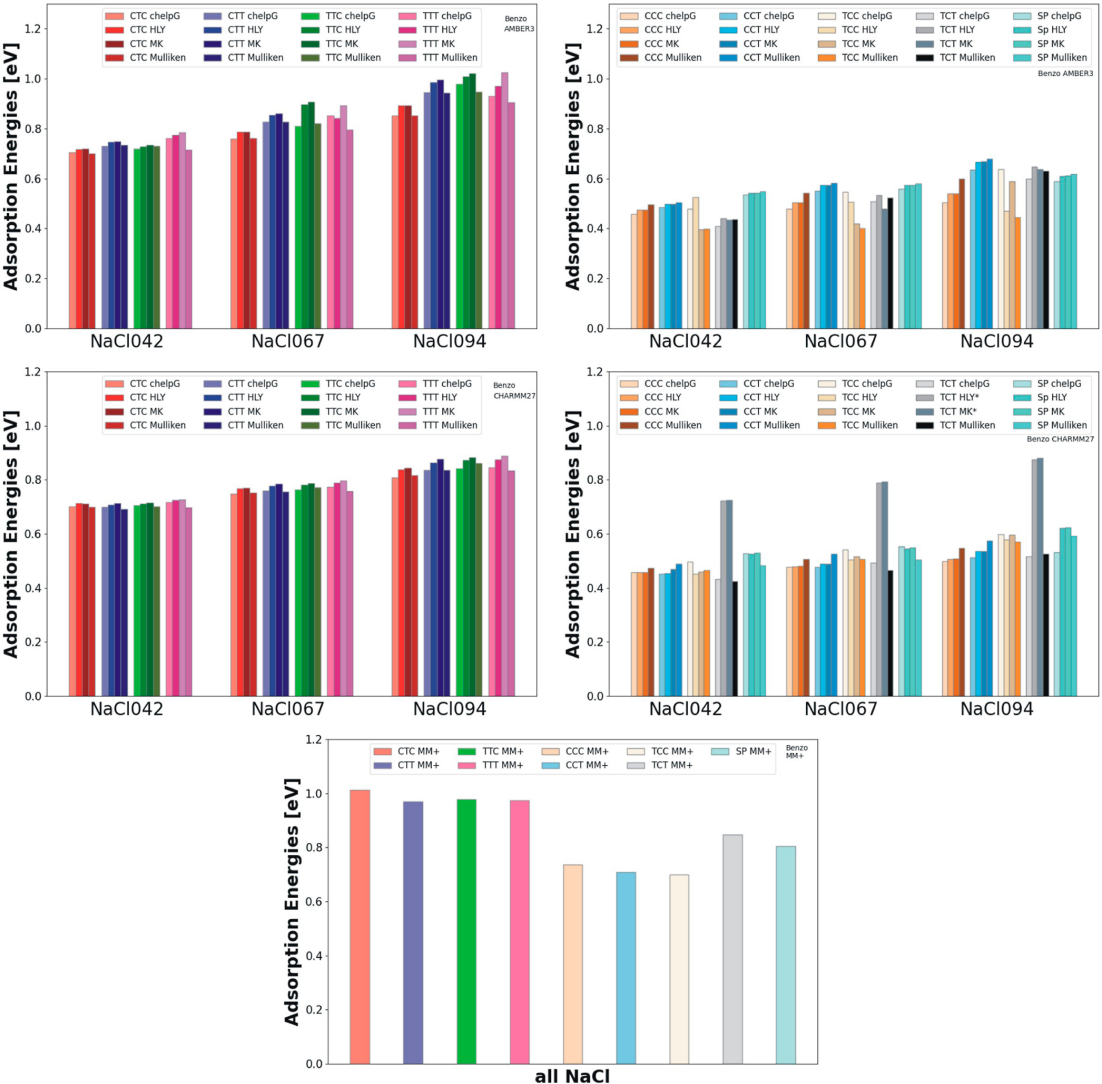
Comparison of adsorption energies for benzo merocyanine conformers and the spiropyran molecule using the AMBER 3 force field (top), CHARMM27 (middle), and MM+ (bottom). The T-conformers (left graphs) have overall a higher adsorption energy than C-conformers and spiropyran (right graphs) due to the parallel orientation of the aromatic ring of the T-conformers with the substrate. Also clearly visible are the increased energies with respect to substrate polarity with lowest energy on a NaCl substrate with ±0.42*e* as partial atomic charge on the sodium/chlorine atoms and highest adsorption energy for the substrate with ±0.94*e* as partial charge. Noticeable are also the nearly constant values for the energies when using the various charge schemes. For MM+ (bottom), all adsorption energies for a given conformer were independent of the charge assignments on the substrate and the molecule (*TCT conformer, using CHARMM27 and HLY/MK, switched to TTT conformer when adsorbed.).

The adsorption energies depicted as an example in Figure 13 for the Benzo-SP and MCs (for all molecules, see Supporting Information Figures S9–S12) also reveal an overarching scheme: First, T-conformers show higher adsorption energies than C-conformers and the spiropyran molecule when compared for the same substrate. Second, increasing the polarity of the substrate increases the adsorption energies but, as mentioned above, does not significantly influence the geometry for T-conformers. The adsorption energies for T-conformers for benzo molecules are increased from about 0.7 eV (for NaCl042) to about 0.9 eV (for NaCl094) using AMBER 3 and from 0.7 eV (for NaCl042) to 0.8 eV (for NaCl094) using CHARMM 27. Again, using MM+, the energies are independent of charge scheme and polarization of the substrate and were about 1.0 eV for T-conformers, which is about 0.2–0.3 eV higher than for C-conformers and for the SP molecules. For the other two force fields, AMBER 3 and CHARMM 27, the energies for the C-conformers and SP were also about 0.2–0.3 eV lower than those for T-conformers. This general trend with slightly different values was also observed for methoxy conformers (Figure S10), nitro conformers (Figure S11), and naphtho conformers (Figure S12).

To summarize the findings above, T-conformers have about 30% higher adsorption energies than C-conformers and spiropyran. Using MM, the interactions of the adsorbate with the substrate are mostly due to van der Waals forces. For T-conformers that adsorb in a flat orientation, all aromatic rings contribute to the energies, whereas for C-conformers and spiropyran, only one or two of the aromatic rings are oriented parallel to the surface. This discrepancy would account for the 30% difference in the observed adsorption energy. In a different study of larger molecules (PTCDA and CuPC) on NaCl, higher adsorption energies of around 1.7–2.3 eV were calculated, in line with the fact that for both of those molecules, several more aromatic rings are oriented parallel to the substrate and therefore contribute to the adsorption energies.^40^

The remarkable findings of this study are that although four different molecules with many different geometric and electronic configurations were used, we found that when looking at merocyanine/spiropyran isomers, the calculations for only T-conformers of the MC form will result in (nearly) identical configurations upon adsorption on a substrate, independent of the choice of charge scheme, substrate polarity, or force field. For the C-conformers, the calculations led to varied geometries and lower adsorption energies. The lower energies could indicate that these conformers could be more easily (partially) desorbed from the substrate and then reconfigure themselves into T-conformers with higher adsorption energy. We postulate that when the central C–O bond of the spiropyran molecule is broken, a T-conformer will eventually emerge, and long-term stable adsorption will happen as one of the four possible T-conformer.

### Isomerization of Adsorbed Molecules

The choice of adsorbate, spiropyran, and merocyanine isomers was motivated as use in molecular switches by breaking the central C–O bond of spiropyran and creating a merocyanine isomer. The behavior of this class of molecules is very interesting for a wide variety of applications such as reusable sensors, high-resolution imaging in biological samples, and detection and imaging of mechanical stress.^54^ The initiation of these processes for adsorbed molecules is hindered by various surface–molecule interactions as opposed to gas-phase switching. However, when looking purely from an adsorption standpoint, possible switching from spiropyran to merocyanine would require only a partial amount of the binding energy of SP to be provided since only two of the four aromatic rings are bond to the substrate and additionally to the energy to break the C–O bond. The reverse process, on the other hand, is harder to accomplish when T-conformers of merocyanine are adsorbed since a full desorption process (breaking the van der Waals bonds of all the three or four aromatic rings with the substrate) would be necessary before re-establishing the central C–O bond. This would require more energy than that in solution, and the yield would naturally be much lower. In general, though, these processes can be accomplished depending on the substrate configuration by optically illuminating the SP molecules to switch to MC molecules and reversing the process by thermal activation, which accomplishes the aforementioned need to overcome the adsorption energy barrier.^18,55−57^ The higher activation energy of the MC to SP reaction indicates that the MC configuration would be more stable, as shown for similar systems.^18,57^

## Conclusions

In this study, we have employed four different MC/SP molecules and used DFT and MM calculations to determine adsorption geometries and energies and provide a precursor for the surface isomerization process between merocyanine and spiropyran. Initially, using DFT methods to determine partial atomic charges on the various molecules, the assignment of charges of individual atoms can vary based on the specific methods employed, dependent on the charge schemes and conformer. However, when looking at adsorption energies and geometries and separating the conformer into T-conformers (where the central bond is in *trans* orientation) and C-conformers (where the central bond is in *cis* orientation) together with the SP isomer, we found two general themes: the T-conformer shows nearly identical geometries as stable conformations for the whole parameter space during the calculations; however, the adsorption geometries significantly differ for C-conformers where adsorption geometries are inconsistent when using different charge schemes, substrate polarities, or force fields. Adsorption energies of these T-conformers vary to some degree for different charge methods and force fields, but they increase consistently with rising polarity of the substrate. Overall, these adsorption energies are about 30% higher for T-conformers than for SP and C-conformers. These findings are observed for all four investigated MC/SP molecules. Geometric adsorption configuration and associated adsorption energies provide a blueprint for possible isomerization reactions of adsorbed SP molecules to T-conformers as results and the reverse reaction back to SP conformers, meaning that when adsorbed SP molecules are exposed to external stimuli such as UV light which enables the bond breaking of the central C–O, the subsequent formation of merocyanine T-conformers is energetically more favorable than the formation of merocyanine C-conformers. Therefore, the reverse reaction from merocyanine T-conformers to spiropyran by re-establishing the C–O bond by different external stimuli such as visible light or heat is hindered by a higher energy barrier.

## Supporting Information Available

Partial atomic charges for benzo, methoxy, naphtho, nitro merocyanines, and spiropyrans; adsorption energies for all molecules; and adsorption geometries for all molecules (PDF)
