# Supplementary material for: Atomic Charge Dependency of Spiropyran/Merocyanine Adsorption as a Precursor to Surface Isomerization Reactions
Source: ACS Omega. 2023 Dec 28;9(1):798–810. doi: 10.1021/acsomega.3c06712 (PMC10785610; doi:10.1021/acsomega.3c06712)
Supplement: Supplementary file 1 — ao3c06712_si_001.pdf [file ao3c06712_si_001.pdf]

Supporting Information Available

Atomic charge dependency of Spiropyran/Merocyanine adsorption as a precursor to surface isomerization reactions

Andreas Riemann, Lauren Rankin, and Dylan Henry

Department of Physics & Astronomy, Western Washington University, 516 High St, Bellingham, WA 98225, USA

E-mail: andreas.riemann@wwu.edu

Phone: 360.650.2856

In this section all the information regarding partial atomic charges and adsorption geometries and energies can be found. This consist of data for 4 molecules, 8 MC conformers and 1 SP isomer, 4 charge schemes, 3 polarities for the substrate investigated using 3 different force fields. The following tables provide an overview of the graphs:

Figure number	Content
Figure S1	partial atomic charges calculated using various charge schemes sorted by Benzo MC conformers & SP isomer
Figure S2	comparison of partial atomic charges for Benzo MC conformers & SP isomer by charge scheme
Figure S3	partial atomic charges calculated using various charge schemes sorted by Methoxy MC conformers & SP isomer
Figure S4	comparison of partial atomic charges for Methoxy MC conformers & SP isomer by charge scheme
Figure S5	partial atomic charges calculated using various charge schemes sorted by Nitro MC conformers & SP isomer
Figure S6	comparison of partial atomic charges for Nitro MC conformers & SP isomer by charge scheme
Figure S7	partial atomic charges calculated using various charge schemes sorted by Naphtho MC conformers & SP isomer
Figure S8	comparison of partial atomic charges for Naphtho MC conformers & SP isomer by charge scheme

Figures for adsorption energies for all force fields and four molecules

Figure number	Content
Figure S9	adsorption energies for **Benzo** molecules for all three force fields (AMBER 3, CHARMM27, MM+) and all charge configurations
Figure S10	adsorption energies for **Methoxy** molecules for all three force fields (AMBER 3, CHARMM27, MM+) and all charge configurations
Figure S11	adsorption energies for **Nitro** molecules for all three force fields (AMBER 3, CHARMM27, MM+) and all charge configurations
Figure S12	adsorption energies for **Naphtho** molecules for all three force fields (AMBER 3, CHARMM27, MM+) and all charge configurations

Overview of figures for adsorption geometries for Benzo MC conformers and Benzo SP isomer

Figure #	conformer	Figure #	conformer	Figure #	conformer/isomer
S13	MC CCC	S14	MC CCT	S15	MC CTC
S16	MC CTT	S17	MC TCC	S18	MC TCT
S19	MC TTC	S20	MC TTT	S21	SP

Overview of figures for adsorption geometries for Nitro MC conformers and Nitro SP isomer

Figure #	conformer	Figure #	conformer	Figure #	conformer/isomer
S22	MC CCC	S23	MC CCT	S24	MC CTC
S25	MC CTT	S26	MC TCC	S27	MC TCT
S28	MC TTC	S29	MC TTT	S30	SP

Overview of figures for adsorption geometries for Methoxy MC conformers and Methoxy SP isomer

Figure #	conformer	Figure #	conformer	Figure #	conformer/isomer
S31	MC CCC	S32	MC CCT	S33	MC CTC
S34	MC CTT	S35	MC TCC	S36	MC TCT
S37	MC TTC	S38	MC TTT	S39	SP

Overview of figures for adsorption geometries for Naphtho MC conformers and Naphtho SP isomer

Figure #	conformer	Figure #	conformer	Figure #	conformer/isomer
S40	MC CCC	S41	MC CCT	S42	MC CTC
S43	MC CTT	S44	MC TCC	S45	MC TCT
S46	MC TTC	S47	MC TTT	S48	SP

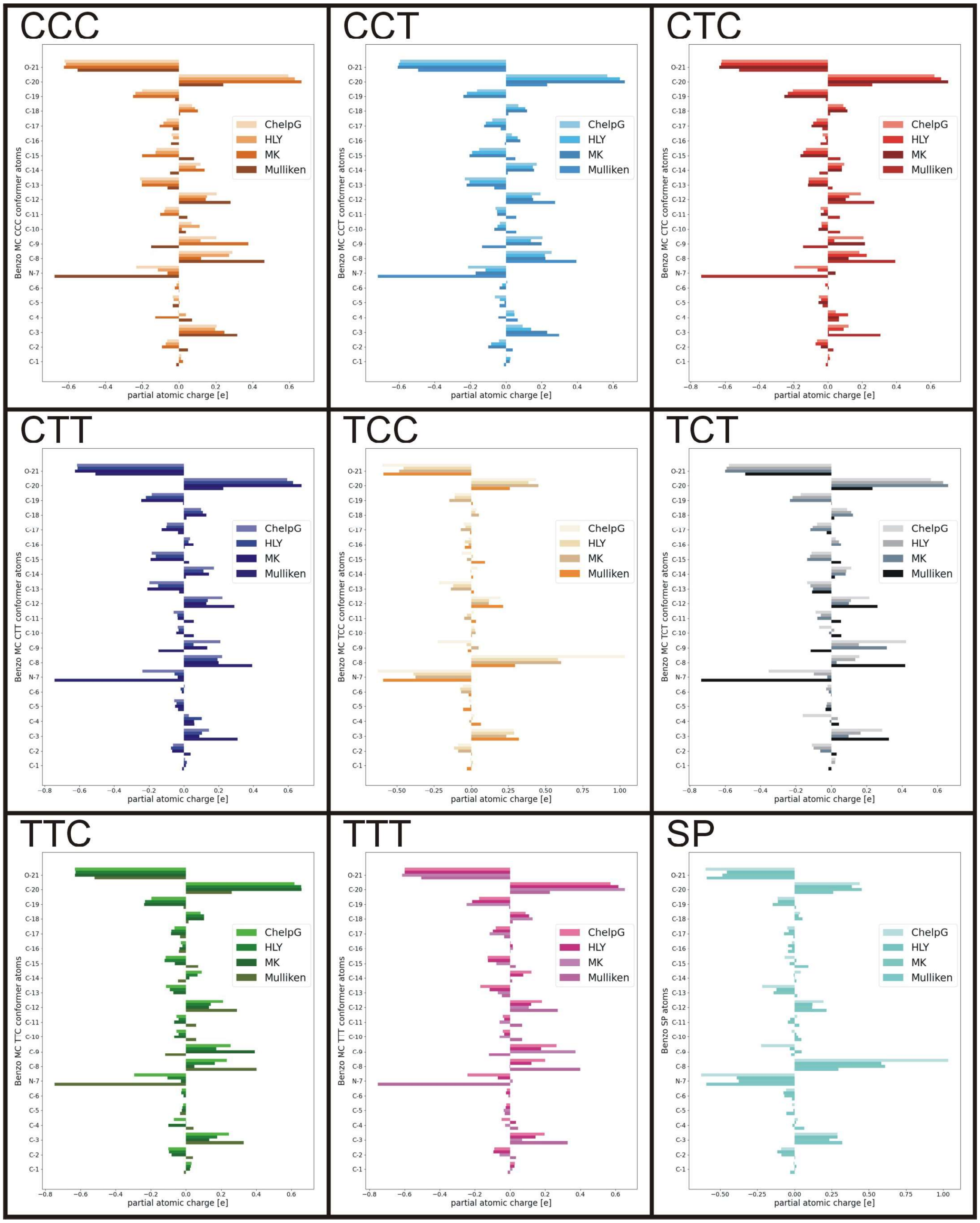

Figure S1: Partial atomic charges for all Benzo merocyanine conformers and Benzo spiropyran. For most conformers and their atoms the different charge schemes (Mulliken population analysis, HLY method, Merz-Kollman and ChelpG) produce similar results in terms of magnitude and sign of the partial charge. However, for some of the atoms (e.g. C-14 & C-15 which are part of the central bonds) there is significant discrepancy between Mulliken on one hand, and the ESP charge methods on the other hand.

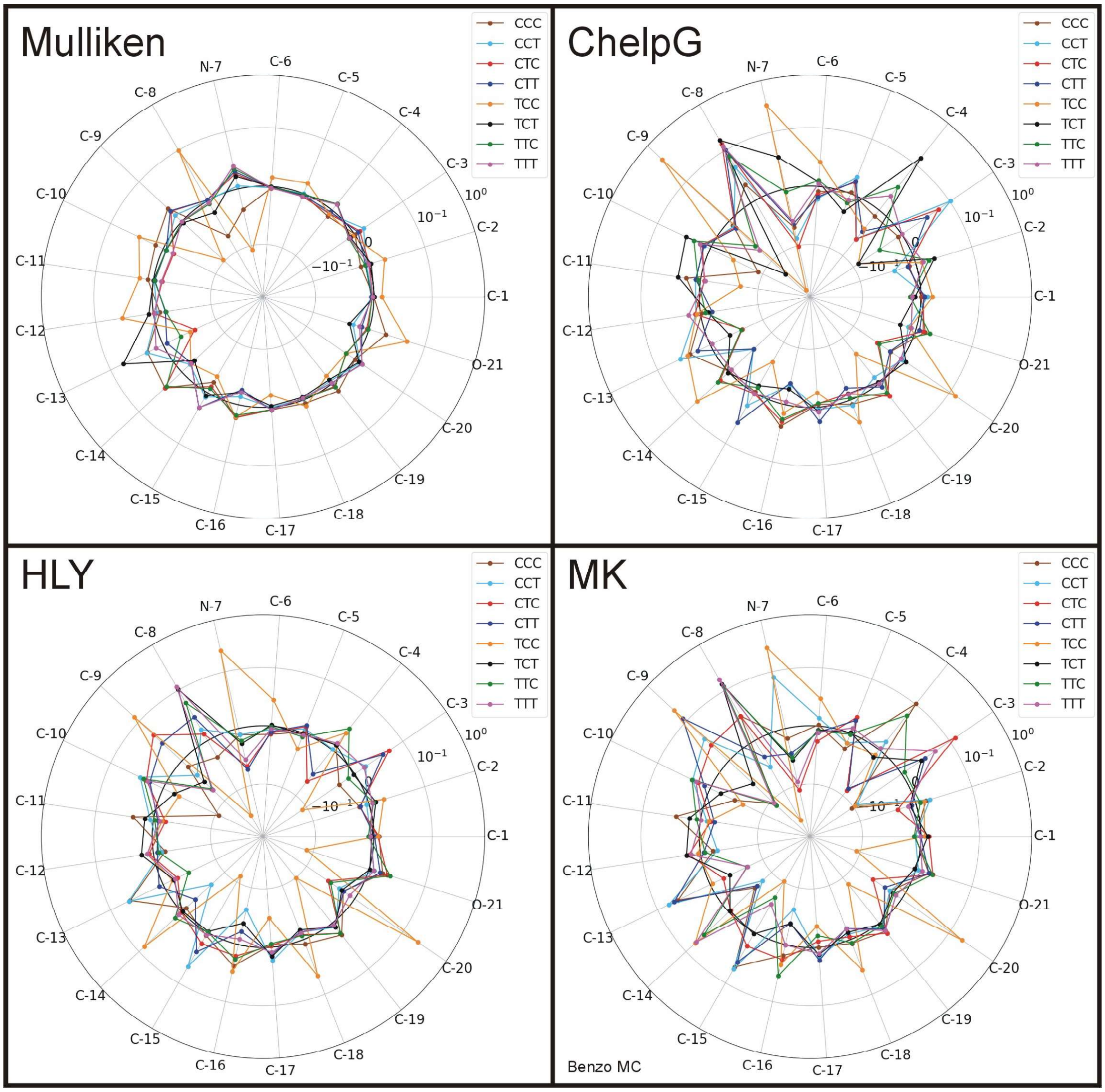

Figure S2: Comparison of charges for **Benzo merocyanine** conformers by charge scheme. The radial plots show the deviation from the mean for the partial charges on each atom. Using the Mulliken charge scheme (top left) indicates that almost all atoms are assigned a roughly similar charge independent of the conformer. On the other hand, looking at the charts for the other three methods, one can clearly see a dependence of the assigned charges on the conformer configuration since the deviation from the mean is much more pronounced. The radial values (in symmetric logarithmic scale, measured in units of elementary charge, e) are calculated as the actual assigned charge minus the average charge taken over all conformers. A value of zero indicates that the assigned charge for that atom and conformer is the same as the average for all conformers.

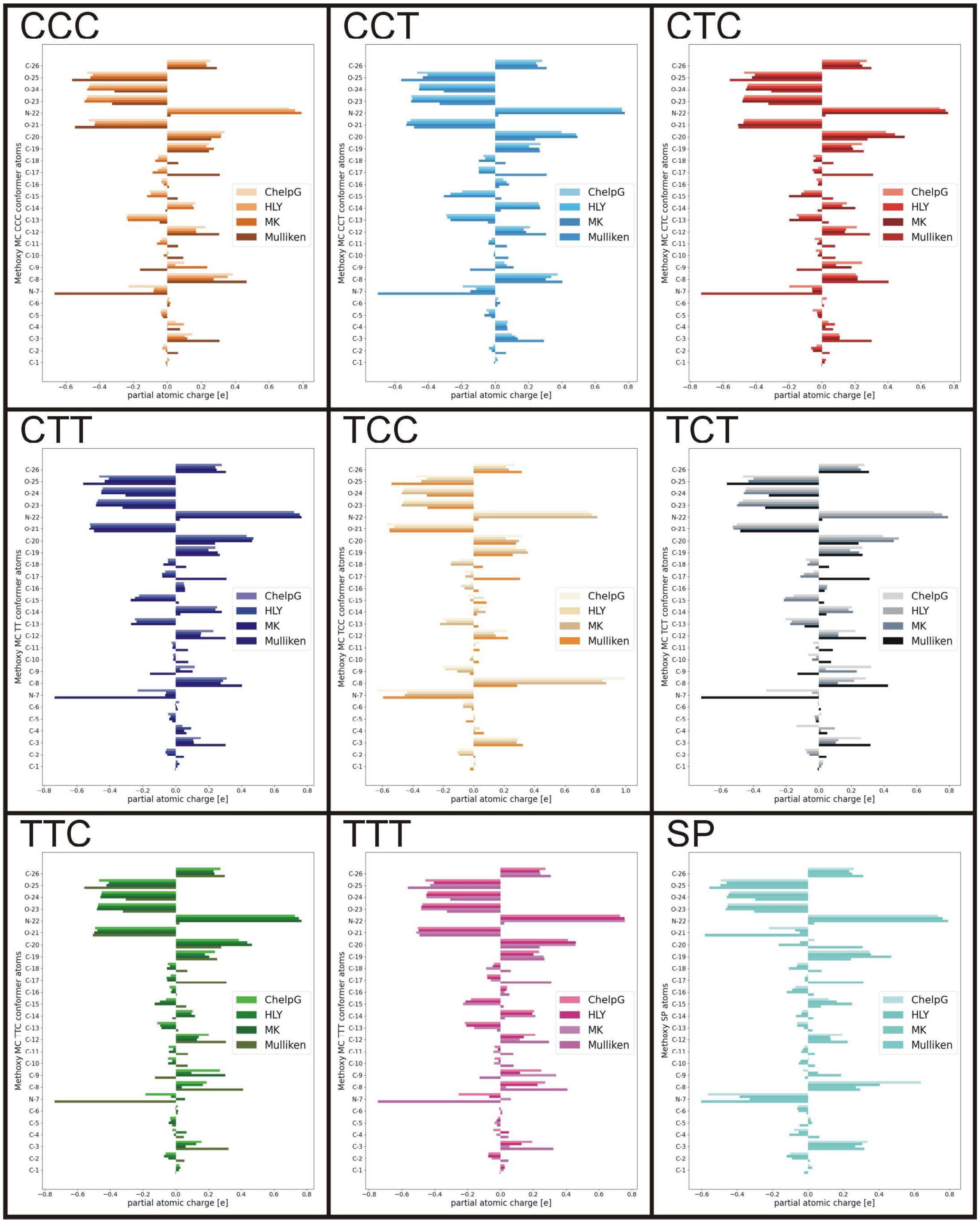

Figure S3: Partial atomic charges for all Methoxy merocyanine conformers and Methoxy spiropyran. For most conformers and their atoms the different charge schemes (Mulliken population analysis, HLY method, Merz-Kollman and ChelpG) produce similar results in terms of magnitude and sign of the partial charge. However, for some of the atoms (e.g. C-14 & C-15 which are part of the central bonds) there is significant discrepancy between Mulliken on one hand, and the ESP charge methods on the other hand.

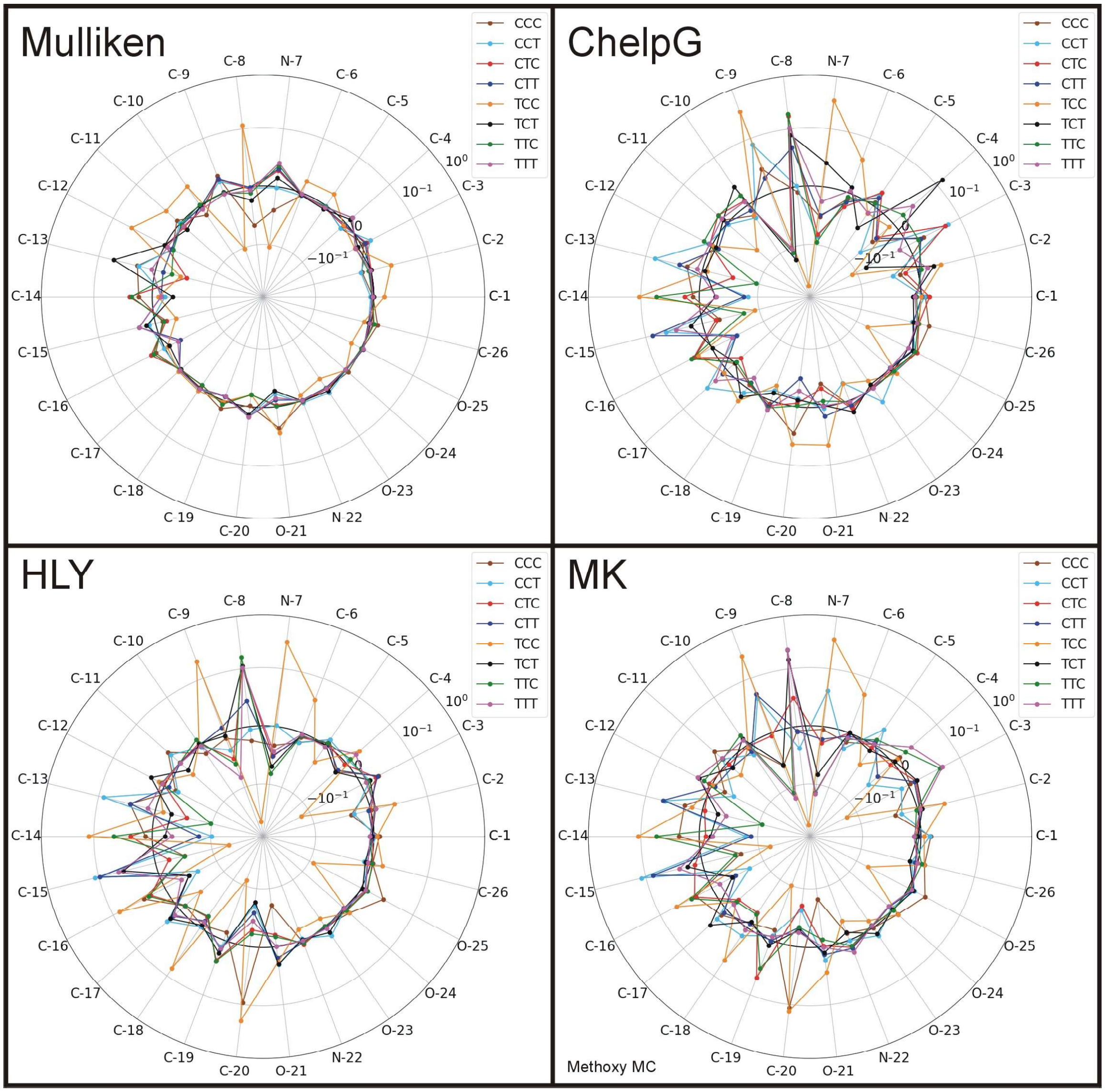

Figure S4: Comparison of charges for **Methoxy merocyanine** conformers by charge scheme. The radial plots show the deviation from the mean for the partial charges on each atom. Using the Mulliken charge scheme (top left) indicates that almost all atoms are assigned a roughly similar charge independent of the conformer. On the other hand, looking at the charts for the other three methods, one can clearly see a dependence of the assigned charges on the conformer configuration since the deviation from the mean is much more pronounced. The radial values (in symmetric logarithmic scale, measured in units of elementary charge, e) are calculated as the actual assigned charge minus the average charge taken over all conformers. A value of zero indicates that the assigned charge for that atom and conformer is the same as the average for all conformers.

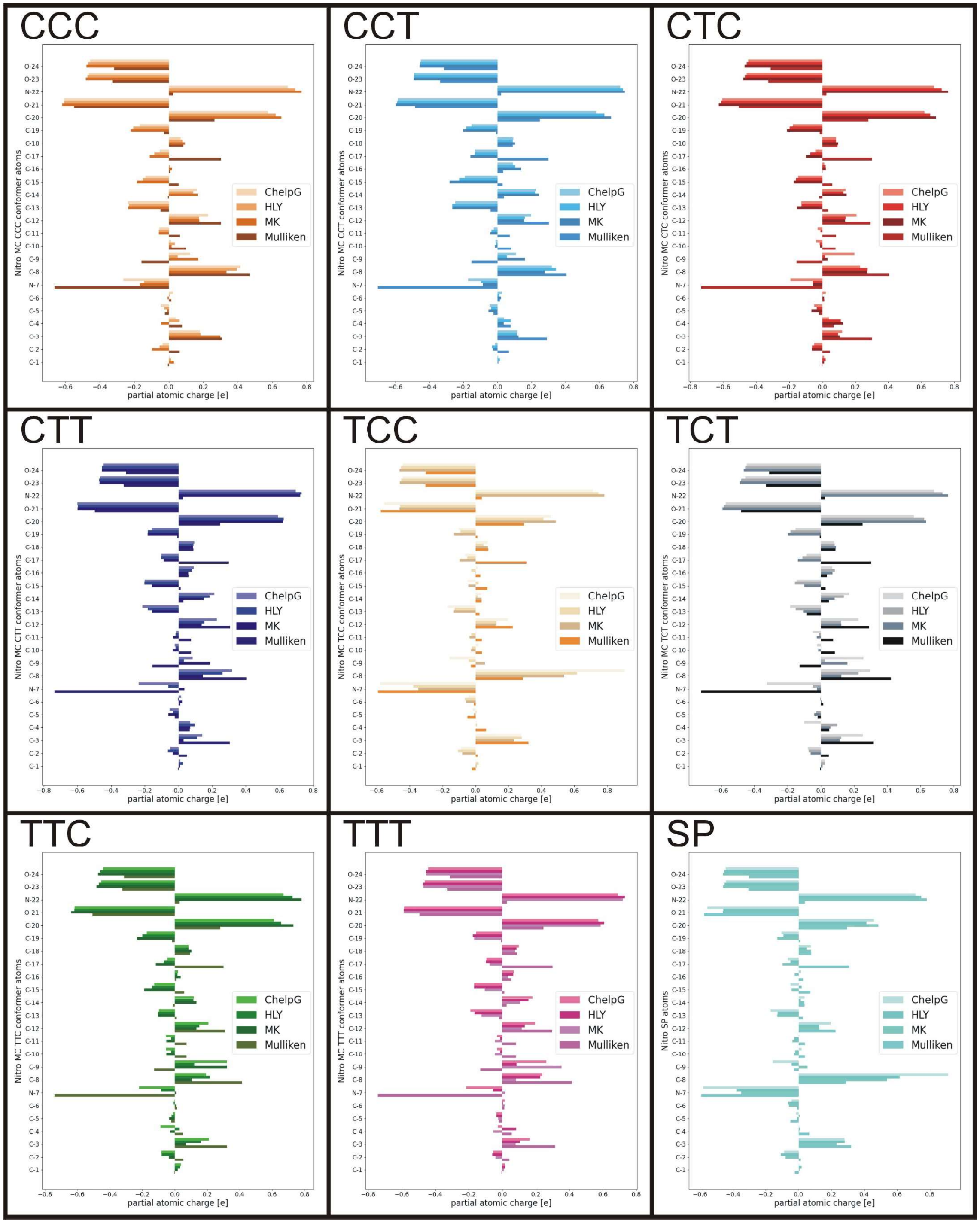

Figure S5: Partial atomic charges for all Nitro merocyanine conformers and Nitro spiropyran. For most conformers and their atoms the different charge schemes (Mulliken population analysis, HLY method, Merz-Kollman and ChelpG) produce similar results in terms of magnitude and sign of the partial charge. However, for some of the atoms (e.g. C-14 & C-15 which are part of the central bonds) there is significant discrepancy between Mulliken on one hand, and the ESP charge methods on the other hand.

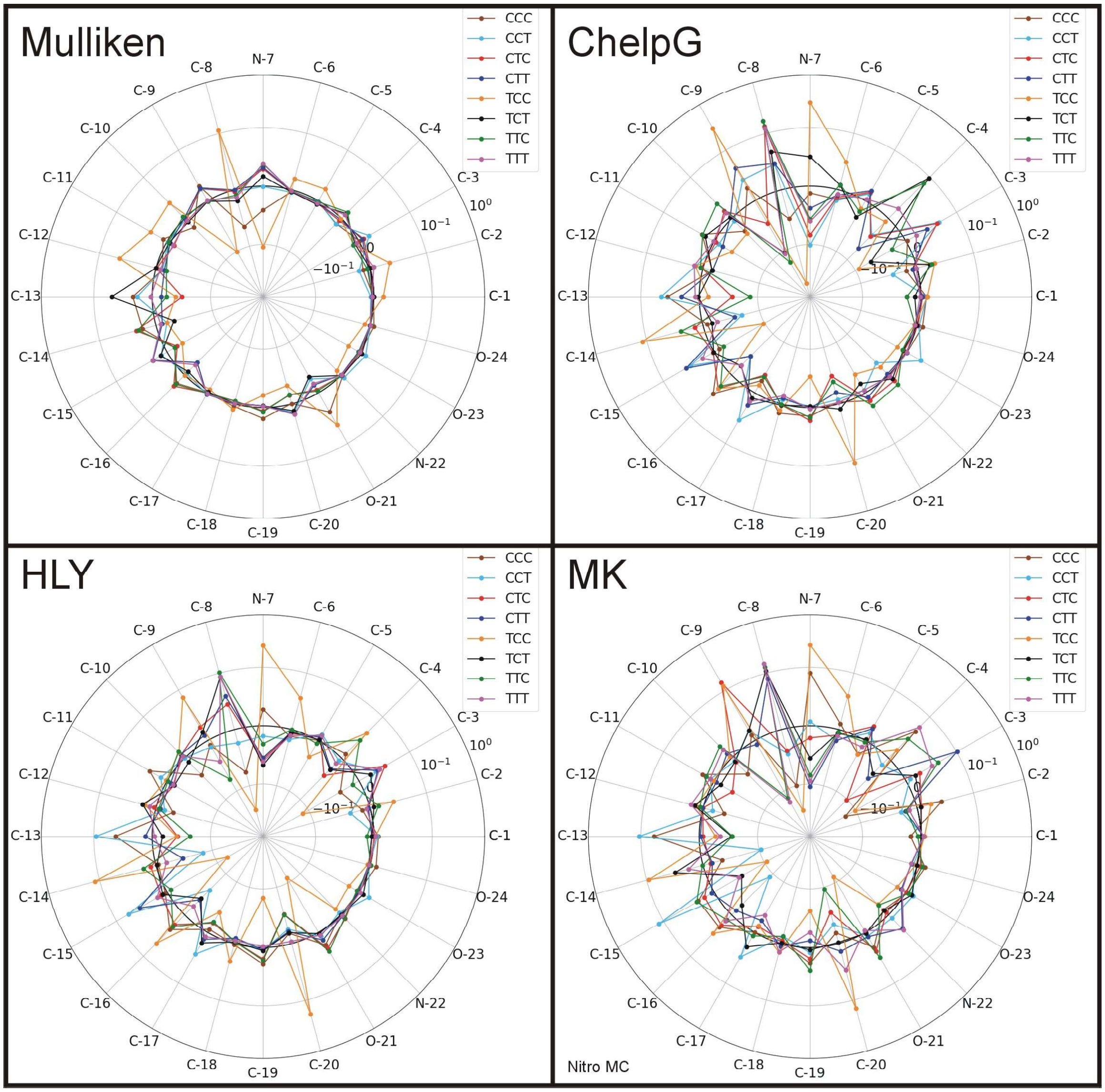

Figure S6: Comparison of charges for **Nitro merocyanine** conformers by charge scheme. The radial plots show the deviation from the mean for the partial charges on each atom. Using the Mulliken charge scheme (top left) indicates that almost all atoms are assigned a roughly similar charge independent of the conformer. On the other hand, looking at the charts for the other three methods, one can clearly see a dependence of the assigned charges on the conformer configuration since the deviation from the mean is much more pronounced. The radial values (in symmetric logarithmic scale, measured in units of elementary charge, e) are calculated as the actual assigned charge minus the average charge taken over all conformers. A value of zero indicates that the assigned charge for that atom and conformer is the same as the average for all conformers.

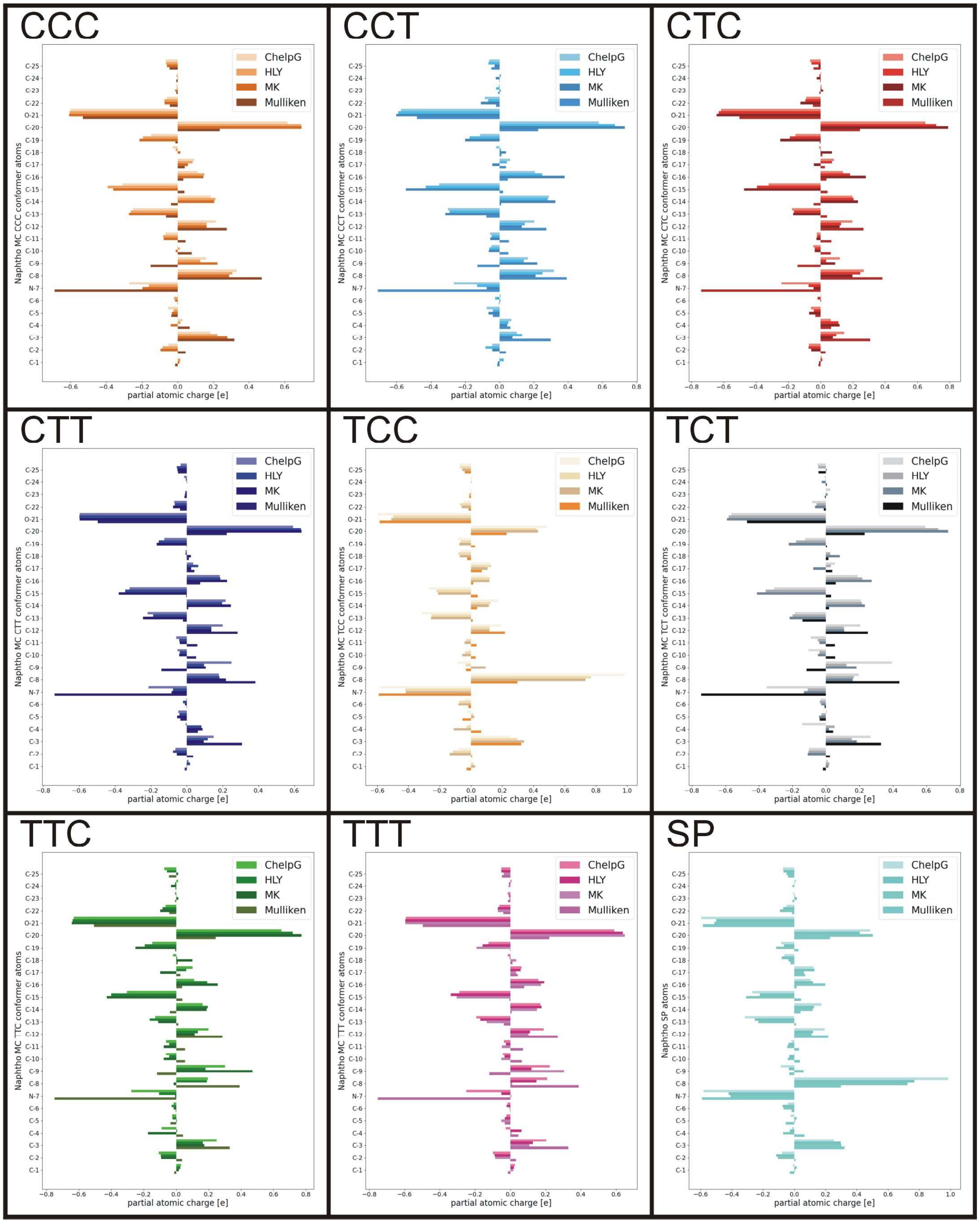

Figure S7: Partial atomic charges for all Naphtho merocyanine conformers and Naphtho spiropyran. For most conformers and their atoms the different charge schemes (Mulliken population analysis, HLY method, Merz-Kollman and ChelpG) produce similar results in terms of magnitude and sign of the partial charge. However, for some of the atoms (e.g. C-14 & C-15 which are part of the central bonds) there is significant discrepancy between Mulliken on one hand, and the ESP charge methods on the other hand.

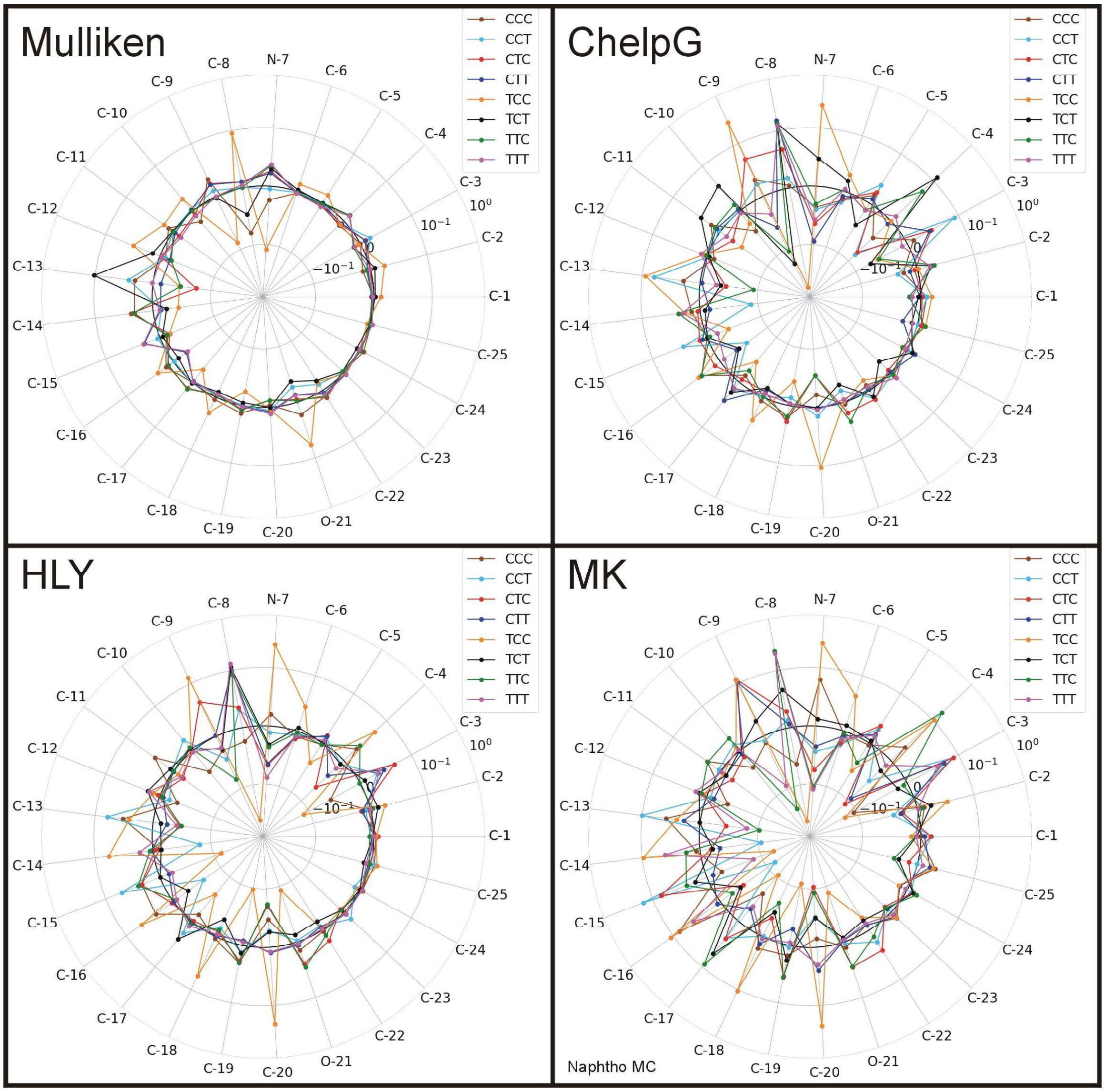

Figure S8: Comparison of charges for **Naphtho merocyanine** conformers by charge scheme. The radial plots show the deviation from the mean for the partial charges on each atom. Using the Mulliken charge scheme (top left) indicates that almost all atoms are assigned a roughly similar charge independent of the conformer. On the other hand, looking at the charts for the other three methods, one can clearly see a dependence of the assigned charges on the conformer configuration since the deviation from the mean is much more pronounced. The radial values (in symmetric logarithmic scale, measured in units of elementary charge, e) are calculated as the actual assigned charge minus the average charge taken over all conformers. A value of zero indicates that the assigned charge for that atom and conformer is the same as the average for all conformers.

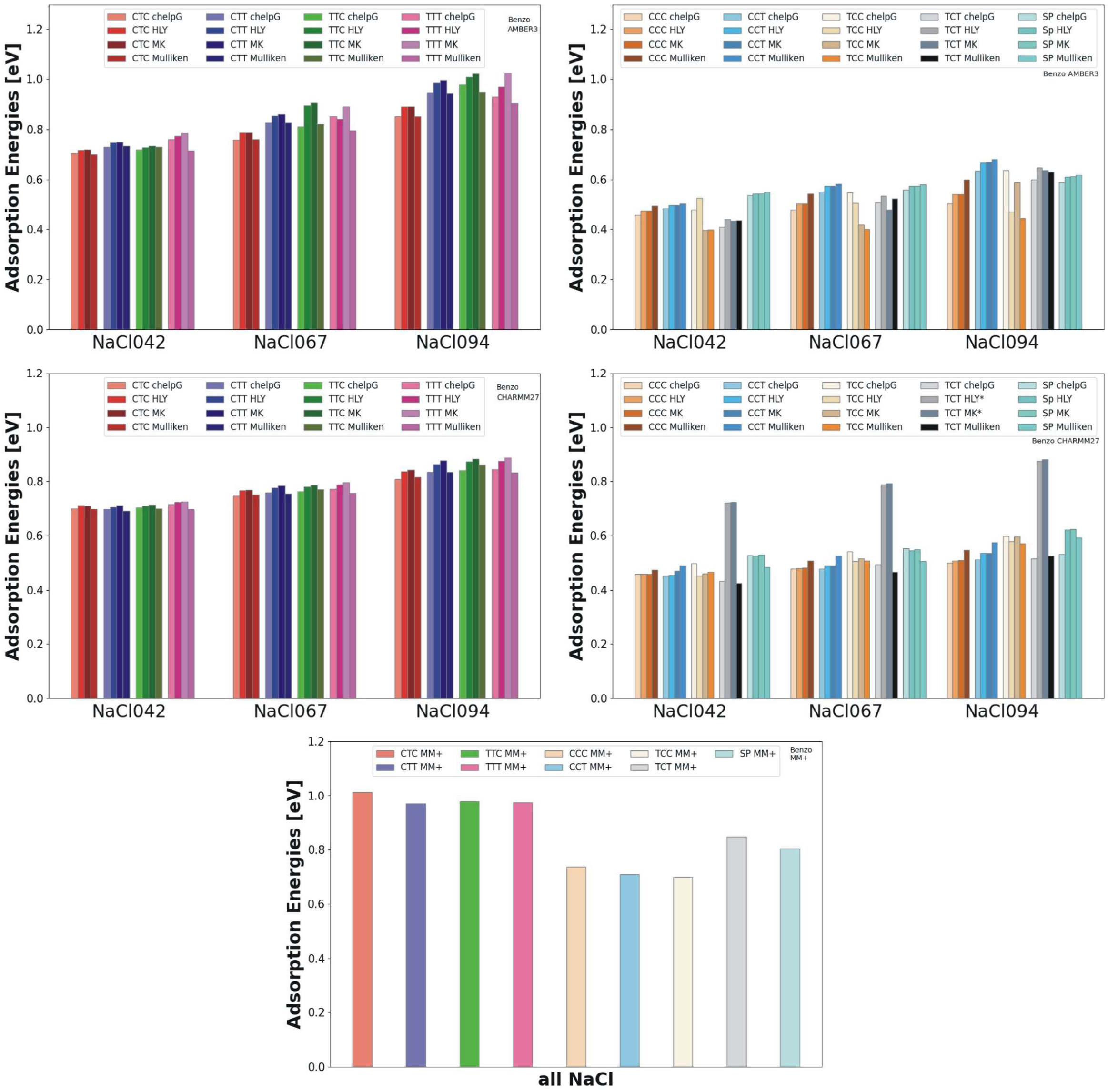

Figure S9: Comparison of adsorption energies for **Benzo** merocyanine conformers and spiropyran molecule using the **AMBER 3** force field (top), **CHARMM27** (middle) and **MM+** (bottom). The T-conformers (left graphs) have overall a higher adsorption energy than C-conformers and spiropyran (right graphs) due to the parallel orientation of the aromatic ring of the molecule with the substrate. Also clearly visible are the increased energies with respect to substrate polarity with lowest energy on a NaCl substrate with $\pm 0.42e$ as partial atomic charge on the sodium/chlorine atom, and highest adsorption energy for the substrate with $\pm 0.94e$ as partial charge. Noticeable are is also the nearly constant value for energies when using the various charge schemes. For MM+ (bottom), all adsorption energies for a given conformer were independent of the charge assignments on the substrate and the molecule. (*TCT conformer, using CHARMM27 and HLY/MK, switched to TTT conformer when adsorbed.)

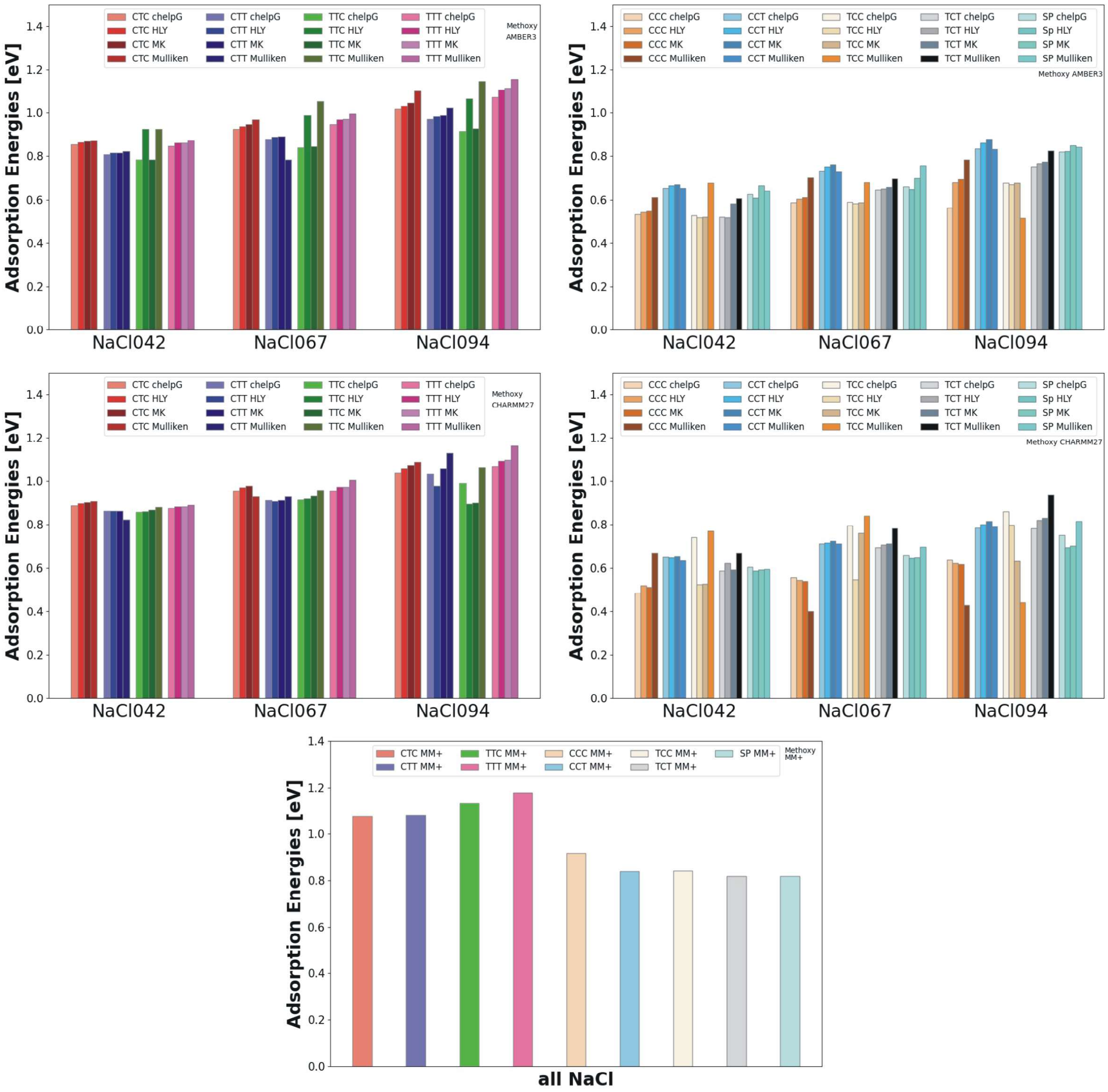

Figure S10: Comparison of adsorption energies for **Methoxy** merocyanine conformers and spiropyran molecule using the **AMBER 3** force field (top), **CHARMM27** (middle) and **MM+** (bottom). The T-conformers (left graphs) have overall a higher adsorption energy than C-conformers and spiropyran (right graphs) due to the parallel orientation of the aromatic ring of the molecule with the substrate. Also clearly visible are the increased energies with respect to substrate polarity with lowest energy on a NaCl substrate with $\pm 0.42e$ as partial atomic charge on the sodium/chlorine atom, and highest adsorption energy for the substrate with $\pm 0.94e$ as partial charge. Noticeable are is also the nearly constant value for energies when using the various charge schemes. For MM+ (bottom), all adsorption energies for a given conformer were independent of the charge assignments on the substrate and the molecule.

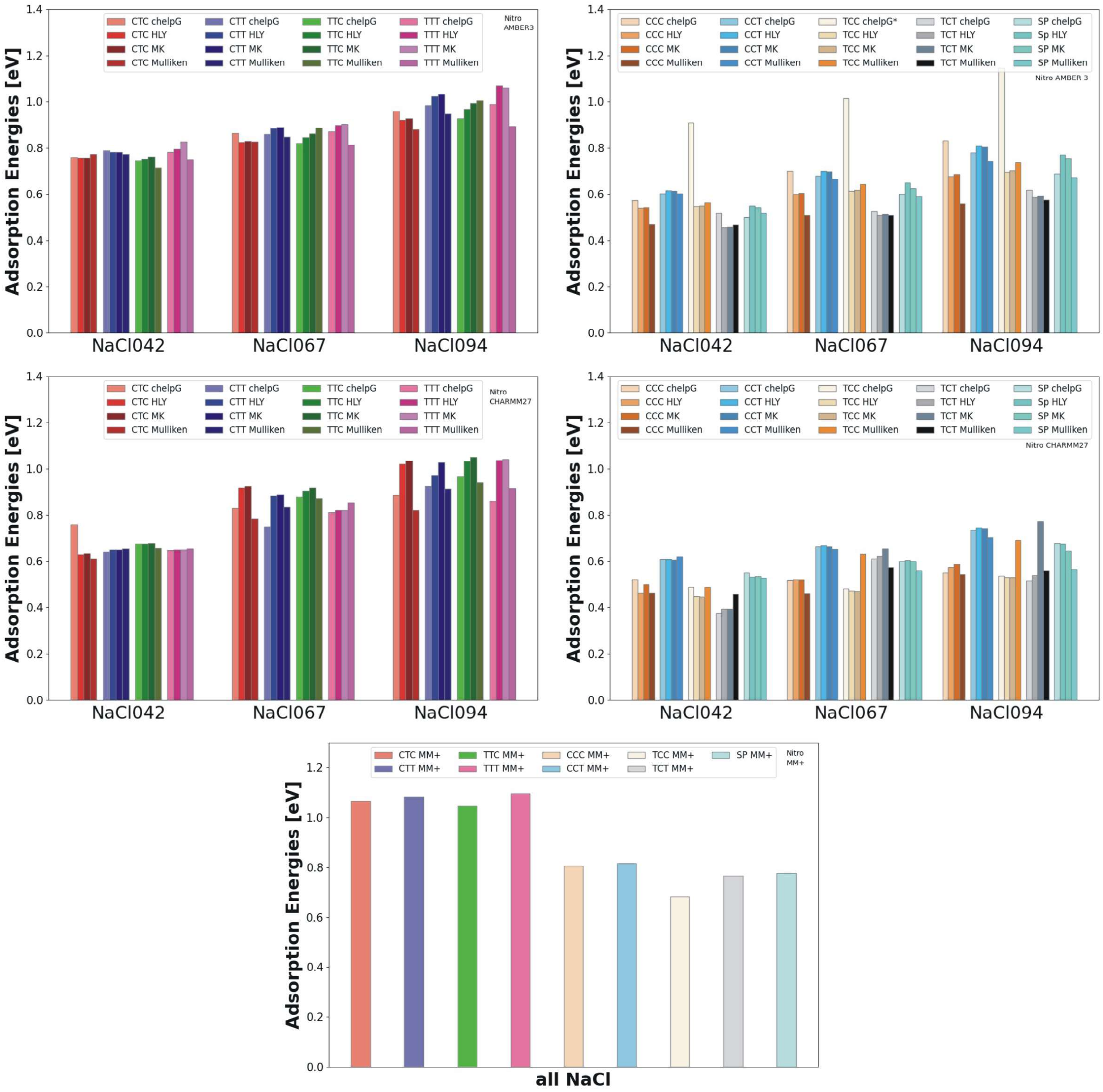

Figure S11: Comparison of adsorption energies for **Nitro** merocyanine conformers and spiropyran molecule using the **AMBER 3** force field (top), **CHARMM27** (middle) and **MM+** (bottom). The T-conformers (left graphs) have overall a higher adsorption energy than C-conformers and spiropyran (right graphs) due to the parallel orientation of the aromatic ring of the molecule with the substrate. Also clearly visible are the increased energies with respect to substrate polarity with lowest energy on a NaCl substrate with $\pm 0.42e$ as partial atomic charge on the sodium/chlorine atom, and highest adsorption energy for the substrate with $\pm 0.94e$ as partial charge. Noticeable are is also the nearly constant value for energies when using the various charge schemes. For MM+ (bottom), all adsorption energies for a given conformer were independent of the charge assignments on the substrate and the molecule. (*TCC conformer, using AMBER 3 and ChelpG, switched to CTC conformer when adsorbed.)

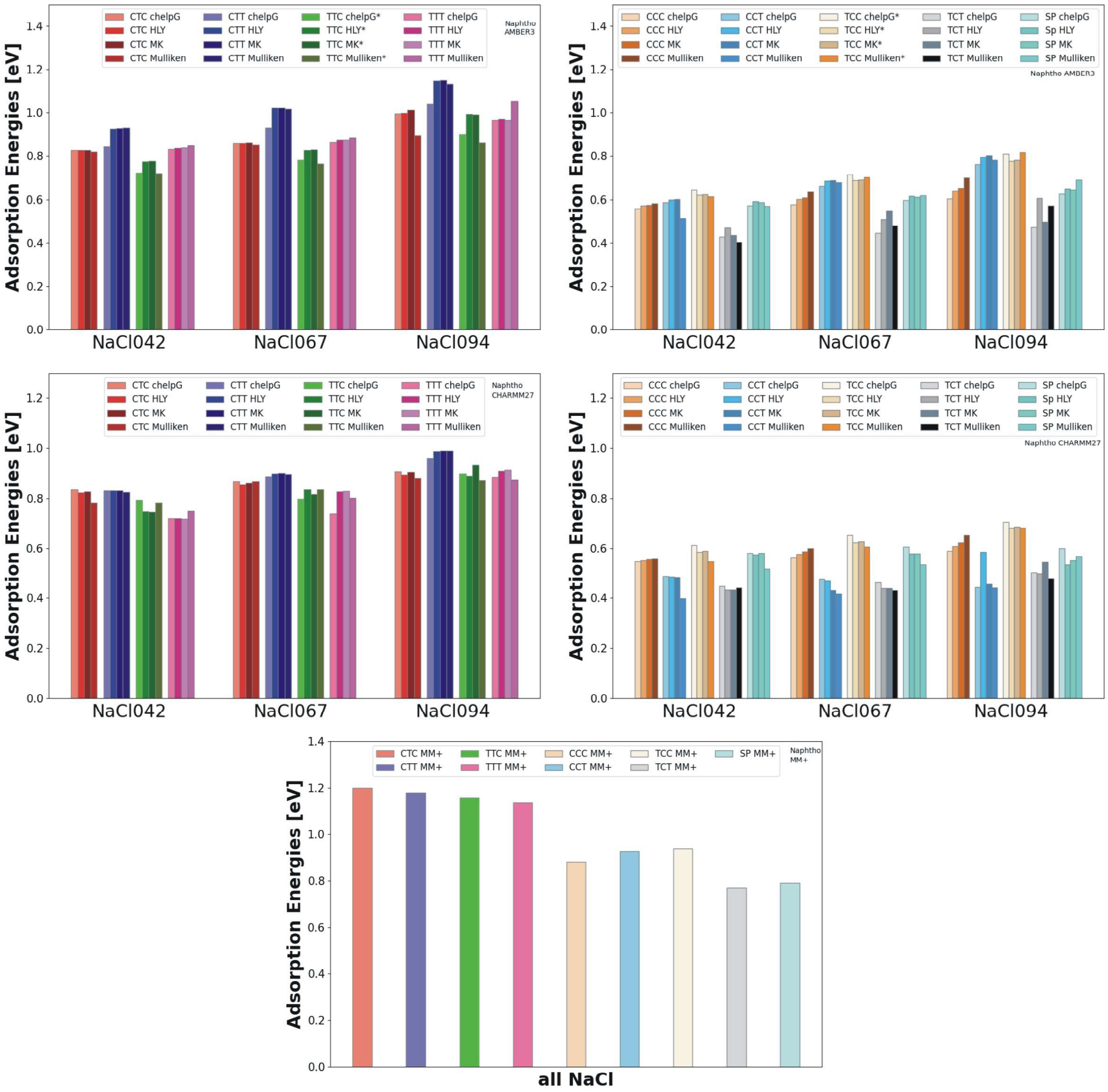

Figure S12: Comparison of adsorption energies for **Naphtho** merocyanine conformers and spiropyran molecule using the **AMBER 3** force field (top), **CHARMM27** (middle) and **MM+** (bottom). The T-conformers (left graphs) have overall a higher adsorption energy than C-conformers and spiropyran (right graphs) due to the parallel orientation of the aromatic ring of the molecule with the substrate. Also clearly visible are the increased energies with respect to substrate polarity with lowest energy on a NaCl substrate with $\pm 0.42e$ as partial atomic charge on the sodium/chlorine atom, and highest adsorption energy for the substrate with $\pm 0.94e$ as partial charge. Noticeable are is also the nearly constant value for energies when using the various charge schemes. For MM+ (bottom), all adsorption energies for a given conformer were independent of the charge assignments on the substrate and the molecule. (*TTC conformer using AMBER 3 , switched to CTT conformer when adsorbed, TCC conformer using AMBER 3 , switched to CCC conformer when adsorbed.)

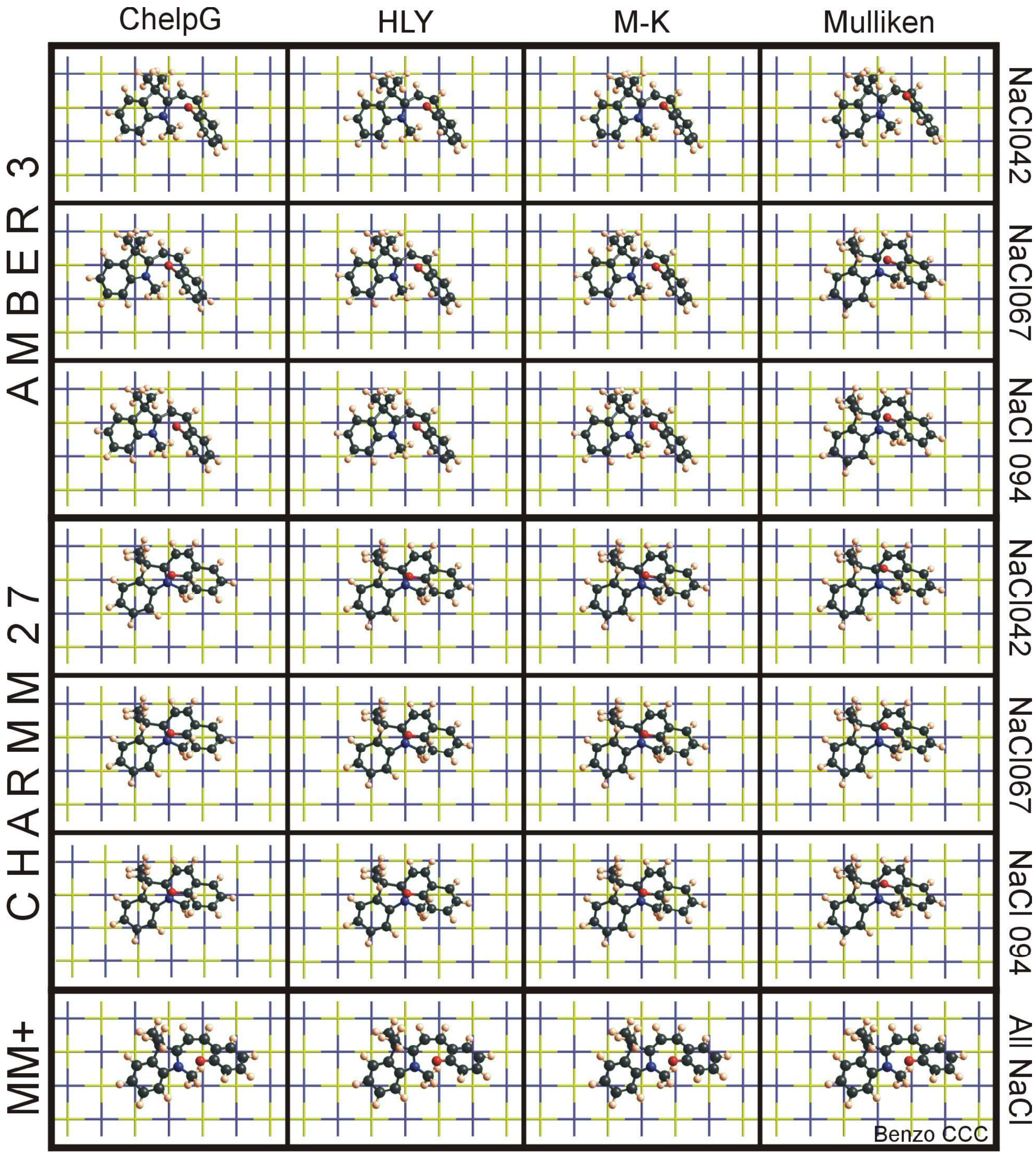

Figure S13: Adsorption geometry for **Benzo CCC conformer** using four charge methods (ChelpG, HLY, M-K, and Mulliken) and three force fields, AMBER 3 (top three rows), CHARMM27 (row four to six), and MM+(bottom row) and three substrate polarities (NaCl042 with Na/Cl atoms with $q = \pm 0.42e$, NaCl067 with Na/Cl atoms with $q = \pm 0.67e$, NaCl094 with Na/Cl atoms with $q = \pm 0.94e$.) For the force field MM+, there is no difference in geometry (and energy) when using different charge schemes for molecule or polarity for the substrate. The substrate's ionic bonds are rendered as tubes to show the grid: Chlorine (-) is yellow, and sodium (+) is blue/purple.

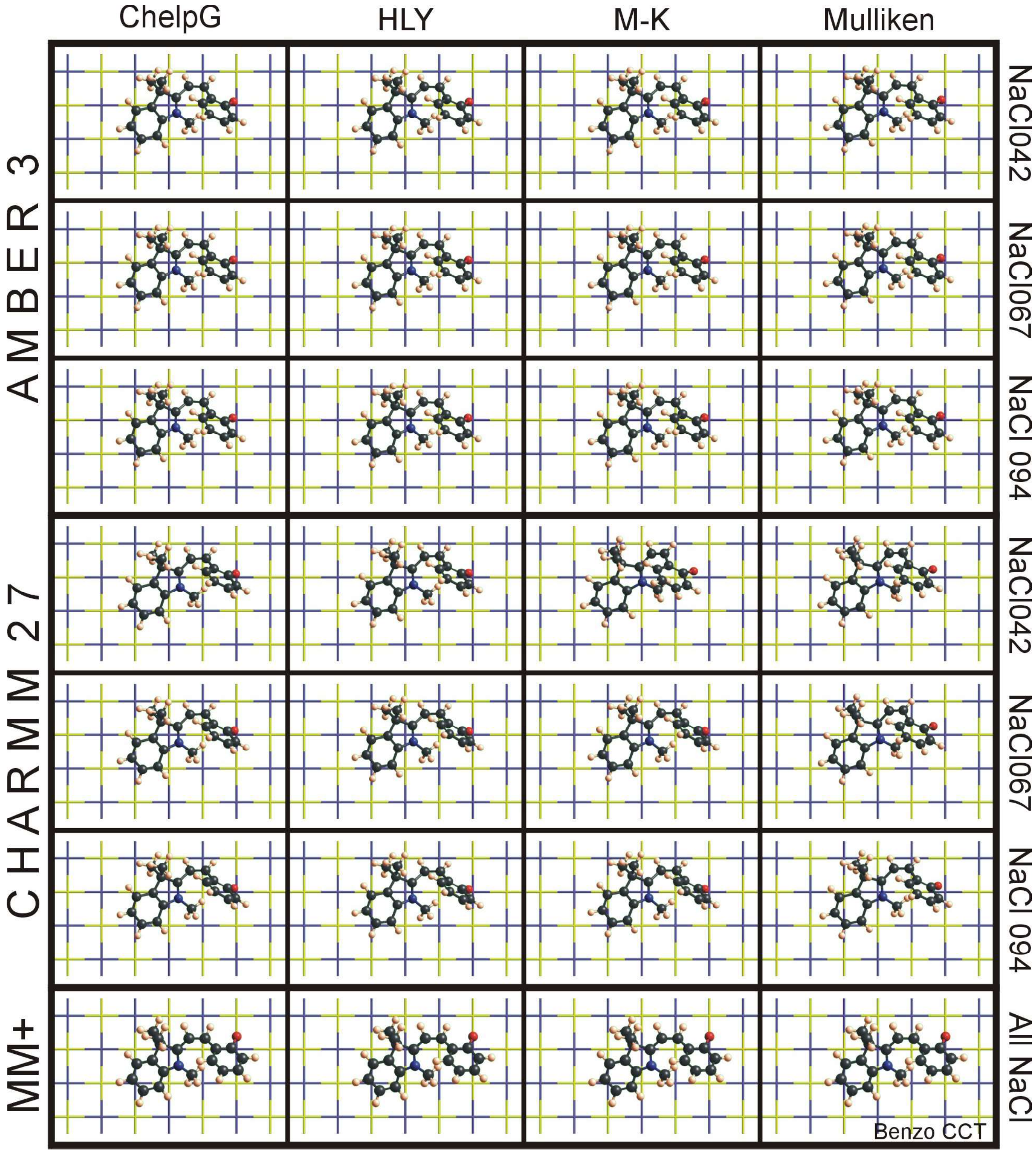

Figure S14: Adsorption geometry for **Benzo CCT conformer** using four charge methods (ChelpG, HLY, M-K, and Mulliken) and three force fields, AMBER 3 (top three rows), CHARMM27 (row four to six), and MM+(bottom row) and three substrate polarities (NaCl042 with Na/Cl atoms with $q = \pm 0.42e$, NaCl067 with Na/Cl atoms with $q = \pm 0.67e$, NaCl094 with Na/Cl atoms with $q = \pm 0.94e$.) For the force field MM+, there is no difference in geometry (and energy) when using different charge schemes for molecule or polarity for the substrate.

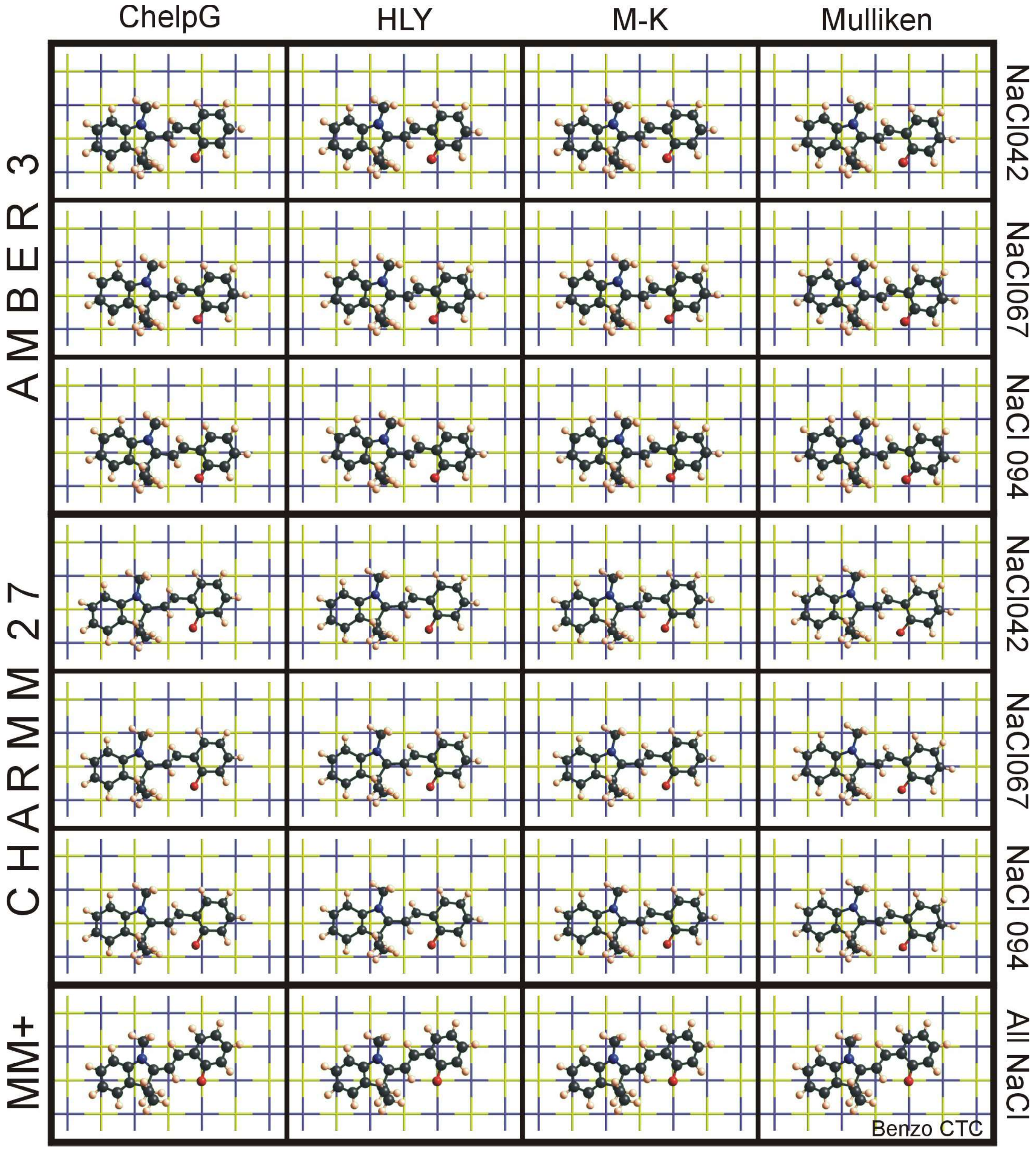

Figure S15: Adsorption geometry for **Benzo CTC conformer** using four charge methods (ChelpG, HLY, M-K, and Mulliken) and three force fields, AMBER 3 (top three rows), CHARMM27 (row four to six), and MM+(bottom row) and three substrate polarities (NaCl042 with Na/Cl atoms with $q = \pm 0.42e$, NaCl067 with Na/Cl atoms with $q = \pm 0.67e$, NaCl094 with Na/Cl atoms with $q = \pm 0.94e$.) For the force field MM+, there is no difference in geometry (and energy) when using different charge schemes for molecule or polarity for the substrate.

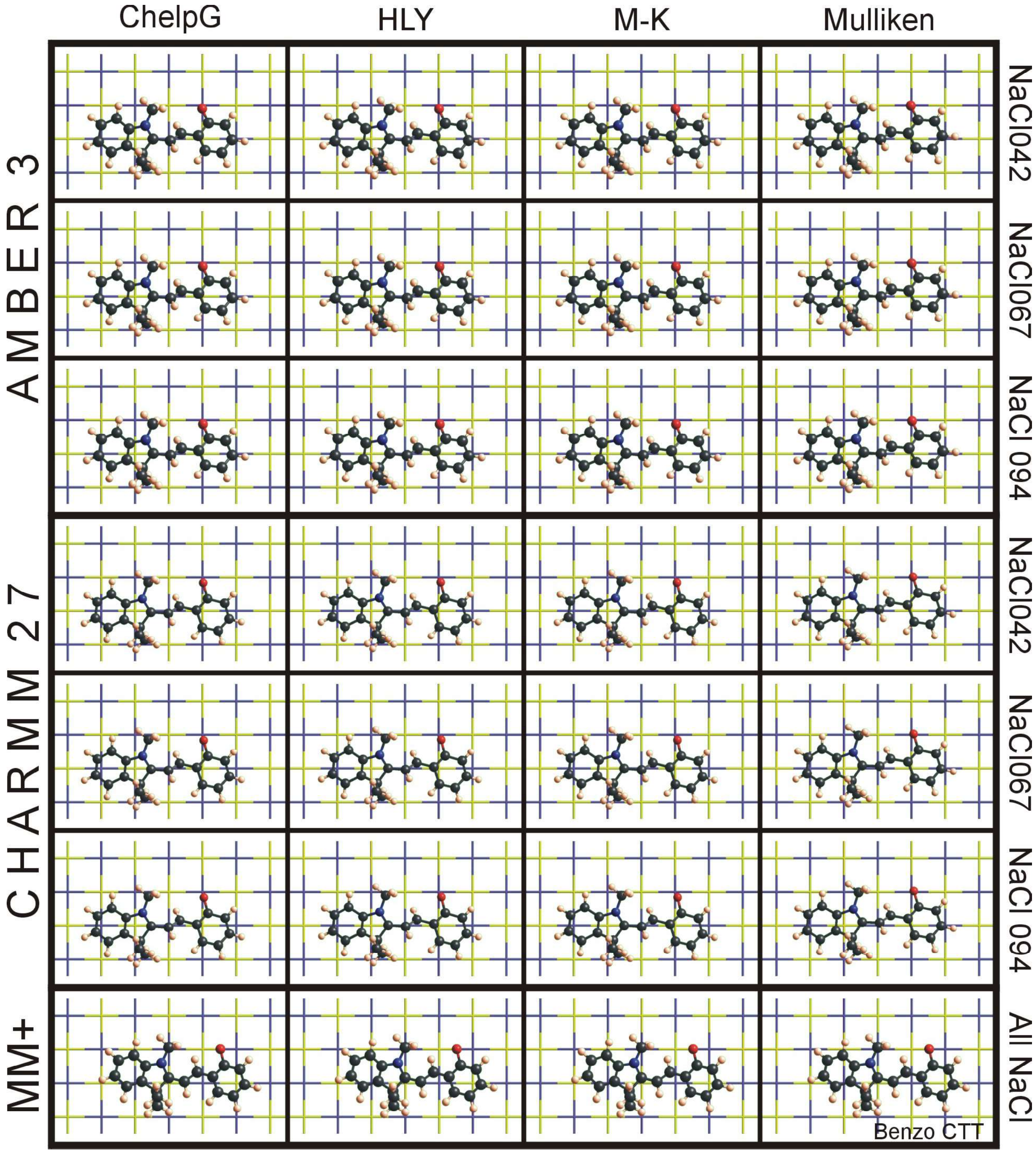

Figure S16: Adsorption geometry for **Benzo CTT conformer** using four charge methods (ChelpG, HLY, M-K, and Mulliken) and three force fields, AMBER 3 (top three rows), CHARMM27 (row four to six), and MM+(bottom row) and three substrate polarities (NaCl042 with Na/Cl atoms with $q = \pm 0.42e$, NaCl067 with Na/Cl atoms with $q = \pm 0.67e$, NaCl094 with Na/Cl atoms with $q = \pm 0.94e$.) For the force field MM+, there is no difference in geometry (and energy) when using different charge schemes for molecule or polarity for the substrate.

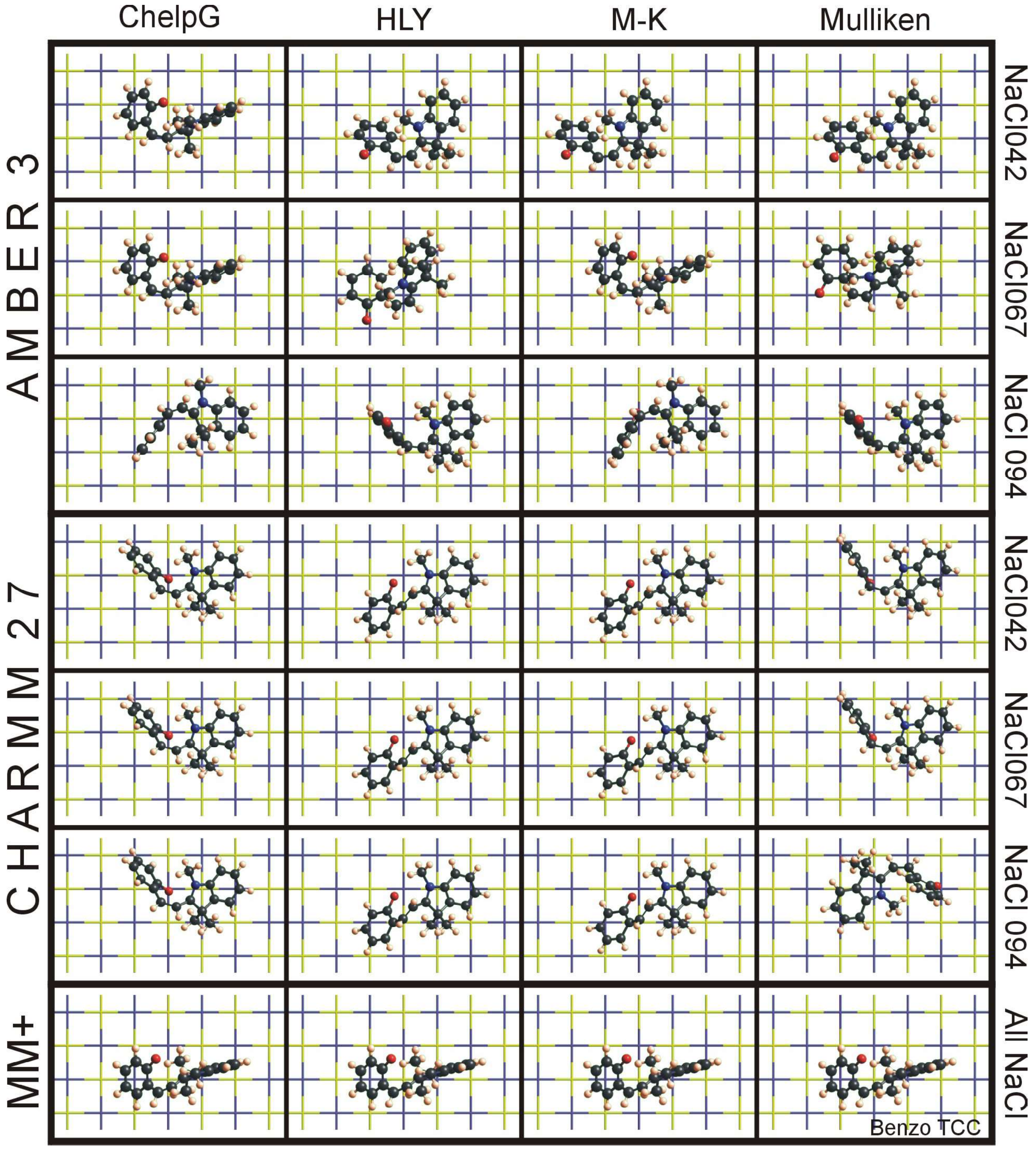

Figure S17: Adsorption geometry for **Benzo TCC conformer** using four charge methods (ChelpG, HLY, M-K, and Mulliken) and three force fields, AMBER 3 (top three rows), CHARMM27 (row four to six), and MM+(bottom row) and three substrate polarities (NaCl042 with Na/Cl atoms with $q = \pm 0.42e$, NaCl067 with Na/Cl atoms with $q = \pm 0.67e$, NaCl094 with Na/Cl atoms with $q = \pm 0.94e$.) For the force field MM+, there is no difference in geometry (and energy) when using different charge schemes for molecule or polarity for the substrate.

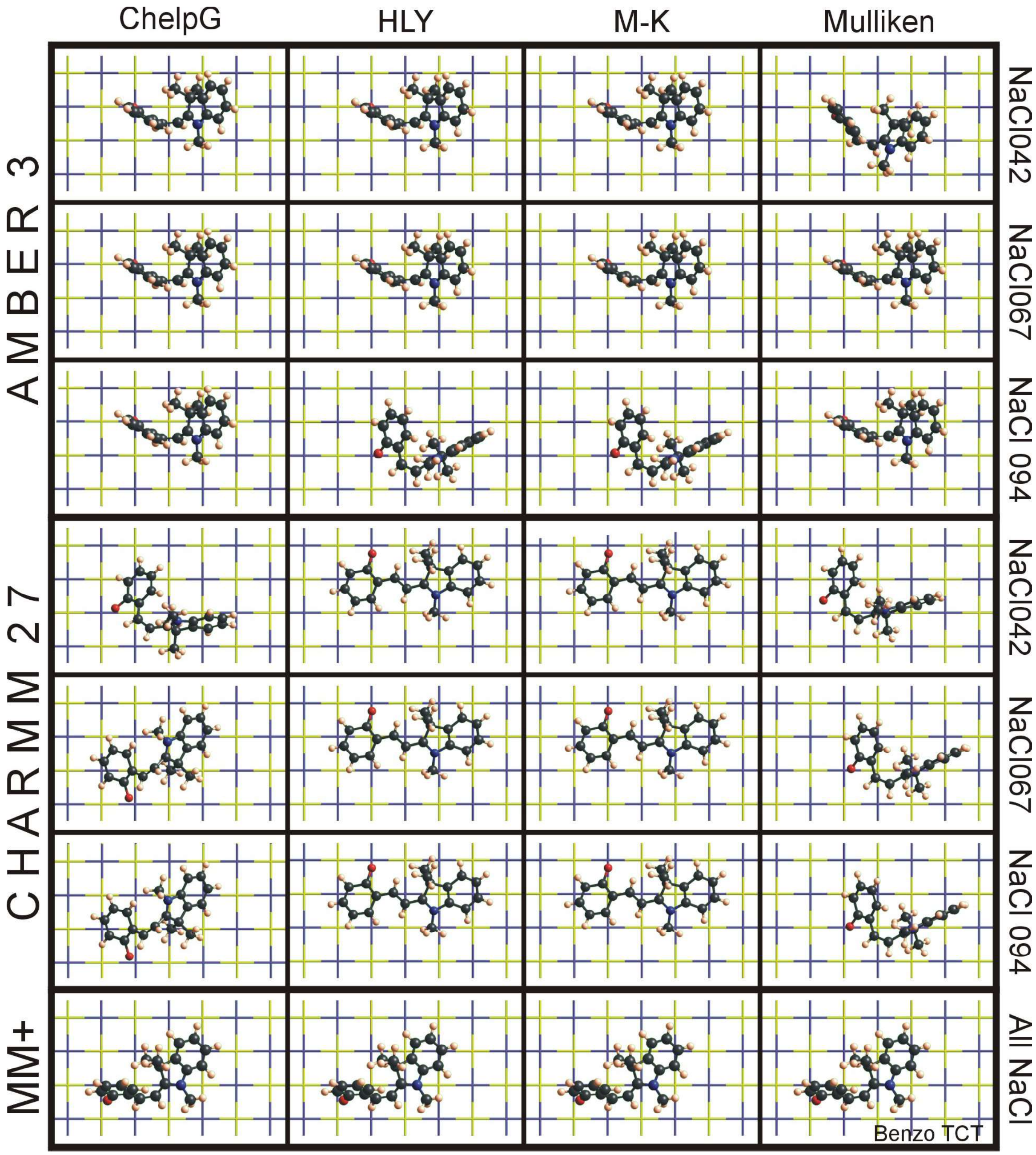

Figure S18: Adsorption geometry for **Benzo TCT conformer** using four charge methods (ChelpG, HLY, M-K, and Mulliken) and three force fields, AMBER 3 (top three rows), CHARMM27 (row four to six), and MM+(bottom row) and three substrate polarities (NaCl042 with Na/Cl atoms with $q = \pm 0.42e$, NaCl067 with Na/Cl atoms with $q = \pm 0.67e$, NaCl094 with Na/Cl atoms with $q = \pm 0.94e$.) For the force field MM+, there is no difference in geometry (and energy) when using different charge schemes for molecule or polarity for the substrate.

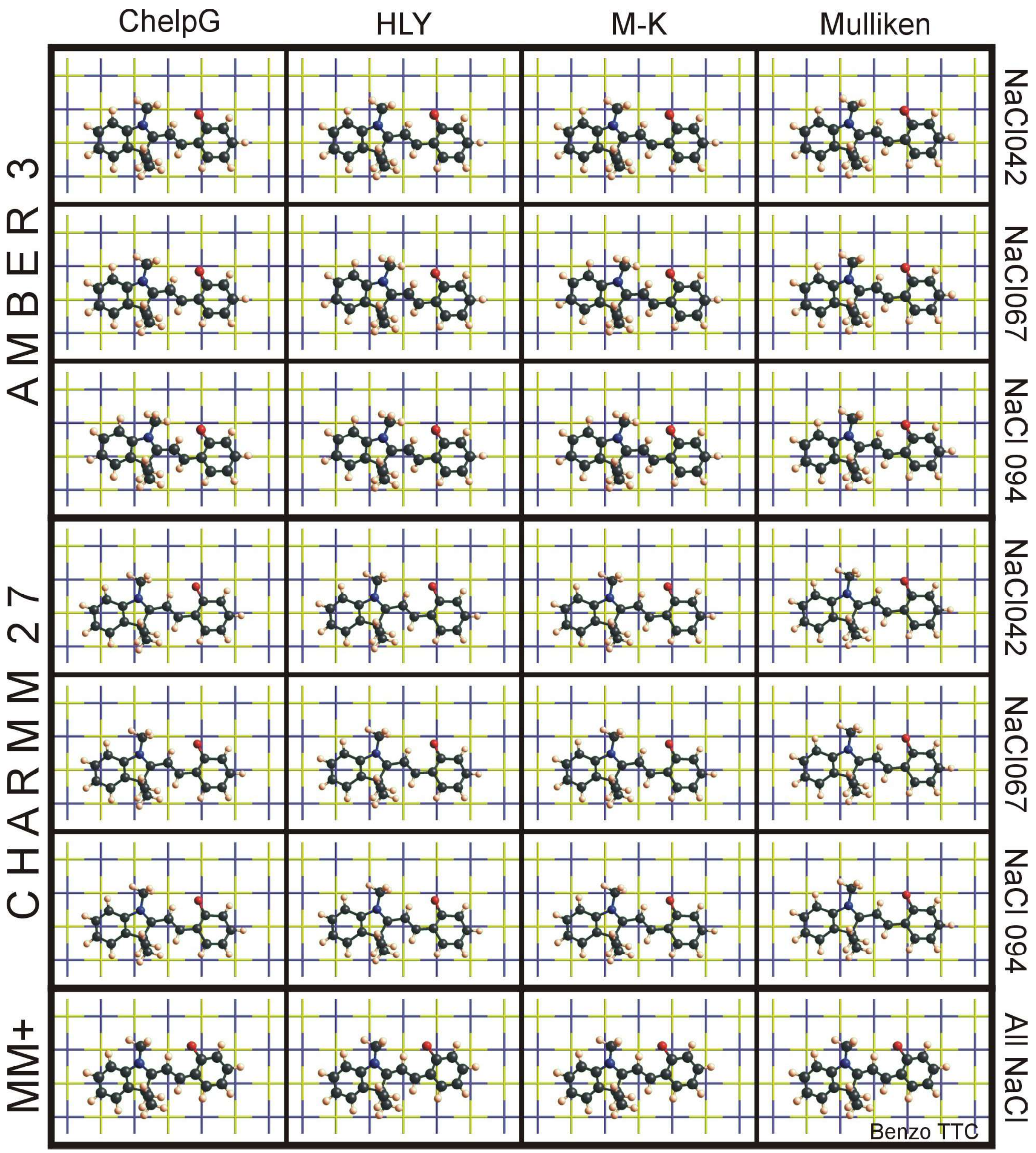

Figure S19: Adsorption geometry for **Benzo TTC conformer** using four charge methods (ChelpG, HLY, M-K, and Mulliken) and three force fields, AMBER3 (top three rows), CHARMM27 (row four to six), and MM+(bottom row) and three substrate polarities (NaCl042 with Na/Cl atoms with $q = \pm 0.42e$, NaCl067 with Na/Cl atoms with $q = \pm 0.67e$, NaCl094 with Na/Cl atoms with $q = \pm 0.94e$.) For the force field MM+, there is no difference in geometry (and energy) when using different charge schemes for molecule or polarity for the substrate.

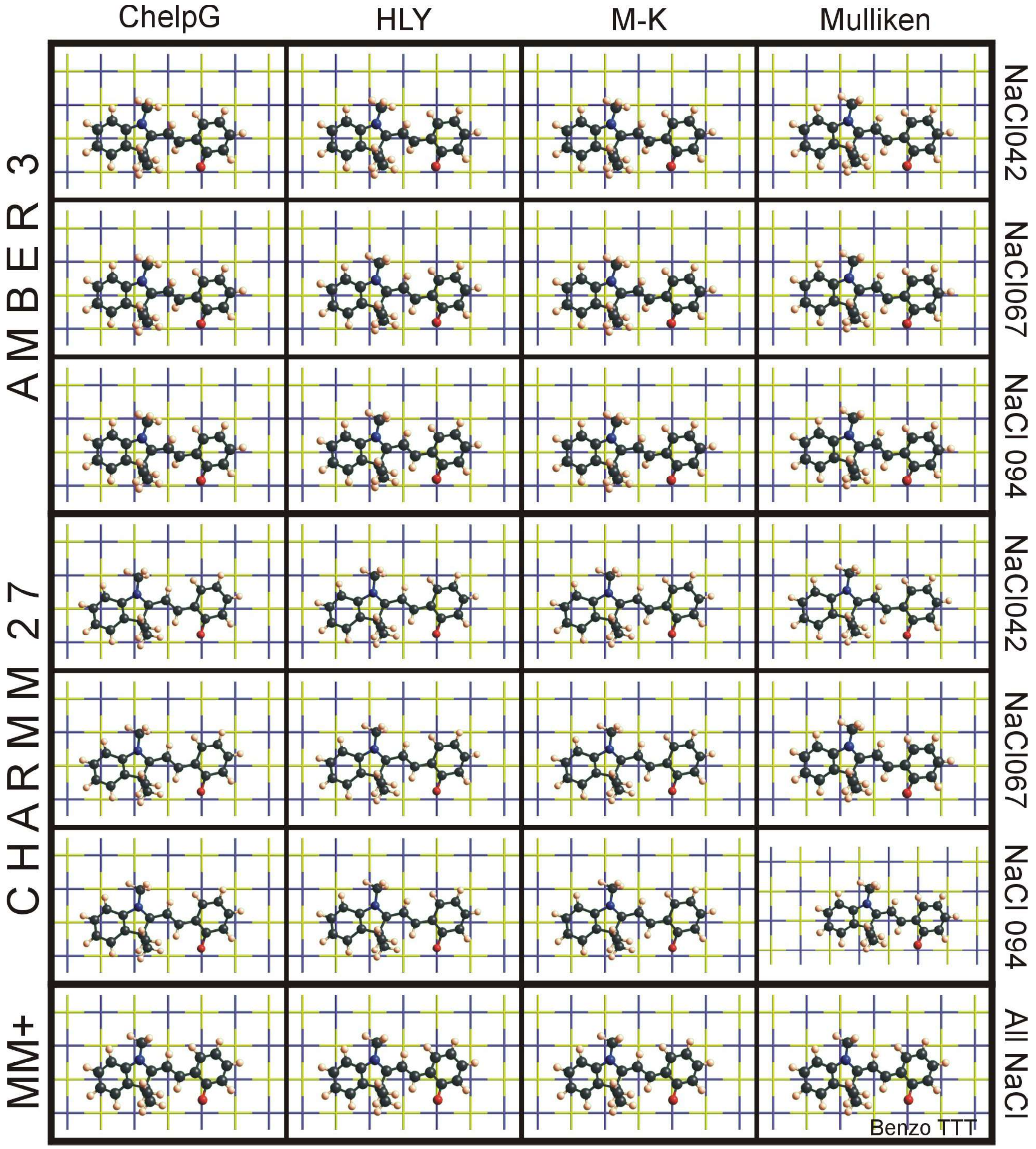

Figure S20: Adsorption geometry for **Benzo TTT conformer** using four charge methods (ChelpG, HLY, M-K, and Mulliken) and three force fields, AMBER 3 (top three rows), CHARMM27 (row four to six), and MM+(bottom row) and three substrate polarities (NaCl042 with Na/Cl atoms with $q = \pm 0.42e$, NaCl067 with Na/Cl atoms with $q = \pm 0.67e$, NaCl094 with Na/Cl atoms with $q = \pm 0.94e$.) For the force field MM+, there is no difference in geometry (and energy) when using different charge schemes for molecule or polarity for the substrate.

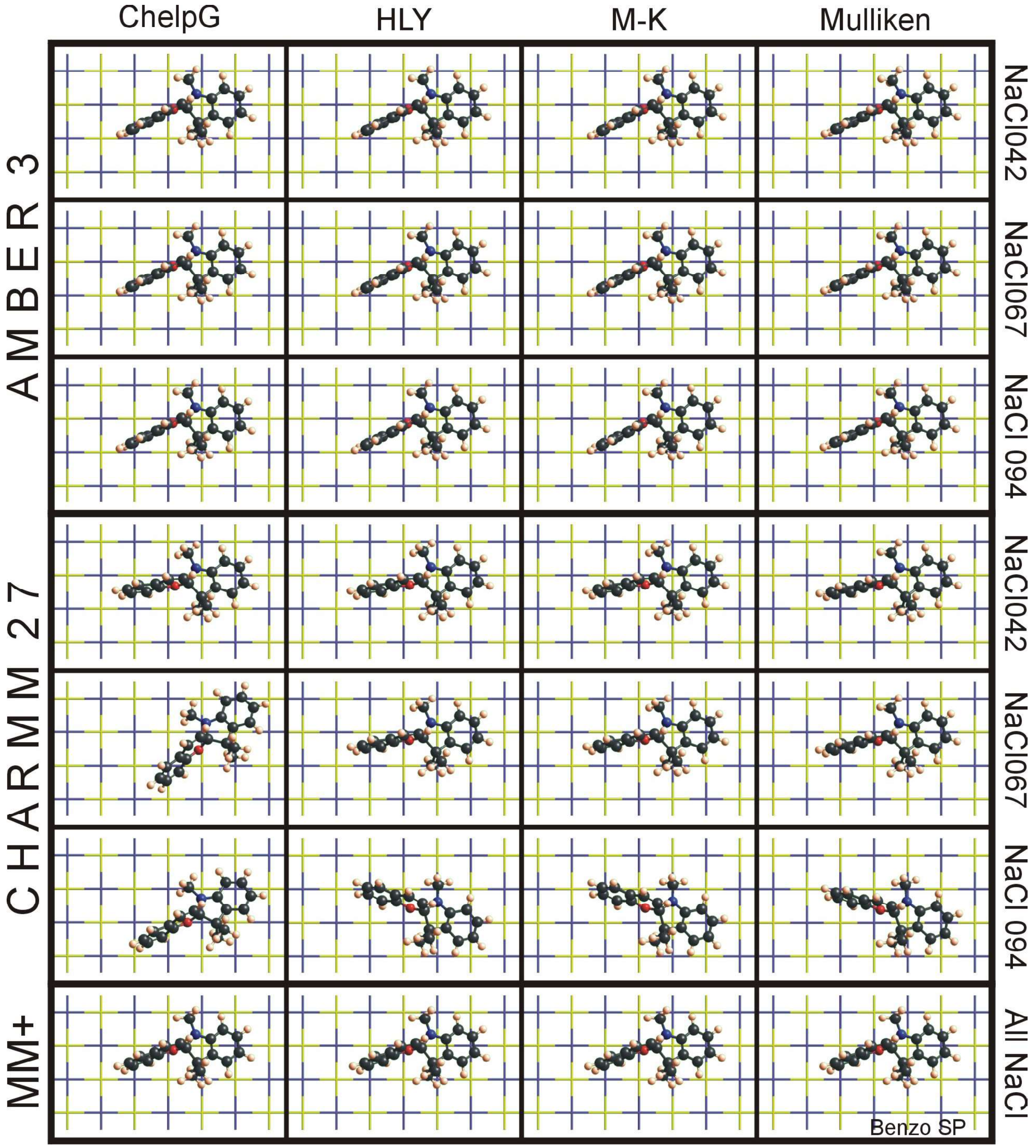

Figure S21: Adsorption geometry for **Benzo SP isomer** using four charge methods (ChelpG, HLY, M-K, and Mulliken) and three force fields, AMBER 3 (top three rows), CHARMM27 (row four to six), and MM+(bottom row) and three substrate polarities (NaCl042 with Na/Cl atoms with $q = \pm 0.42e$, NaCl067 with Na/Cl atoms with $q = \pm 0.67e$, NaCl094 with Na/Cl atoms with $q = \pm 0.94e$.) For the force field MM+, there is no difference in geometry (and energy) when using different charge schemes for molecule or polarity for the substrate.

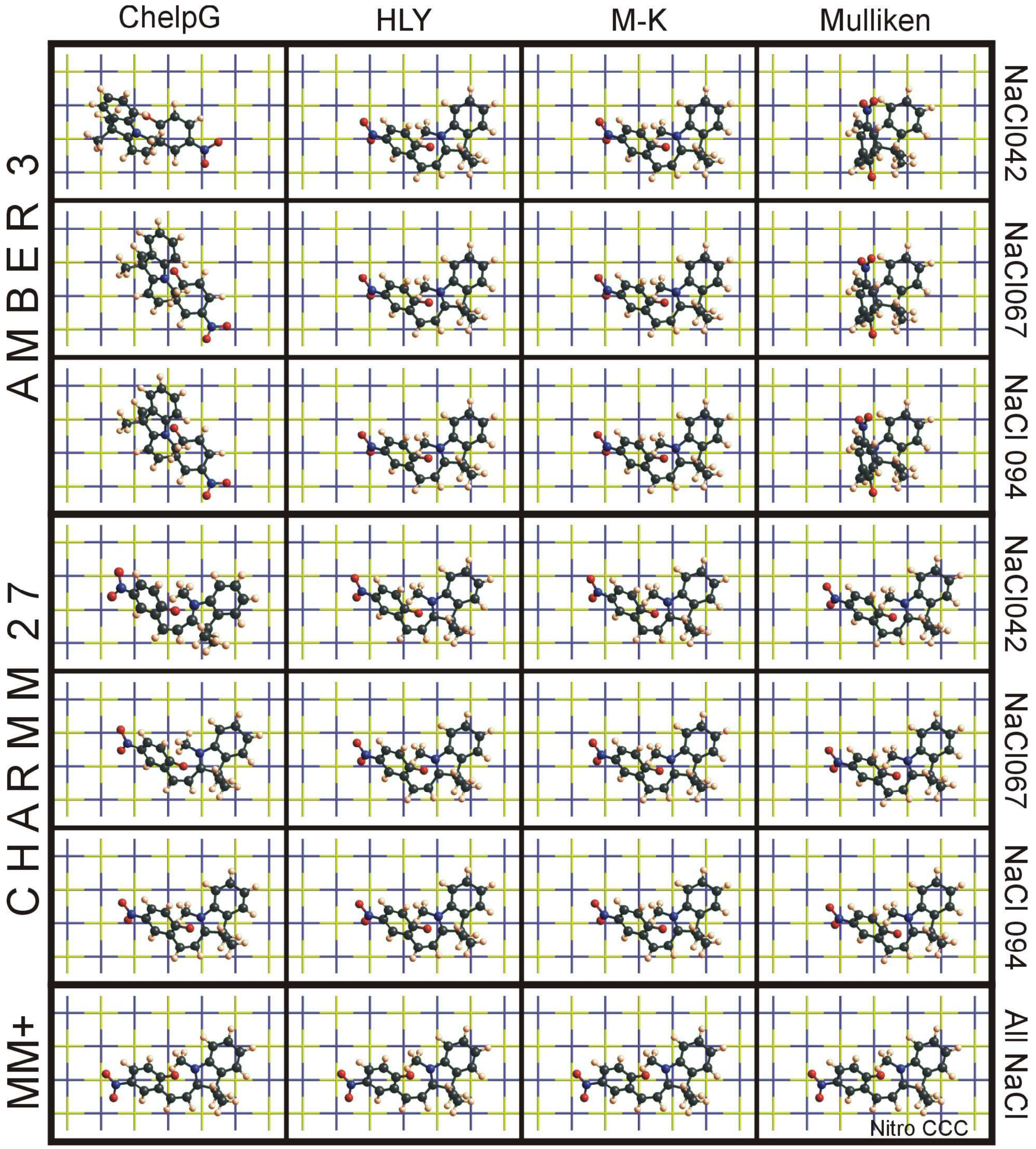

Figure S22: Adsorption geometry for **Nitro CCC conformer** using four charge methods (ChelpG, HLY, M-K, and Mulliken) and three force fields, AMBER 3 (top three rows), CHARMM27 (row four to six), and MM+(bottom row) and three substrate polarities (NaCl042 with Na/Cl atoms with $q = \pm 0.42e$, NaCl067 with Na/Cl atoms with $q = \pm 0.67e$, NaCl094 with Na/Cl atoms with $q = \pm 0.94e$.) For the force field MM+, there is no difference in geometry (and energy) when using different charge schemes for molecule or polarity for the substrate. The substrate's ionic bonds are rendered as tubes to show the grid: Chlorine (-) is yellow, and sodium (+) is blue/purple.

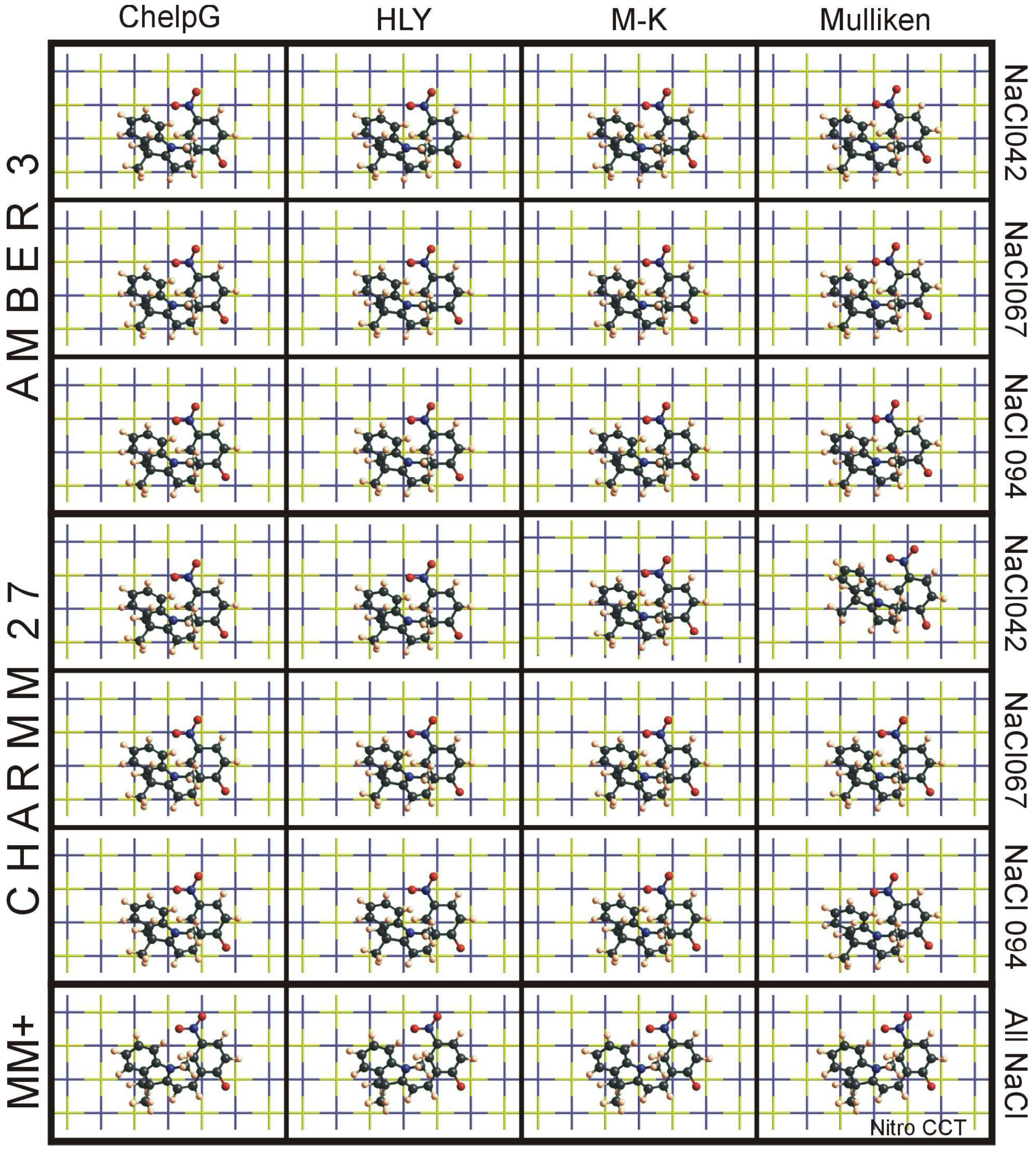

Figure S23: Adsorption geometry for **Nitro CCT conformer** using four charge methods (ChelpG, HLY, M-K, and Mulliken) and three force fields, AMBER 3 (top three rows), CHARMM27 (row four to six), and MM+(bottom row) and three substrate polarities (NaCl042 with Na/Cl atoms with $q = \pm 0.42e$, NaCl067 with Na/Cl atoms with $q = \pm 0.67e$, NaCl094 with Na/Cl atoms with $q = \pm 0.94e$.) For the force field MM+, there is no difference in geometry (and energy) when using different charge schemes for molecule or polarity for the substrate.

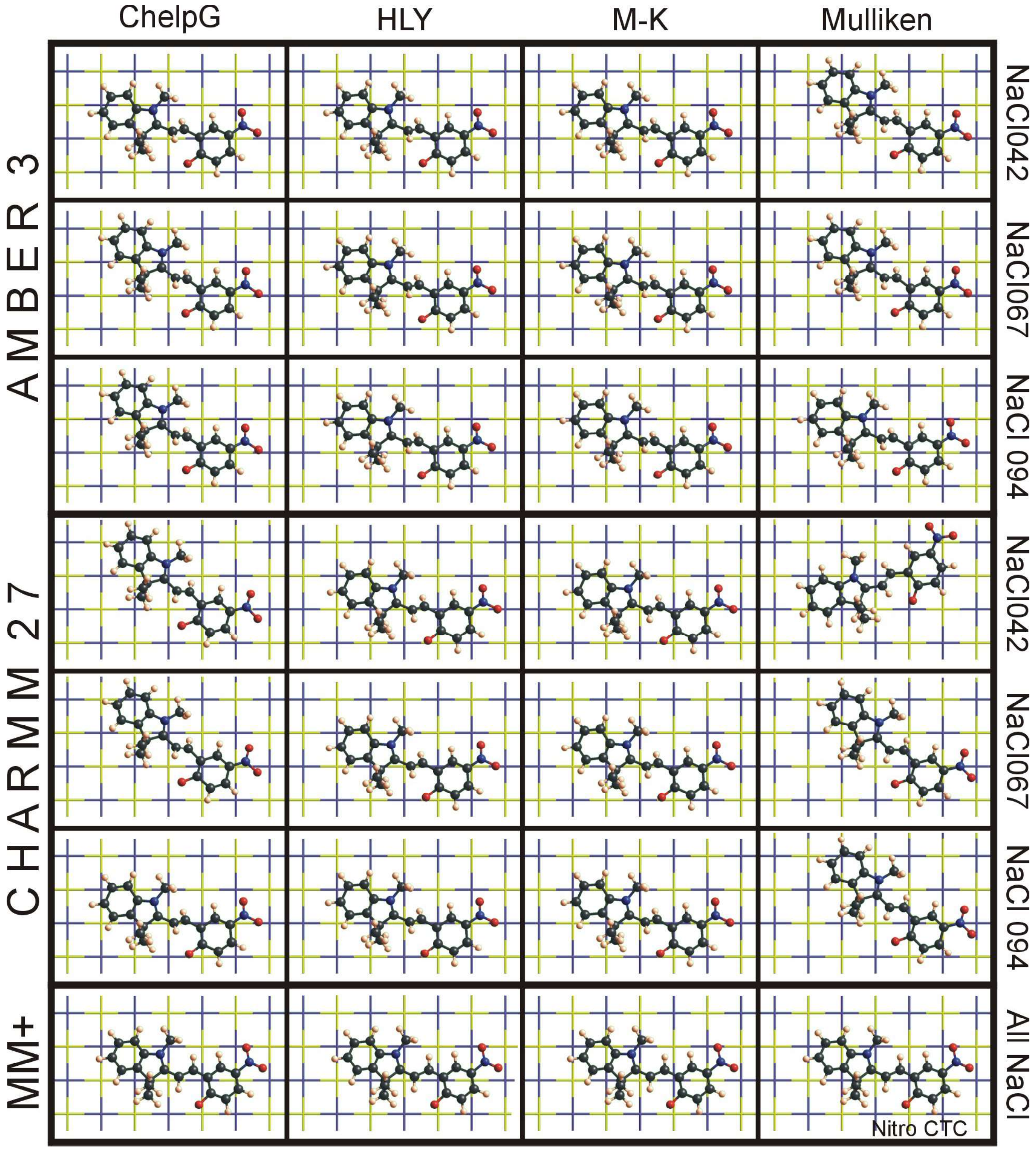

Figure S24: Adsorption geometry for **Nitro CTC conformer** using four charge methods (ChelpG, HLY, M-K, and Mulliken) and three force fields, AMBER 3 (top three rows), CHARMM27 (row four to six), and MM+(bottom row) and three substrate polarities (NaCl042 with Na/Cl atoms with $q = \pm 0.42e$, NaCl067 with Na/Cl atoms with $q = \pm 0.67e$, NaCl094 with Na/Cl atoms with $q = \pm 0.94e$.) For the force field MM+, there is no difference in geometry (and energy) when using different charge schemes for molecule or polarity for the substrate.

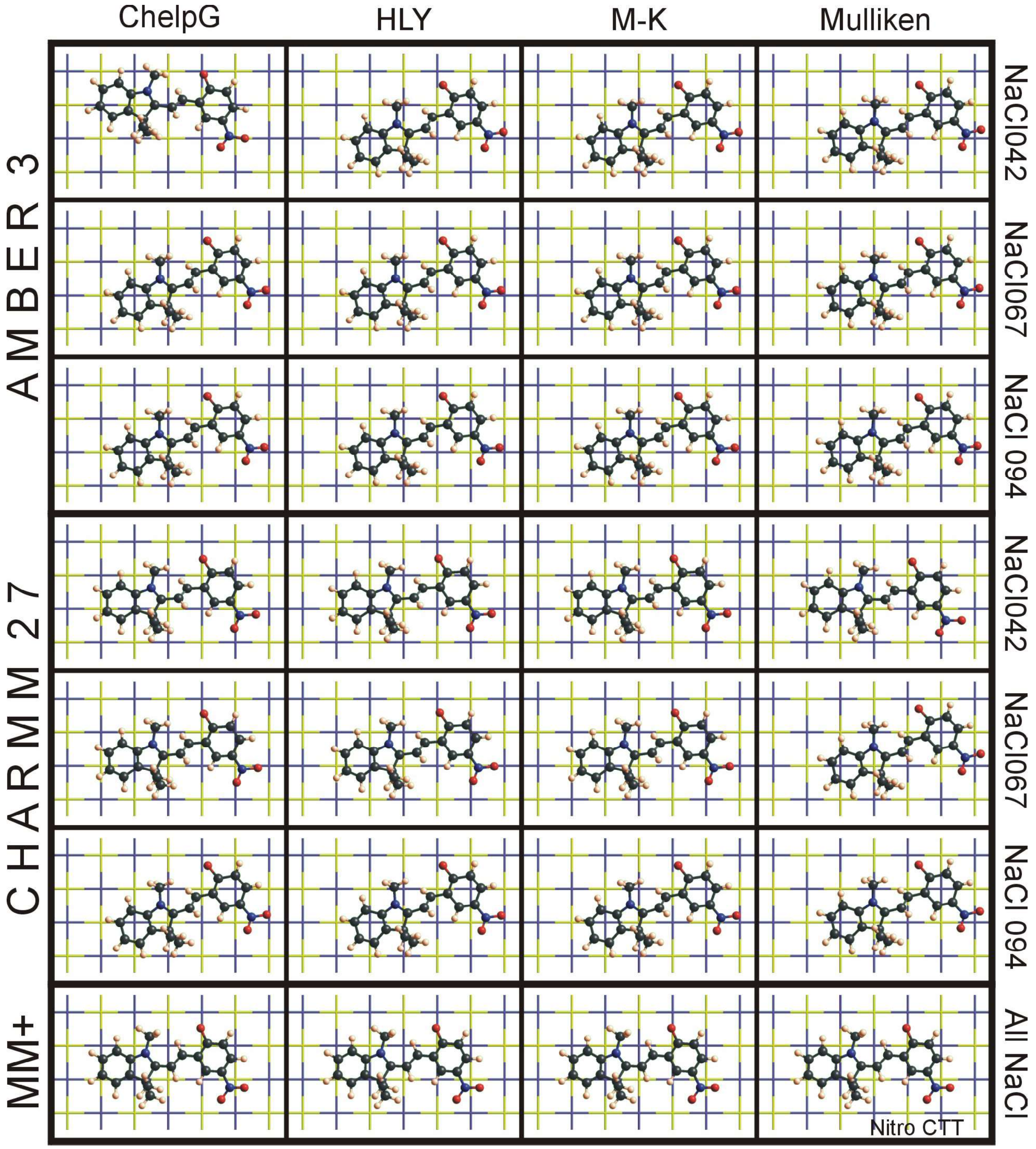

Figure S25: Adsorption geometry for **Nitro CTT conformer** using four charge methods (ChelpG, HLY, M-K, and Mulliken) and three force fields, AMBER3 (top three rows), CHARMM27 (row four to six), and MM+(bottom row) and three substrate polarities (NaCl042 with Na/Cl atoms with $q = \pm 0.42e$, NaCl067 with Na/Cl atoms with $q = \pm 0.67e$, NaCl094 with Na/Cl atoms with $q = \pm 0.94e$.) For the force field MM+, there is no difference in geometry (and energy) when using different charge schemes for molecule or polarity for the substrate.

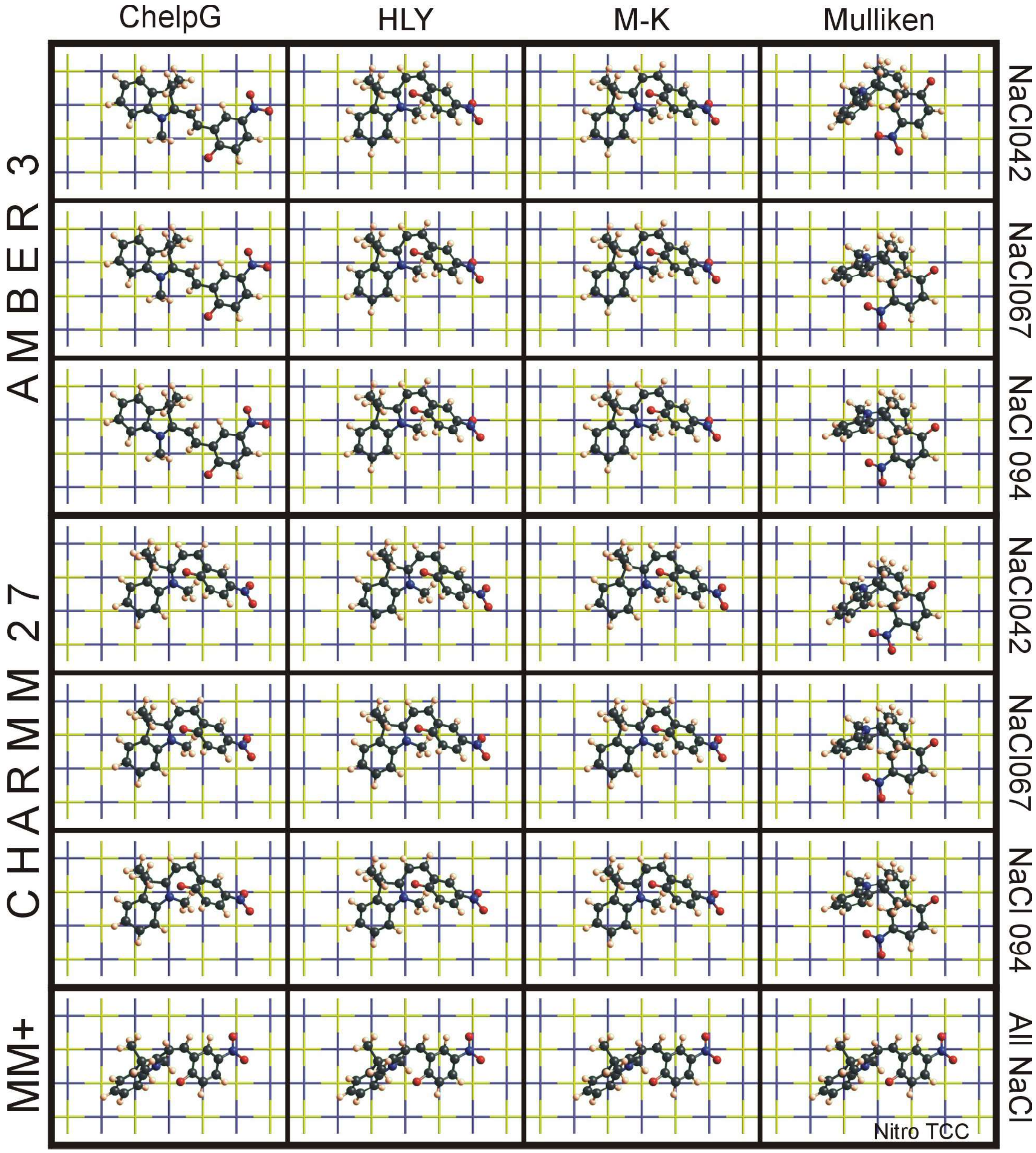

Figure S26: Adsorption geometry for **Nitro TCC conformer** using four charge methods (ChelpG, HLY, M-K, and Mulliken) and three force fields, AMBER 3 (top three rows), CHARMM27 (row four to six), and MM+(bottom row) and three substrate polarities (NaCl042 with Na/Cl atoms with $q = \pm 0.42e$, NaCl067 with Na/Cl atoms with $q = \pm 0.67e$, NaCl094 with Na/Cl atoms with $q = \pm 0.94e$.) For the force field MM+, there is no difference in geometry (and energy) when using different charge schemes for molecule or polarity for the substrate.

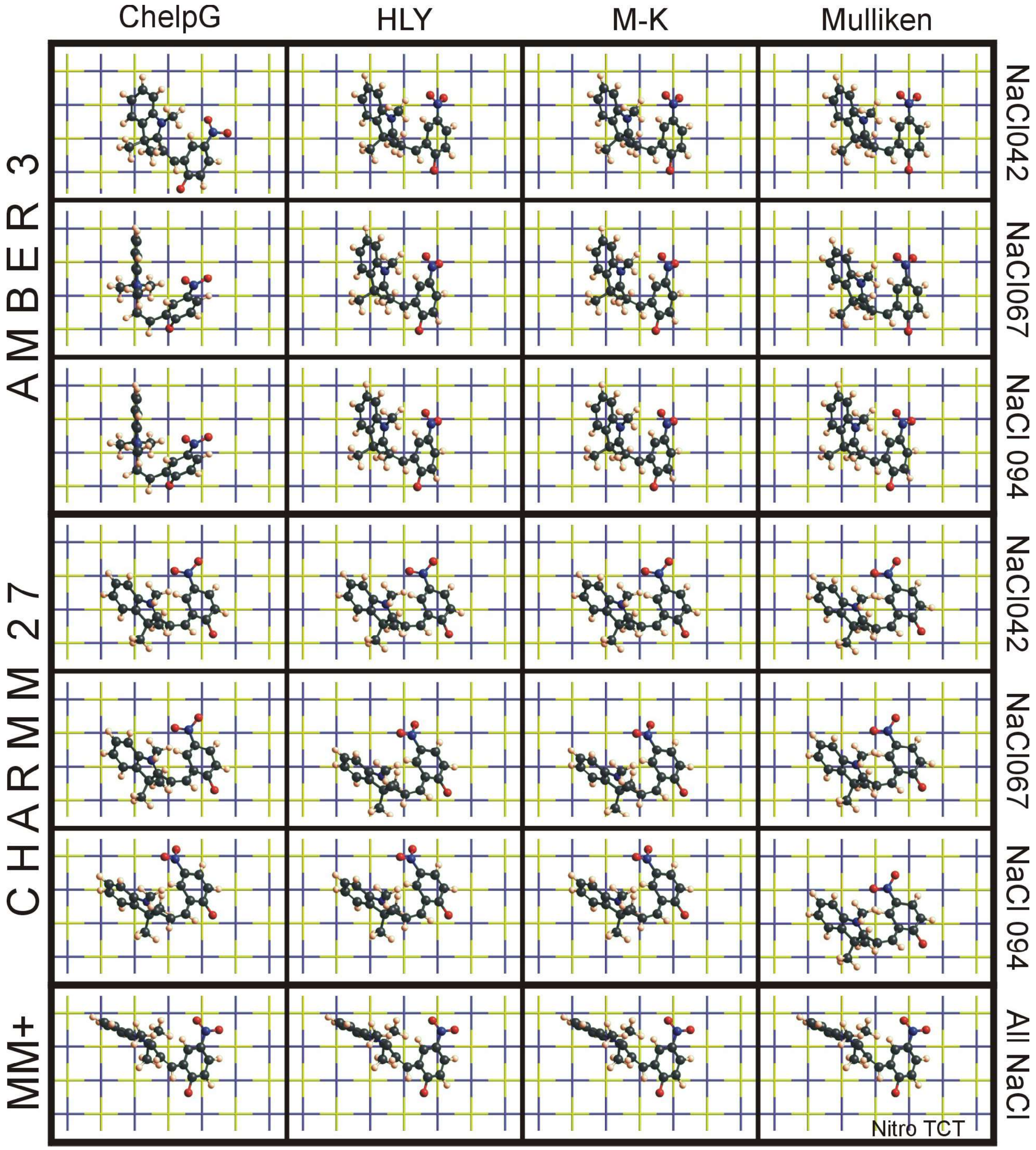

Figure S27: Adsorption geometry for **Nitro TCT conformer** using four charge methods (ChelpG, HLY, M-K, and Mulliken) and three force fields, AMBER 3 (top three rows), CHARMM27 (row four to six), and MM+(bottom row) and three substrate polarities (NaCl042 with Na/Cl atoms with $q = \pm 0.42e$, NaCl067 with Na/Cl atoms with $q = \pm 0.67e$, NaCl094 with Na/Cl atoms with $q = \pm 0.94e$.) For the force field MM+, there is no difference in geometry (and energy) when using different charge schemes for molecule or polarity for the substrate.

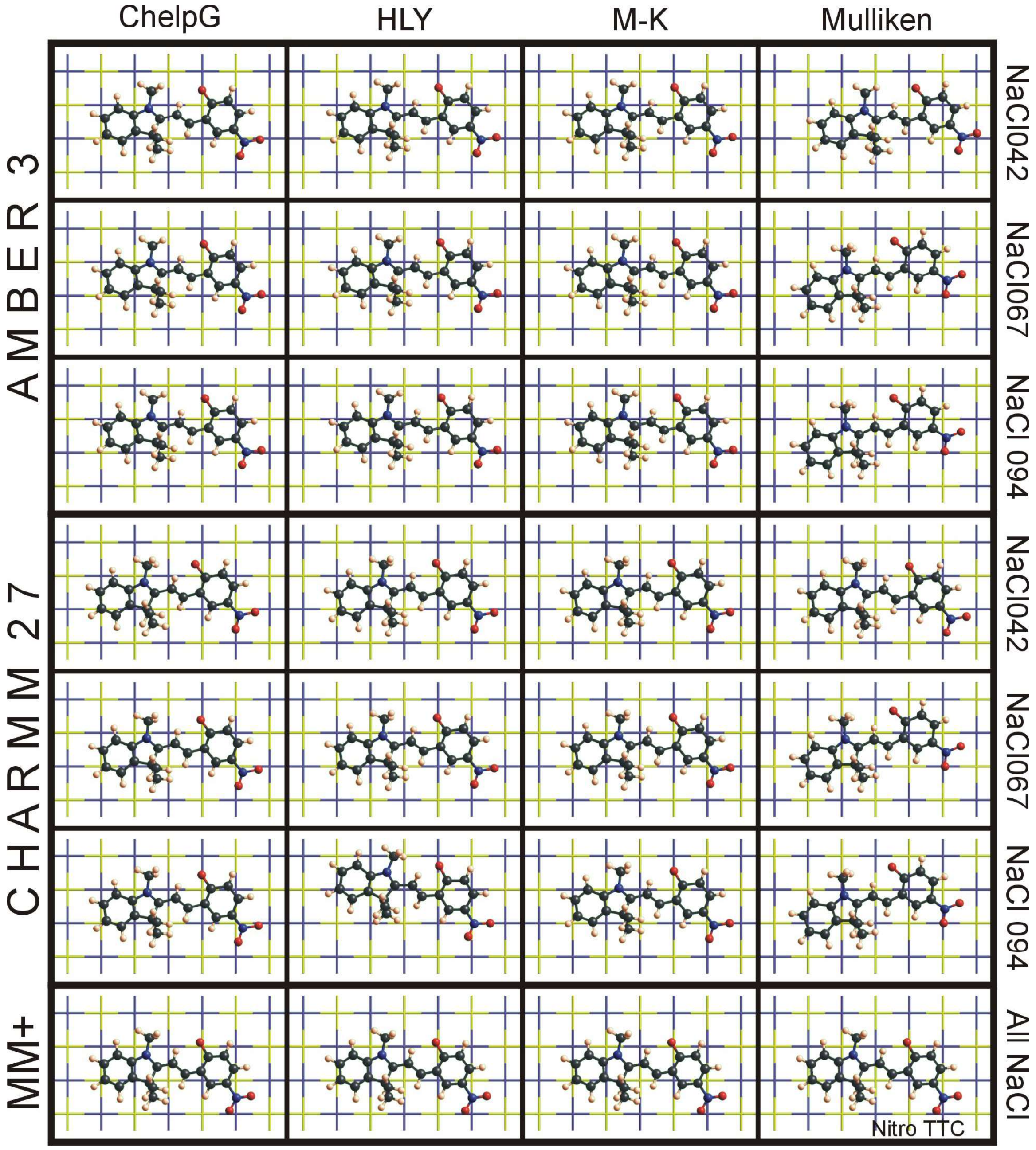

Figure S28: Adsorption geometry for **Nitro TTC conformer** using four charge methods (ChelpG, HLY, M-K, and Mulliken) and three force fields, AMBER3 (top three rows), CHARMM27 (row four to six), and MM+(bottom row) and three substrate polarities (NaCl042 with Na/Cl atoms with $q = \pm 0.42e$, NaCl067 with Na/Cl atoms with $q = \pm 0.67e$, NaCl094 with Na/Cl atoms with $q = \pm 0.94e$.) For the force field MM+, there is no difference in geometry (and energy) when using different charge schemes for molecule or polarity for the substrate.

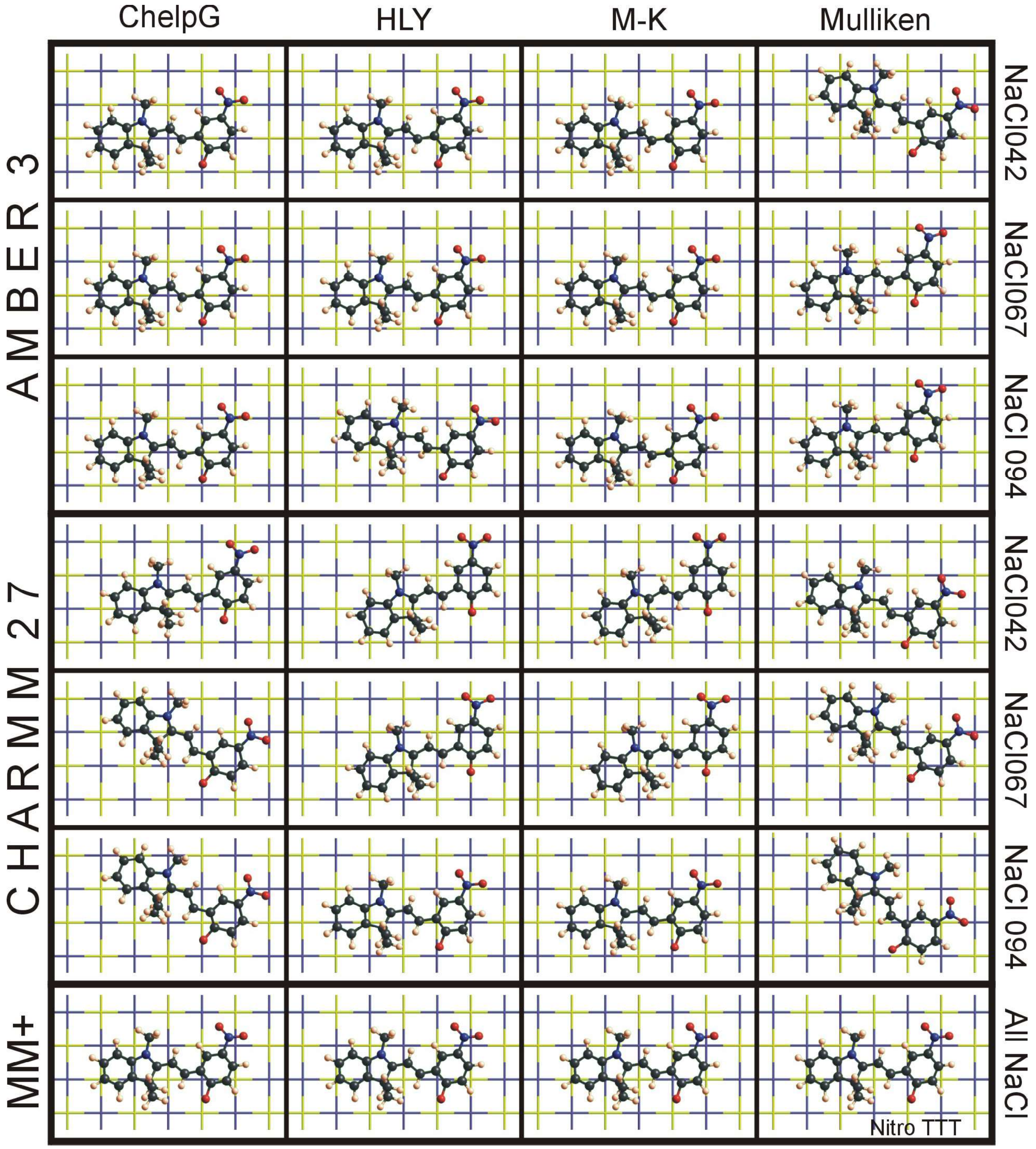

Figure S29: Adsorption geometry for **Nitro TTT conformer** using four charge methods (ChelpG, HLY, M-K, and Mulliken) and three force fields, AMBER 3 (top three rows), CHARMM27 (row four to six), and MM+(bottom row) and three substrate polarities (NaCl042 with Na/Cl atoms with $q = \pm 0.42e$, NaCl067 with Na/Cl atoms with $q = \pm 0.67e$, NaCl094 with Na/Cl atoms with $q = \pm 0.94e$.) For the force field MM+, there is no difference in geometry (and energy) when using different charge schemes for molecule or polarity for the substrate.

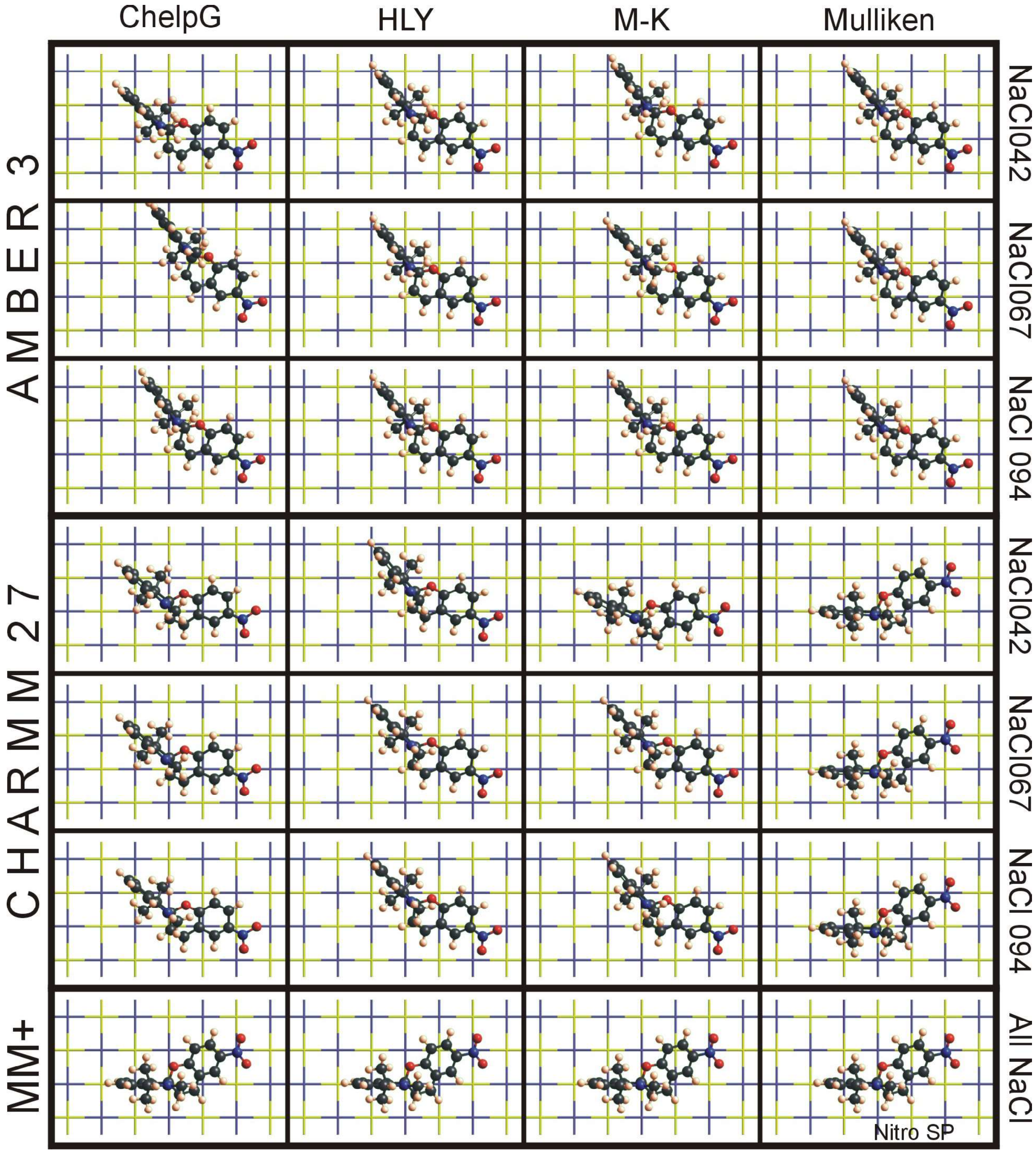

Figure S30: Adsorption geometry for **Nitro SP isomer** using four charge methods (ChelpG, HLY, M-K, and Mulliken) and three force fields, AMBER 3 (top three rows), CHARMM27 (row four to six), and MM+(bottom row) and three substrate polarities (NaCl042 with Na/Cl atoms with $q = \pm 0.42e$, NaCl067 with Na/Cl atoms with $q = \pm 0.67e$, NaCl094 with Na/Cl atoms with $q = \pm 0.94e$.) For the force field MM+, there is no difference in geometry (and energy) when using different charge schemes for molecule or polarity for the substrate.

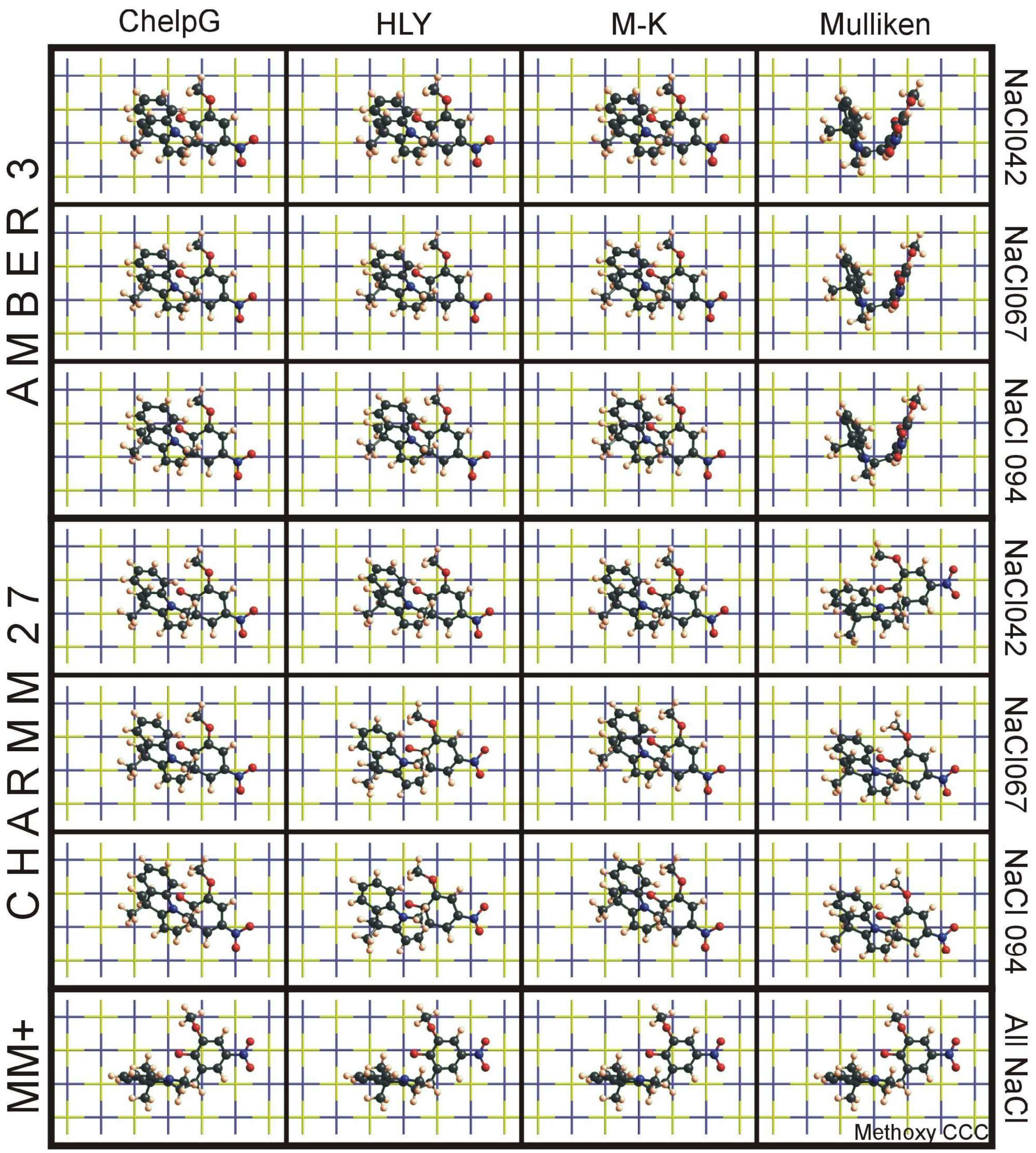

Figure S31: Adsorption geometry for **Methoxy CCC conformer** using four charge methods (ChelpG, HLY, M-K, and Mulliken) and three force fields, AMBER 3 (top three rows), CHARMM27 (row four to six), and MM+(bottom row) and three substrate polarities (NaCl042 with Na/Cl atoms with $q = \pm 0.42e$, NaCl067 with Na/Cl atoms with $q = \pm 0.67e$, NaCl094 with Na/Cl atoms with $q = \pm 0.94e$.) For the force field MM+, there is no difference in geometry (and energy) when using different charge schemes for molecule or polarity for the substrate. The substrate's ionic bonds are rendered as tubes to show the grid: Chlorine (-) is yellow, and sodium (+) is blue/purple.

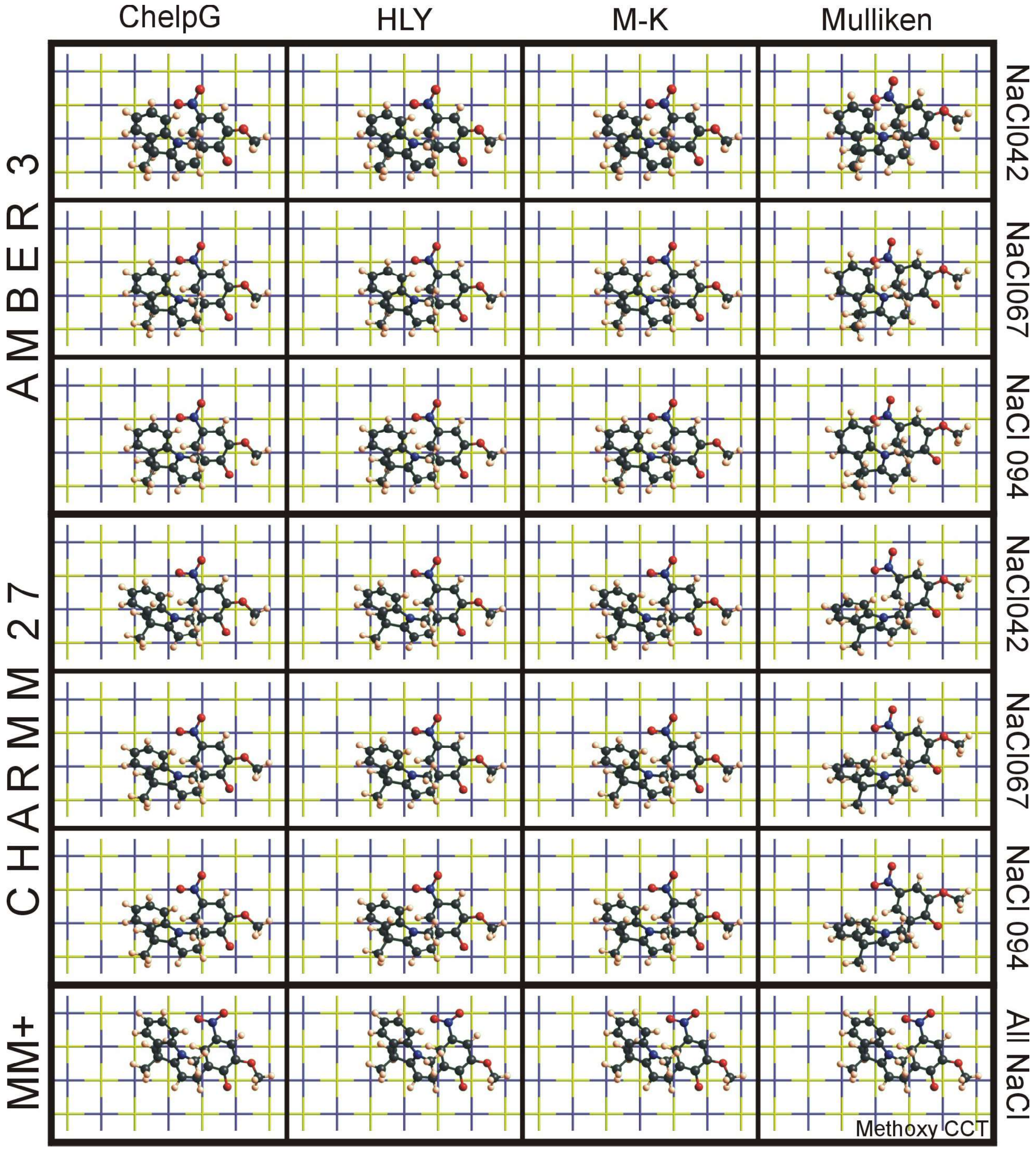

Figure S32: Adsorption geometry for **Methoxy CCT conformer** using four charge methods (ChelpG, HLY, M-K, and Mulliken) and three force fields, AMBER 3 (top three rows), CHARMM27 (row four to six), and MM+(bottom row) and three substrate polarities (NaCl042 with Na/Cl atoms with $q = \pm 0.42e$, NaCl067 with Na/Cl atoms with $q = \pm 0.67e$, NaCl094 with Na/Cl atoms with $q = \pm 0.94e$.) For the force field MM+, there is no difference in geometry (and energy) when using different charge schemes for molecule or polarity for the substrate.

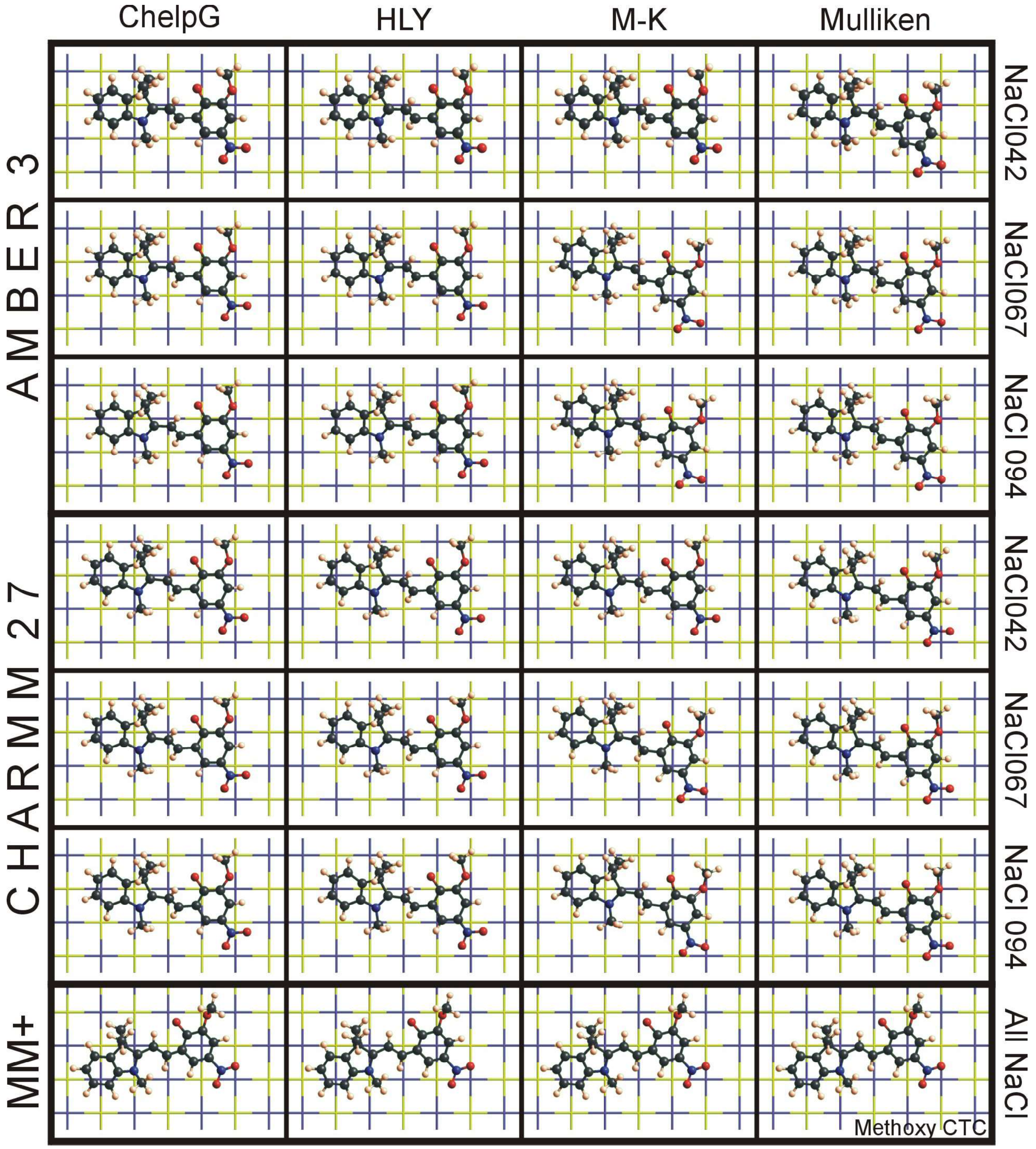

Figure S33: Adsorption geometry for **Methoxy CTC conformer** using four charge methods (ChelpG, HLY, M-K, and Mulliken) and three force fields, AMBER 3 (top three rows), CHARMM27 (row four to six), and MM+(bottom row) and three substrate polarities (NaCl042 with Na/Cl atoms with $q = \pm 0.42e$, NaCl067 with Na/Cl atoms with $q = \pm 0.67e$, NaCl094 with Na/Cl atoms with $q = \pm 0.94e$.) For the force field MM+, there is no difference in geometry (and energy) when using different charge schemes for molecule or polarity for the substrate.

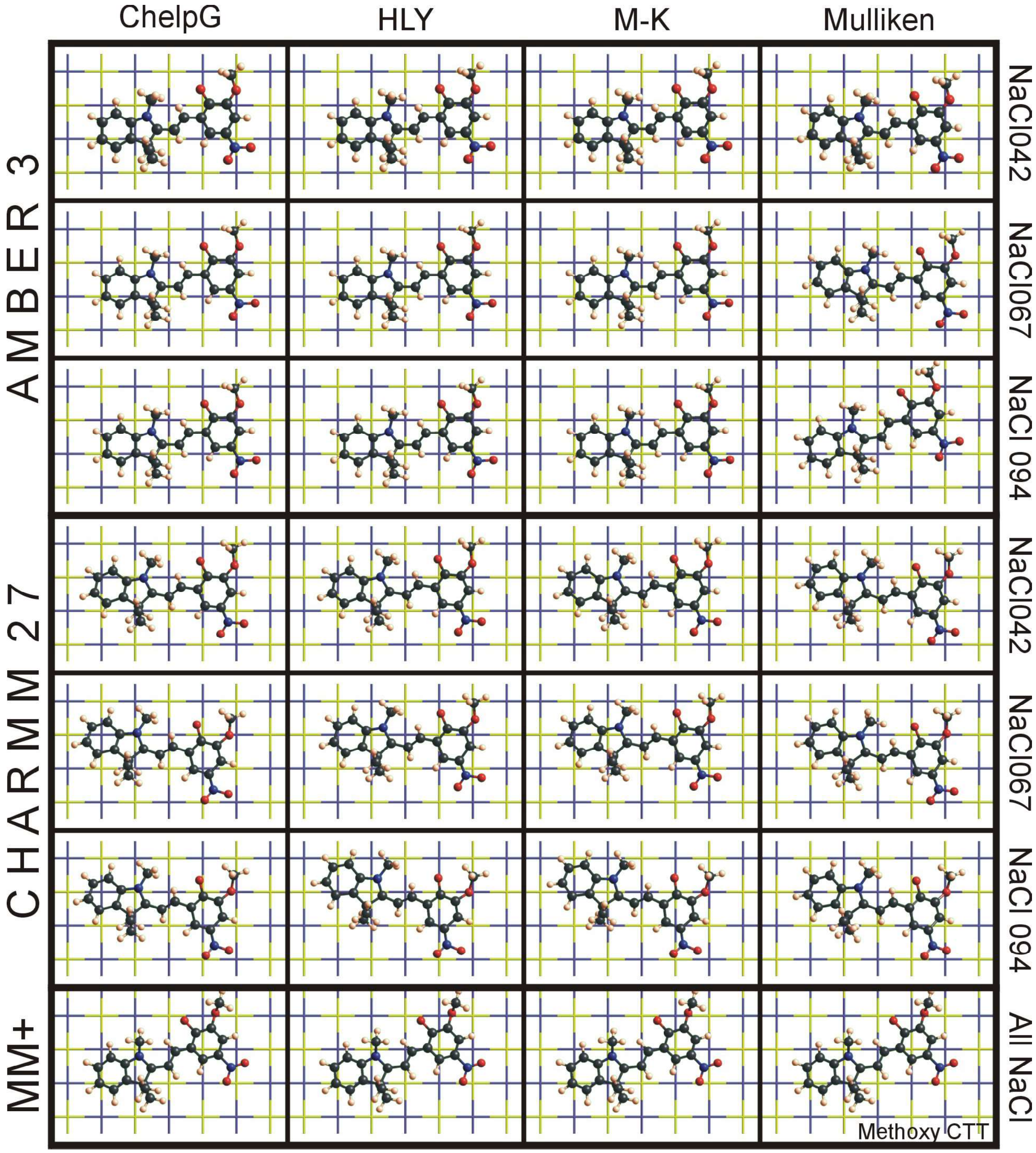

Figure S34: Adsorption geometry for **Methoxy CTT conformer** using four charge methods (ChelpG, HLY, M-K, and Mulliken) and three force fields, AMBER 3 (top three rows), CHARMM27 (row four to six), and MM+(bottom row) and three substrate polarities (NaCl042 with Na/Cl atoms with $q = \pm 0.42e$, NaCl067 with Na/Cl atoms with $q = \pm 0.67e$, NaCl094 with Na/Cl atoms with $q = \pm 0.94e$.) For the force field MM+, there is no difference in geometry (and energy) when using different charge schemes for molecule or polarity for the substrate.

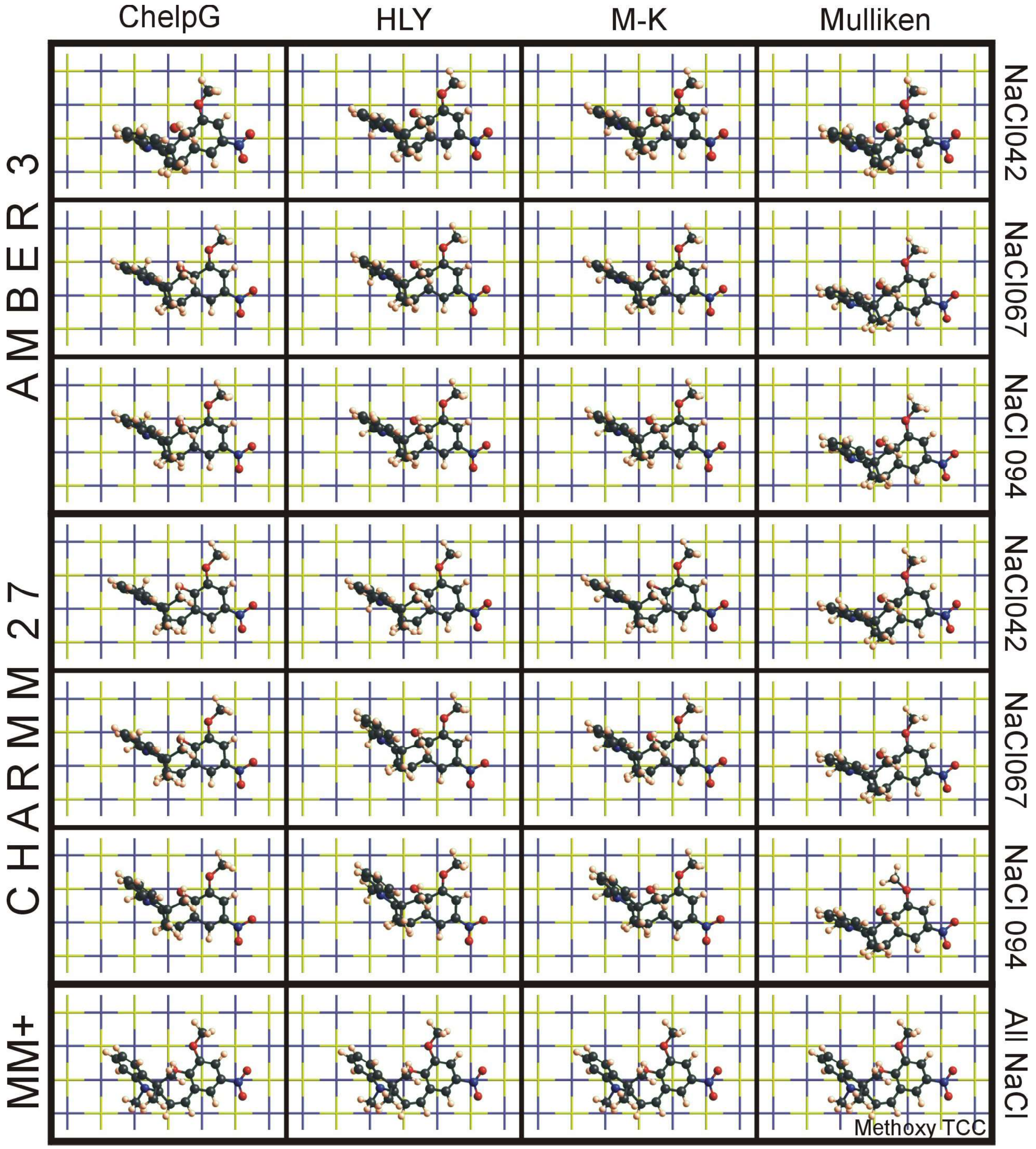

Figure S35: Adsorption geometry for **Methoxy TCC conformer** using four charge methods (ChelpG, HLY, M-K, and Mulliken) and three force fields, AMBER 3 (top three rows), CHARMM27 (row four to six), and MM+(bottom row) and three substrate polarities (NaCl042 with Na/Cl atoms with $q = \pm 0.42e$, NaCl067 with Na/Cl atoms with $q = \pm 0.67e$, NaCl094 with Na/Cl atoms with $q = \pm 0.94e$.) For the force field MM+, there is no difference in geometry (and energy) when using different charge schemes for molecule or polarity for the substrate.

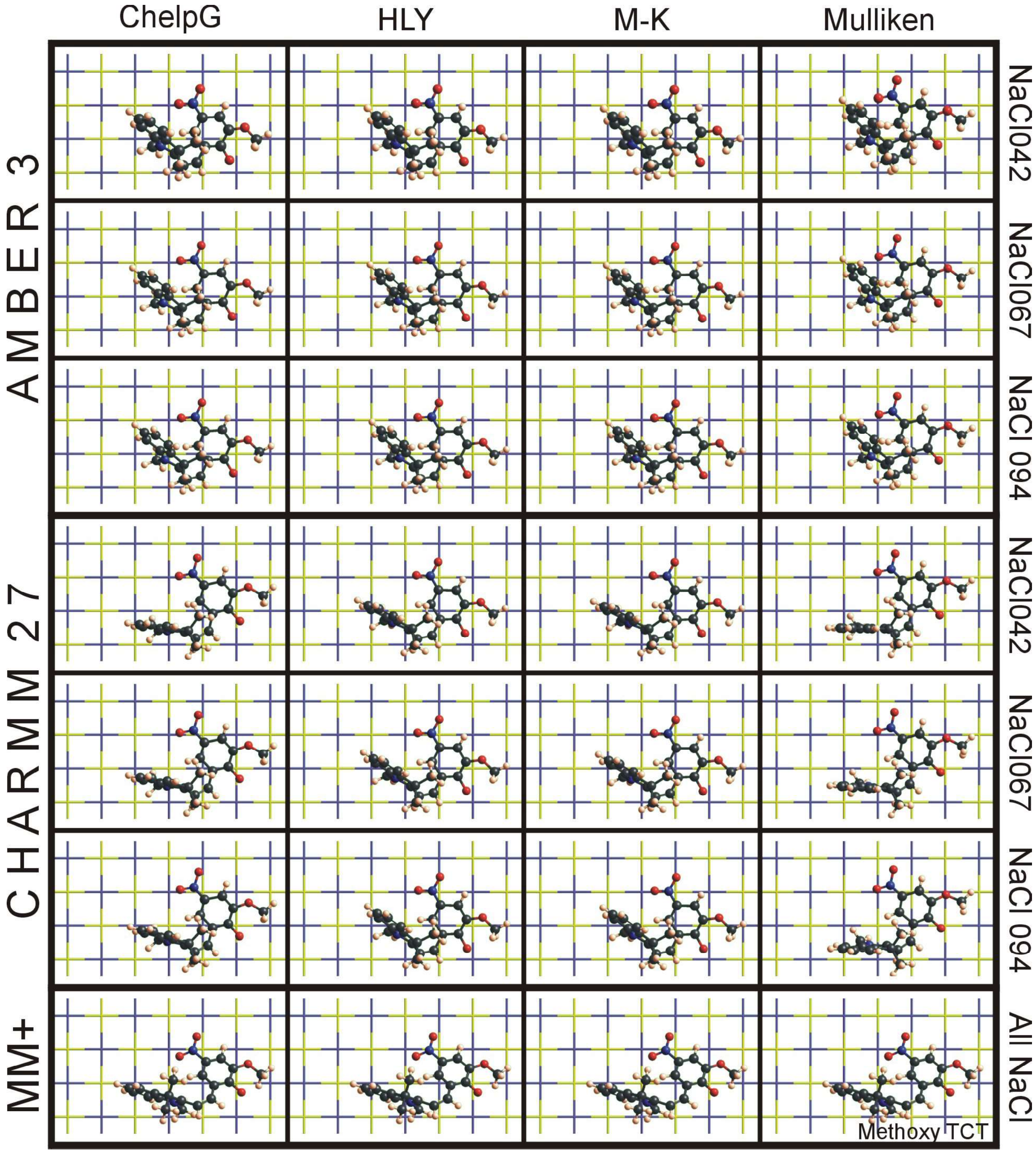

Figure S36: Adsorption geometry for **Methoxy TCT conformer** using four charge methods (ChelpG, HLY, M-K, and Mulliken) and three force fields, AMBER 3 (top three rows), CHARMM27 (row four to six), and MM+(bottom row) and three substrate polarities (NaCl042 with Na/Cl atoms with $q = \pm 0.42e$, NaCl067 with Na/Cl atoms with $q = \pm 0.67e$, NaCl094 with Na/Cl atoms with $q = \pm 0.94e$.) For the force field MM+, there is no difference in geometry (and energy) when using different charge schemes for molecule or polarity for the substrate.

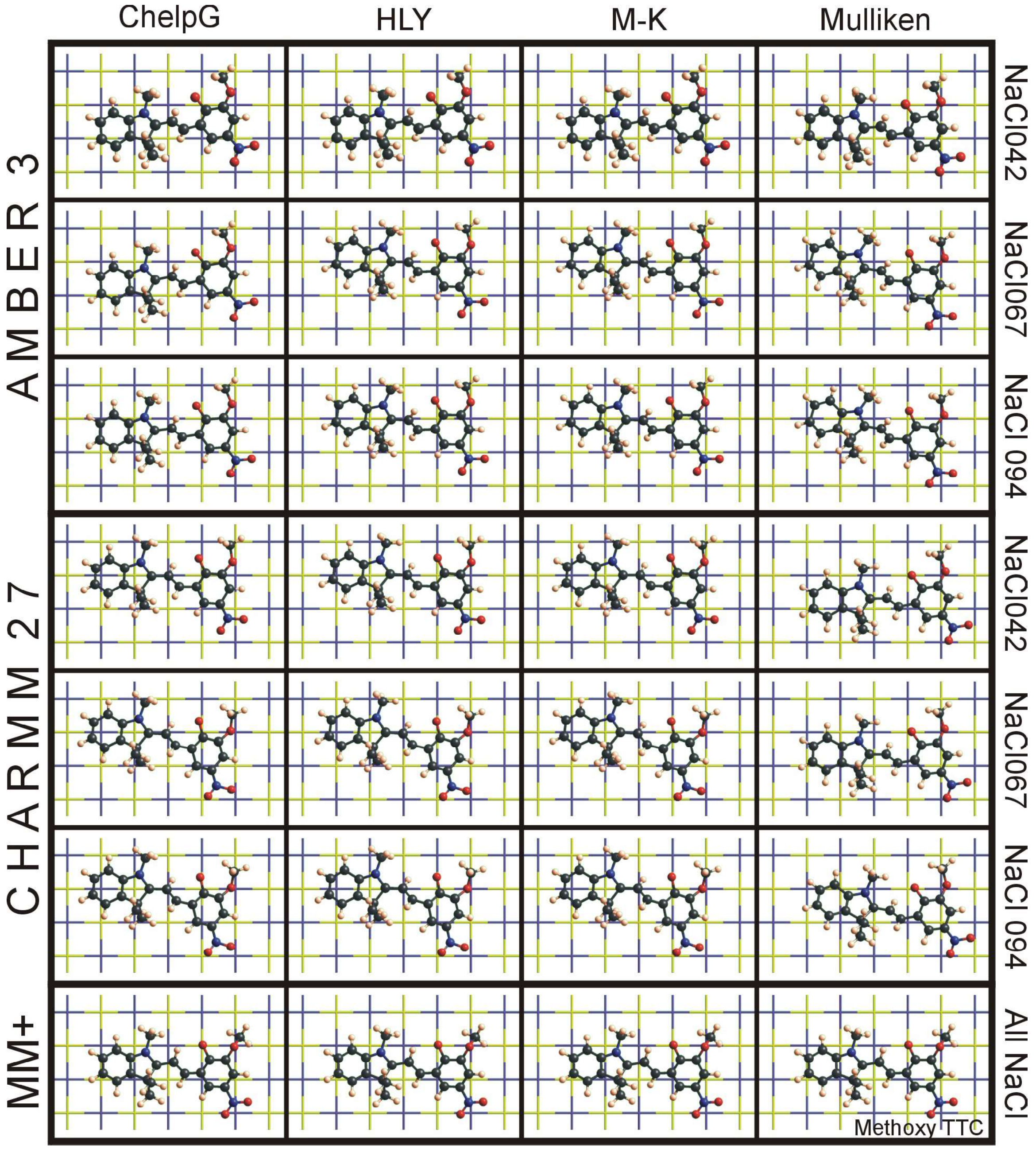

Figure S37: Adsorption geometry for **Methoxy TTC conformer** using four charge methods (ChelpG, HLY, M-K, and Mulliken) and three force fields, AMBER 3 (top three rows), CHARMM27 (row four to six), and MM+(bottom row) and three substrate polarities (NaCl042 with Na/Cl atoms with $q = \pm 0.42e$, NaCl067 with Na/Cl atoms with $q = \pm 0.67e$, NaCl094 with Na/Cl atoms with $q = \pm 0.94e$.) For the force field MM+, there is no difference in geometry (and energy) when using different charge schemes for molecule or polarity for the substrate.

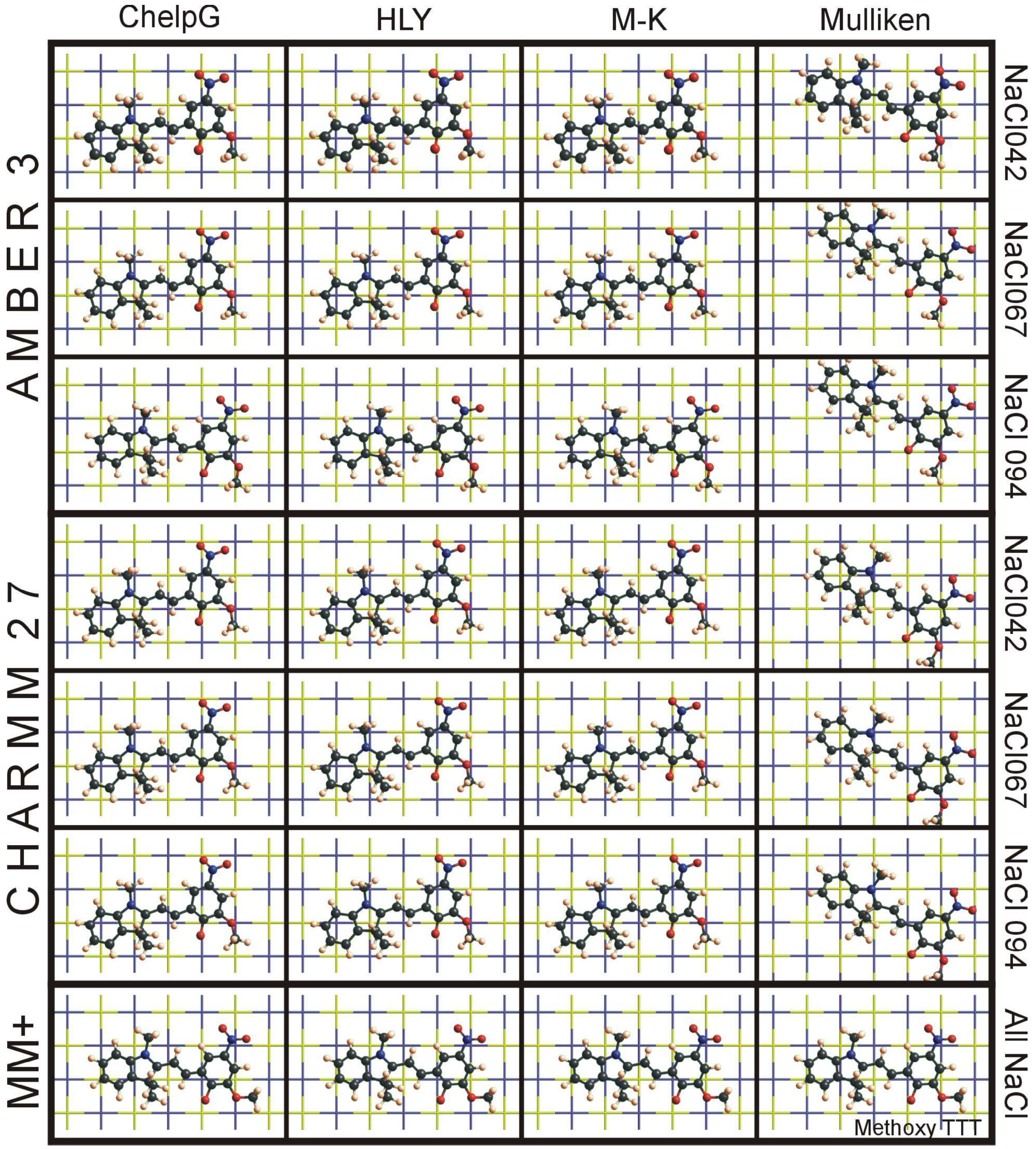

Figure S38: Adsorption geometry for **Methoxy TTT conformer** using four charge methods (ChelpG, HLY, M-K, and Mulliken) and three force fields, AMBER 3 (top three rows), CHARMM27 (row four to six), and MM+(bottom row) and three substrate polarities (NaCl042 with Na/Cl atoms with $q = \pm 0.42e$, NaCl067 with Na/Cl atoms with $q = \pm 0.67e$, NaCl094 with Na/Cl atoms with $q = \pm 0.94e$.) For the force field MM+, there is no difference in geometry (and energy) when using different charge schemes for molecule or polarity for the substrate.

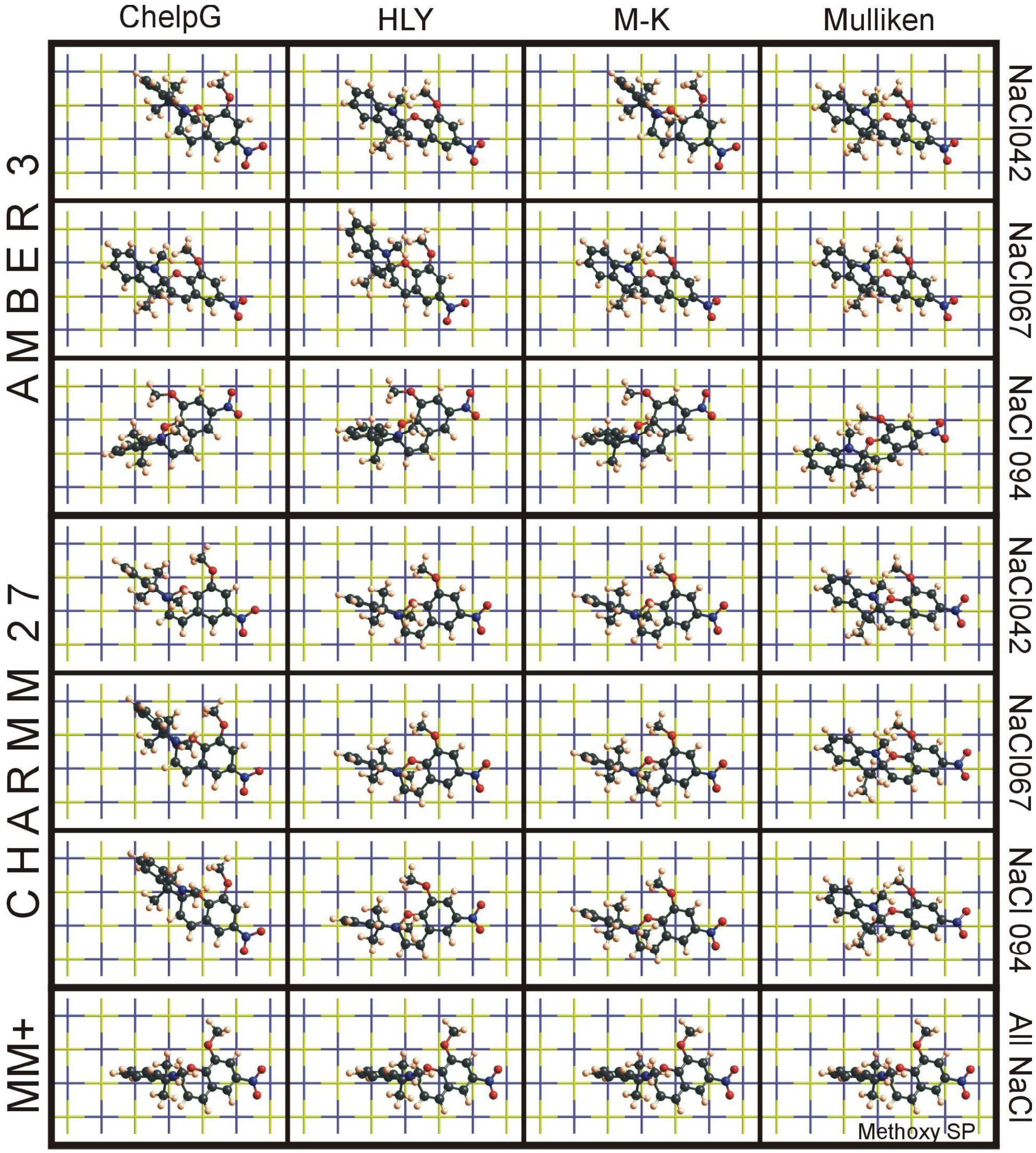

Figure S39: Adsorption geometry for **Methoxy SP isomer** using four charge methods (ChelpG, HLY, M-K, and Mulliken) and three force fields, AMBER 3 (top three rows), CHARMM27 (row four to six), and MM+(bottom row) and three substrate polarities (NaCl042 with Na/Cl atoms with $q = \pm 0.42e$, NaCl067 with Na/Cl atoms with $q = \pm 0.67e$, NaCl094 with Na/Cl atoms with $q = \pm 0.94e$.) For the force field MM+, there is no difference in geometry (and energy) when using different charge schemes for molecule or polarity for the substrate.

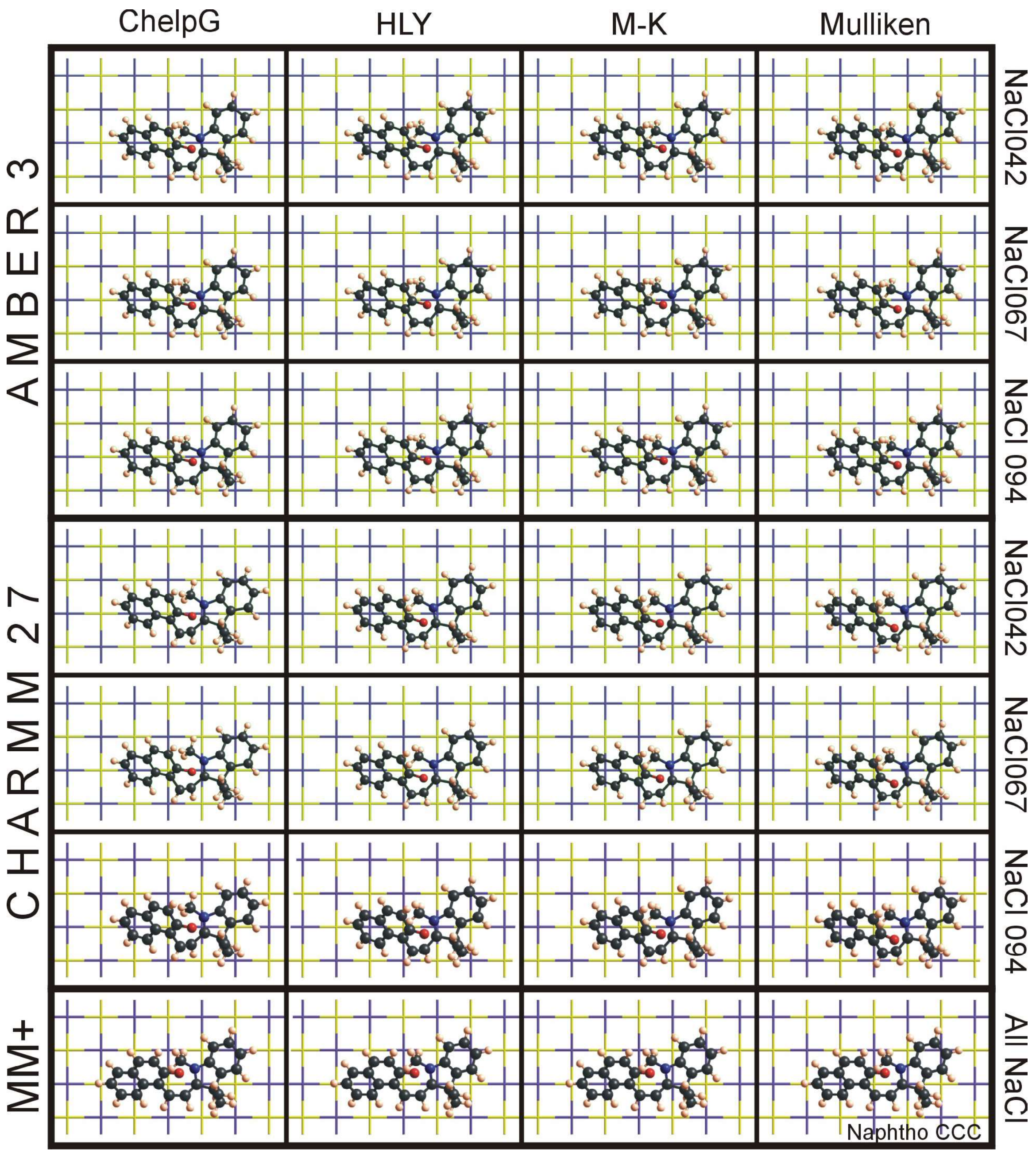

Figure S40: Adsorption geometry for **naphtho CCC conformer** using four charge methods (ChelpG, HLY, M-K, and Mulliken) and three force fields, AMBER 3 (top three rows), CHARMM27 (row four to six), and MM+(bottom row) and three substrate polarities (NaCl042 with Na/Cl atoms with $q = \pm 0.42e$, NaCl067 with Na/Cl atoms with $q = \pm 0.67e$, NaCl094 with Na/Cl atoms with $q = \pm 0.94e$.) For the force field MM+, there is no difference in geometry (and energy) when using different charge schemes for molecule or polarity for the substrate. The substrate's ionic bonds are rendered as tubes to show the grid: Chlorine (-) is yellow, and sodium (+) is blue/purple.

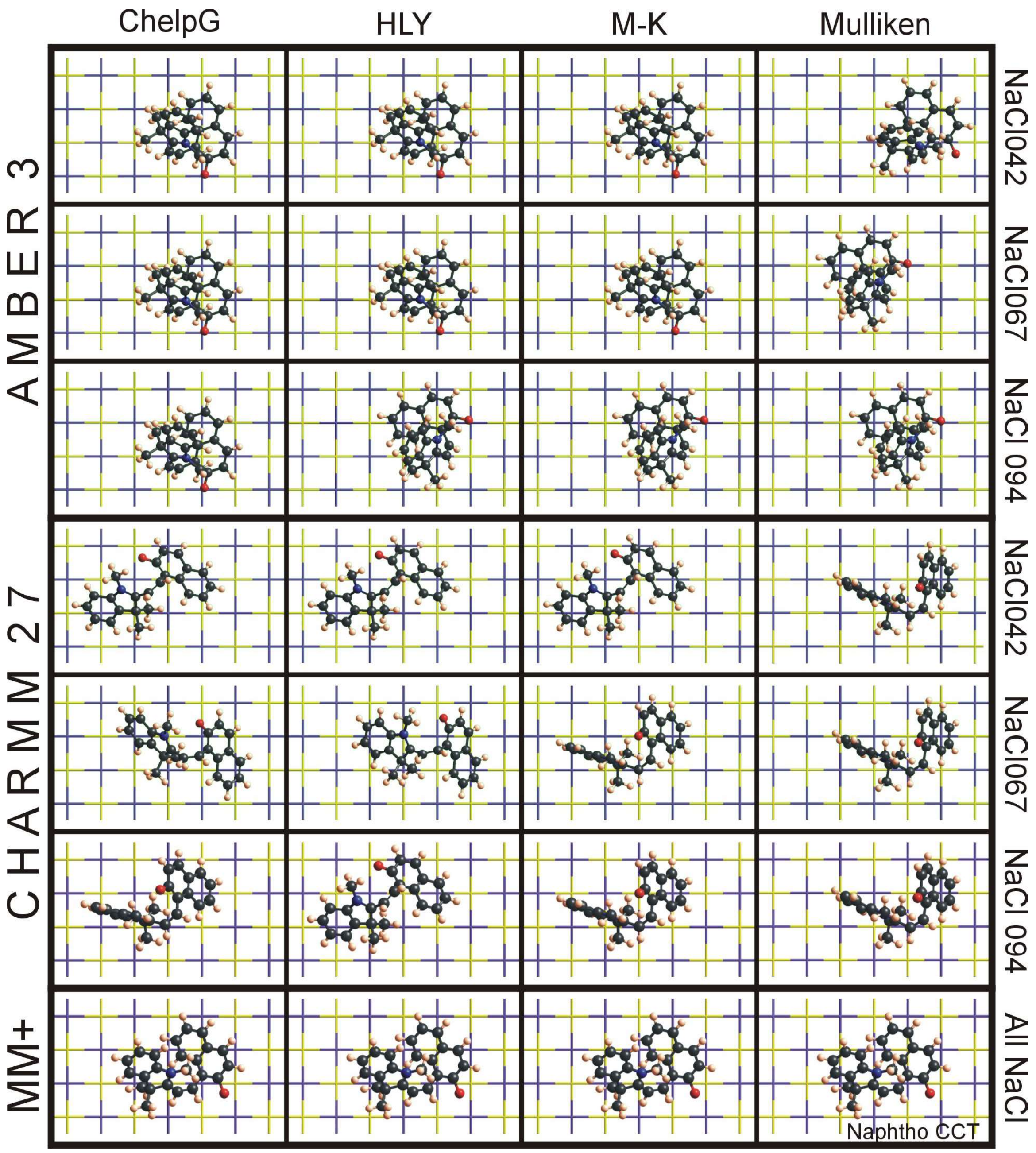

Figure S41: Adsorption geometry for **naphtho CCT conformer** using four charge methods (ChelpG, HLY, M-K, and Mulliken) and three force fields, AMBER 3 (top three rows), CHARMM27 (row four to six), and MM+(bottom row) and three substrate polarities (NaCl042 with Na/Cl atoms with $q = \pm 0.42e$, NaCl067 with Na/Cl atoms with $q = \pm 0.67e$, NaCl094 with Na/Cl atoms with $q = \pm 0.94e$.) For the force field MM+, there is no difference in geometry (and energy) when using different charge schemes for molecule or polarity for the substrate.

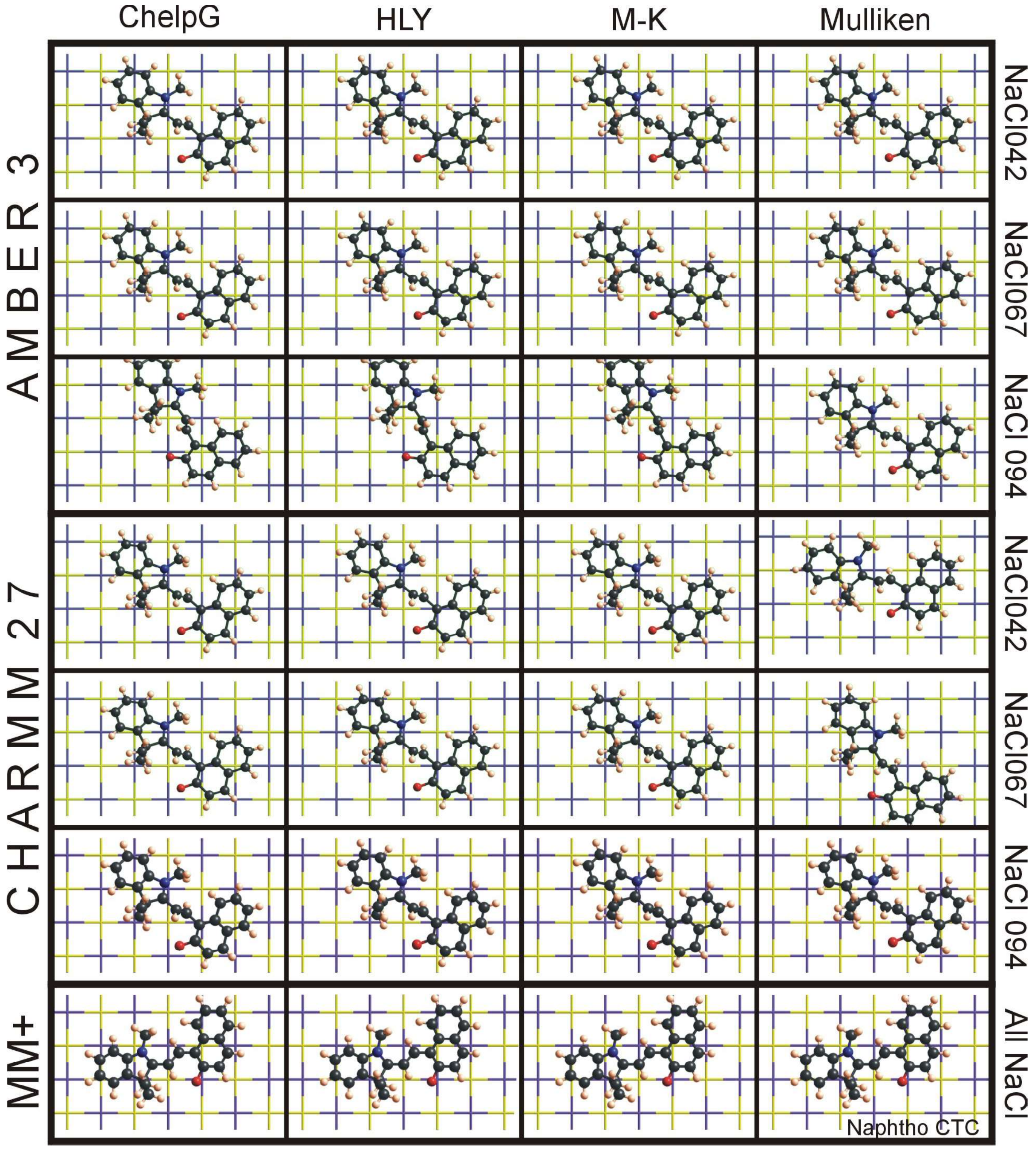

Figure S42: Adsorption geometry for **naphtho CTC conformer** using four charge methods (ChelpG, HLY, M-K, and Mulliken) and three force fields, AMBER 3 (top three rows), CHARMM27 (row four to six), and MM+(bottom row) and three substrate polarities (NaCl042 with Na/Cl atoms with $q = \pm 0.42e$, NaCl067 with Na/Cl atoms with $q = \pm 0.67e$, NaCl094 with Na/Cl atoms with $q = \pm 0.94e$.) For the force field MM+, there is no difference in geometry (and energy) when using different charge schemes for molecule or polarity for the substrate.

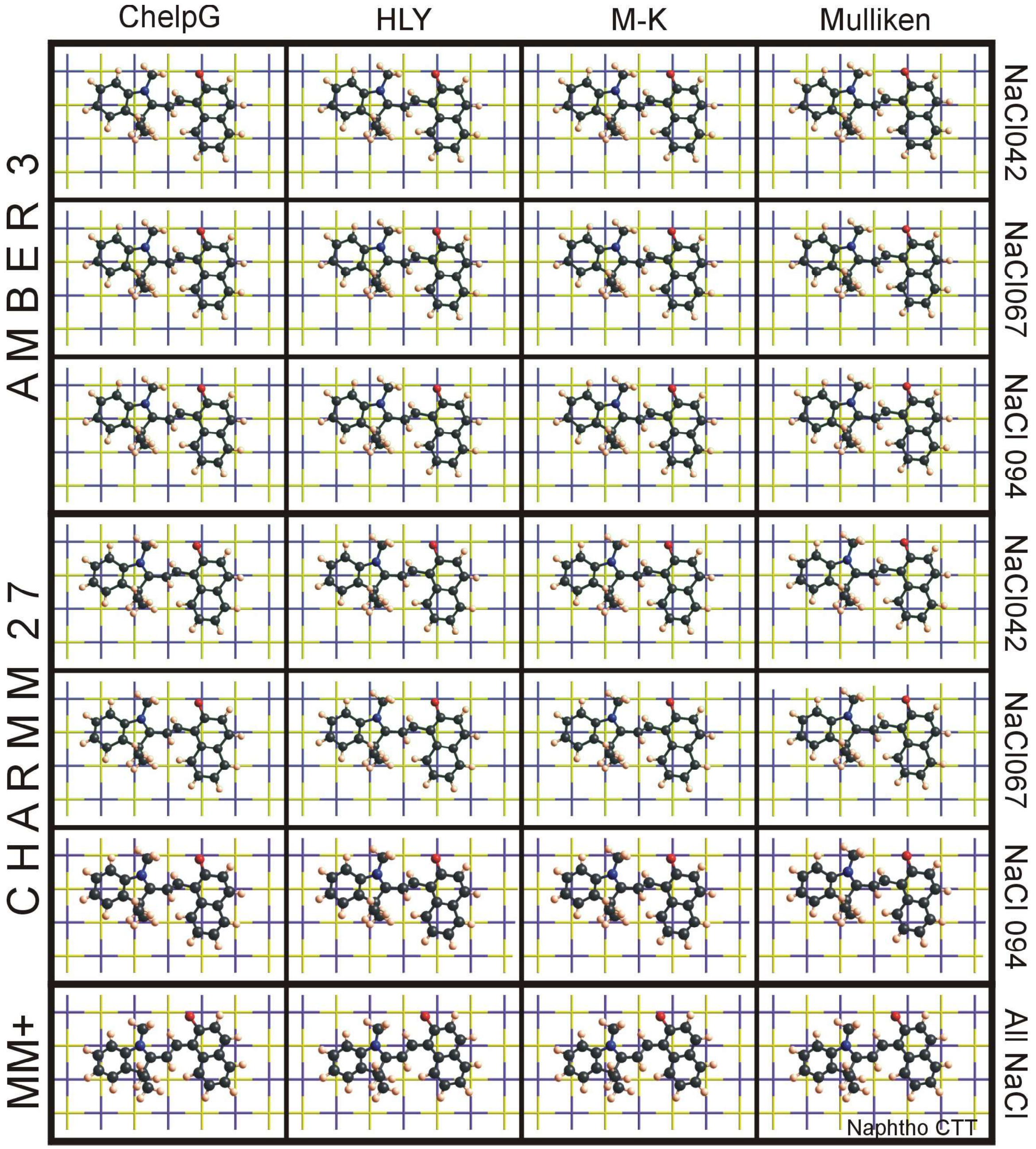

Figure S43: Adsorption geometry for **naphtho CTT conformer** using four charge methods (ChelpG, HLY, M-K, and Mulliken) and three force fields, AMBER 3 (top three rows), CHARMM27 (row four to six), and MM+(bottom row) and three substrate polarities (NaCl042 with Na/Cl atoms with $q = \pm 0.42e$, NaCl067 with Na/Cl atoms with $q = \pm 0.67e$, NaCl094 with Na/Cl atoms with $q = \pm 0.94e$.) For the force field MM+, there is no difference in geometry (and energy) when using different charge schemes for molecule or polarity for the substrate.

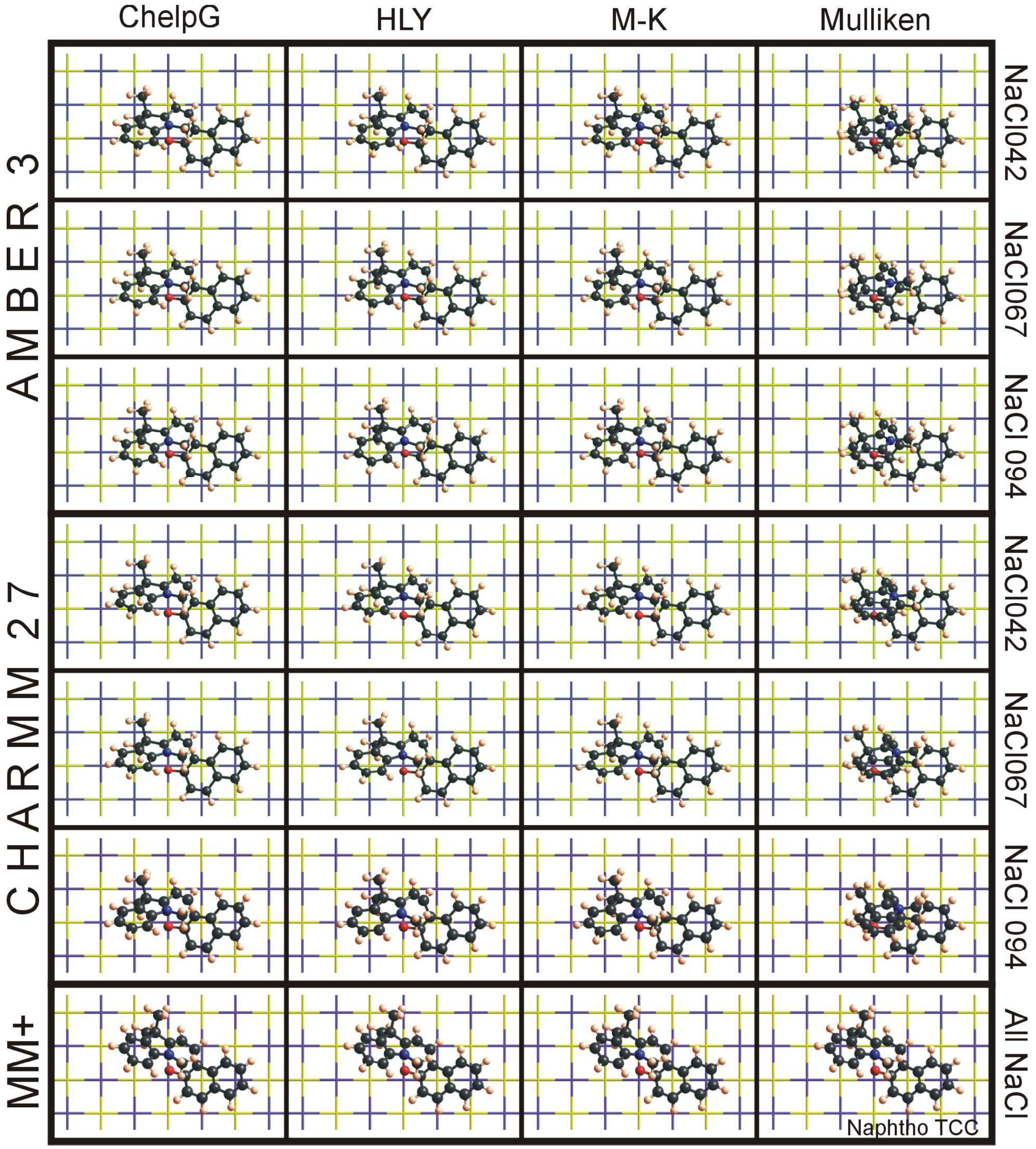

Figure S44: Adsorption geometry for **naphtho TCC conformer** using four charge methods (ChelpG, HLY, M-K, and Mulliken) and three force fields, AMBER 3 (top three rows), CHARMM27 (row four to six), and MM+(bottom row) and three substrate polarities (NaCl042 with Na/Cl atoms with $q = \pm 0.42e$, NaCl067 with Na/Cl atoms with $q = \pm 0.67e$, NaCl094 with Na/Cl atoms with $q = \pm 0.94e$.) For the force field MM+, there is no difference in geometry (and energy) when using different charge schemes for molecule or polarity for the substrate.

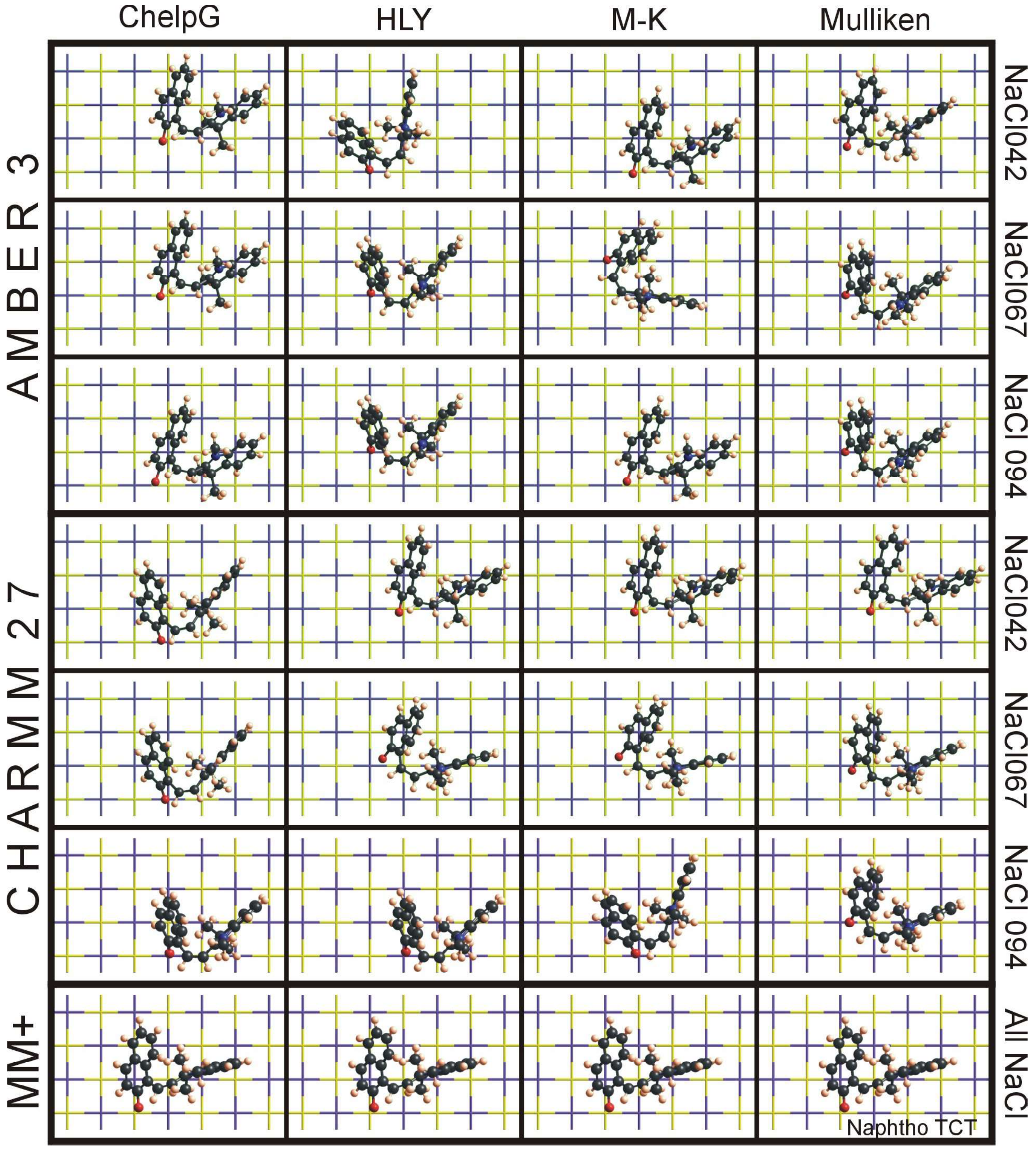

Figure S45: Adsorption geometry for **naphtho TCT conformer** using four charge methods (ChelpG, HLY, M-K, and Mulliken) and three force fields, AMBER 3 (top three rows), CHARMM27 (row four to six), and MM+(bottom row) and three substrate polarities (NaCl042 with Na/Cl atoms with $q = \pm 0.42e$, NaCl067 with Na/Cl atoms with $q = \pm 0.67e$, NaCl094 with Na/Cl atoms with $q = \pm 0.94e$.) For the force field MM+, there is no difference in geometry (and energy) when using different charge schemes for molecule or polarity for the substrate.

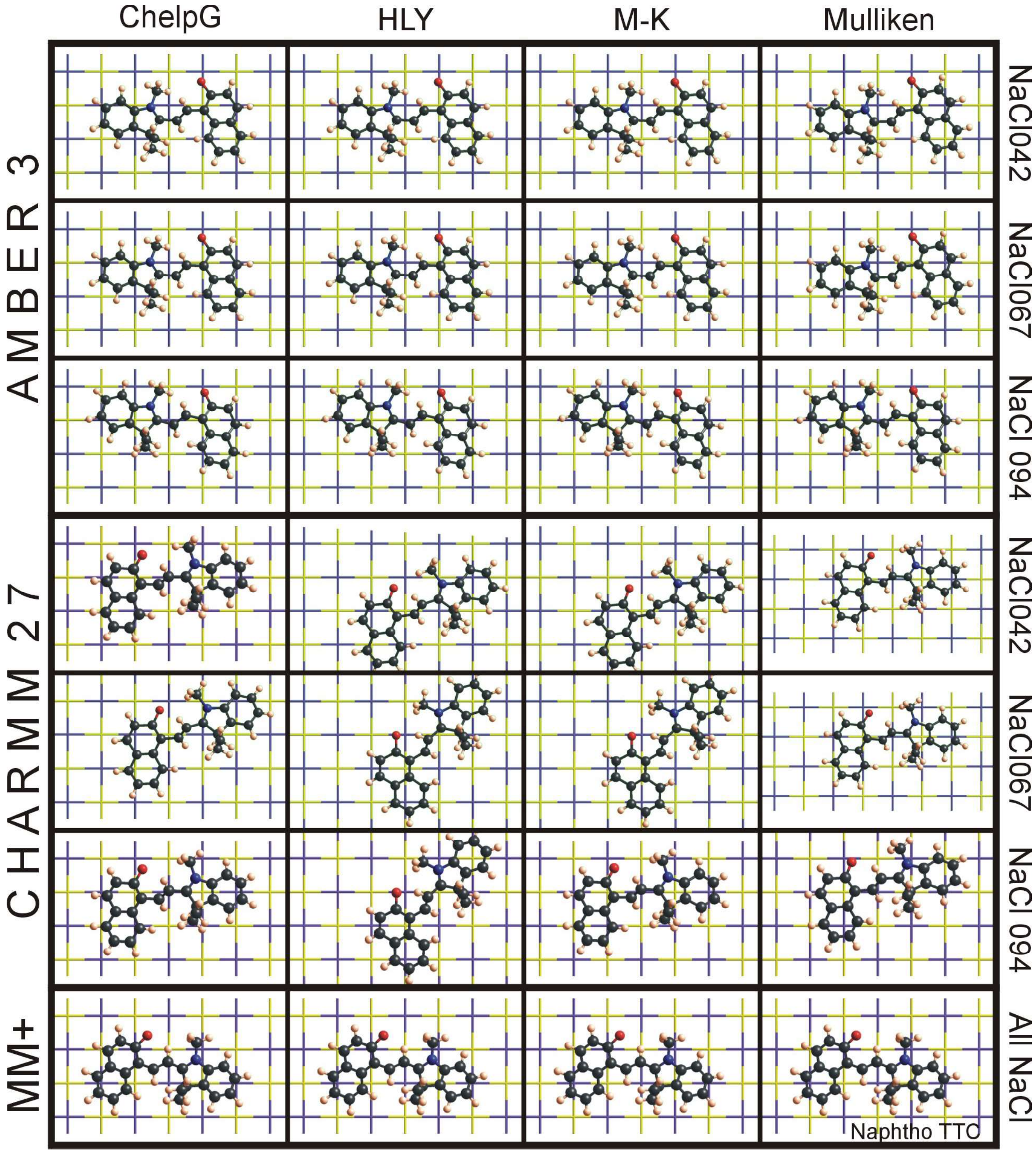

Figure S46: Adsorption geometry for **naphtho TTC conformer** using four charge methods (ChelpG, HLY, M-K, and Mulliken) and three force fields, AMBER 3 (top three rows), CHARMM27 (row four to six), and MM+(bottom row) and three substrate polarities (NaCl042 with Na/Cl atoms with $q = \pm 0.42e$, NaCl067 with Na/Cl atoms with $q = \pm 0.67e$, NaCl094 with Na/Cl atoms with $q = \pm 0.94e$.) For the force field MM+, there is no difference in geometry (and energy) when using different charge schemes for molecule or polarity for the substrate.

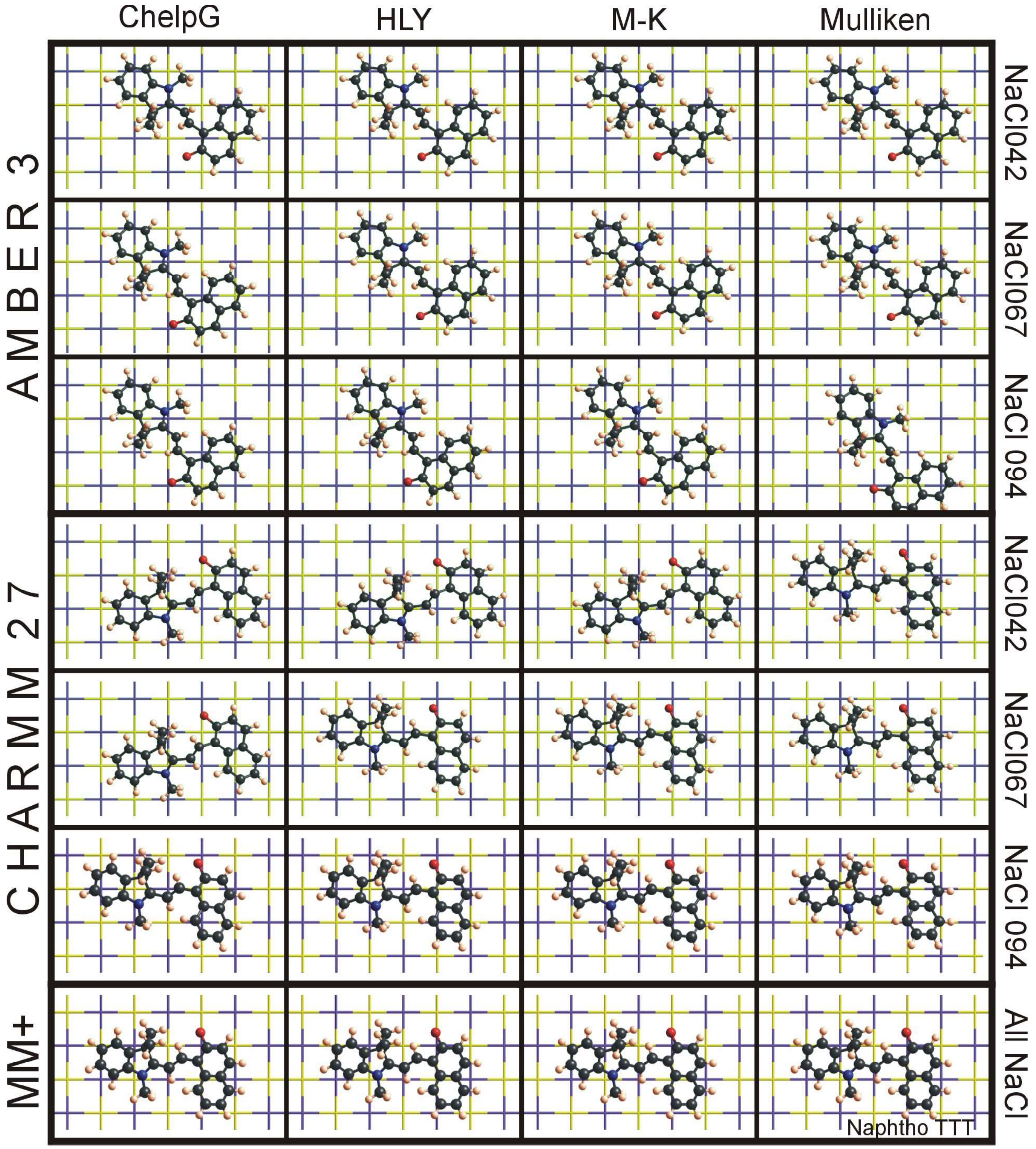

Figure S47: Adsorption geometry for **naphtho TTT conformer** using four charge methods (ChelpG, HLY, M-K, and Mulliken) and three force fields, AMBER 3 (top three rows), CHARMM27 (row four to six), and MM+(bottom row) and three substrate polarities (NaCl042 with Na/Cl atoms with $q = \pm 0.42e$, NaCl067 with Na/Cl atoms with $q = \pm 0.67e$, NaCl094 with Na/Cl atoms with $q = \pm 0.94e$.) For the force field MM+, there is no difference in geometry (and energy) when using different charge schemes for molecule or polarity for the substrate.

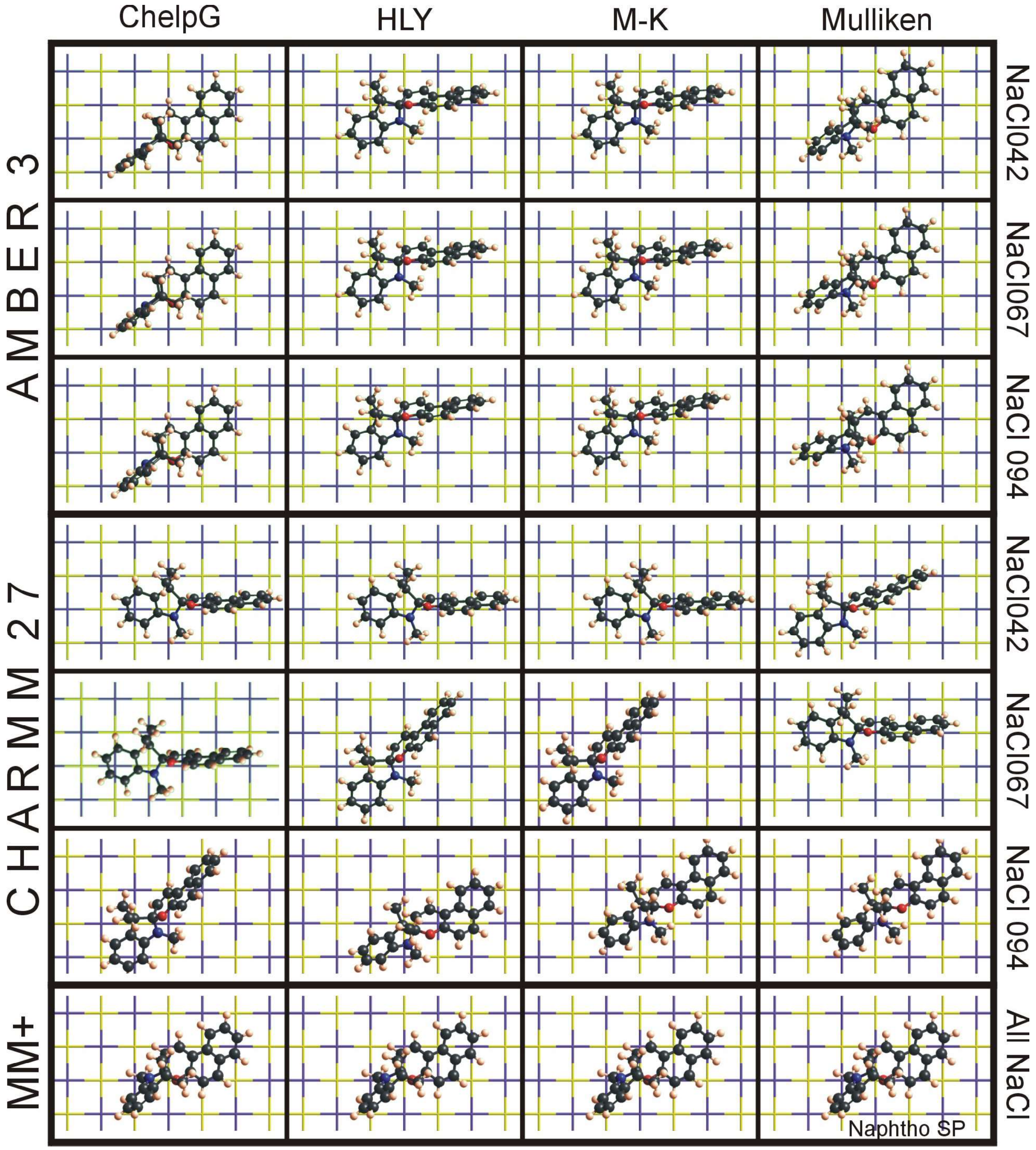

Figure S48: Adsorption geometry for **naphtho SP isomer** using four charge methods (ChelpG, HLY, M-K, and Mulliken) and three force fields, AMBER 3 (top three rows), CHARMM27 (row four to six), and MM+(bottom row) and three substrate polarities (NaCl042 with Na/Cl atoms with $q = \pm 0.42e$, NaCl067 with Na/Cl atoms with $q = \pm 0.67e$, NaCl094 with Na/Cl atoms with $q = \pm 0.94e$.) For the force field MM+, there is no difference in geometry (and energy) when using different charge schemes for molecule or polarity for the substrate.
